# Evaluation of using small volume of interest regions for clinical kidney dosimetry in ^177^Lu-DOTATATE treatments

**DOI:** 10.1186/s40658-025-00769-w

**Published:** 2025-07-08

**Authors:** Jehangir Khan, Tobias Rydèn, Martijn Van Essen, Johanna Svensson, Peter Bernhardt

**Affiliations:** 1 Department of Medical Physics and Biomedical Engineering (MFT), Sahlgrenska University Hospital, Gothenburg, SE-41345 Sweden; 2 Department of Medical Physics, Faculty of Medicine and Health, Örebro University Hospital, Örebro, Sweden; 3 Department of Clinical Physiology, Sahlgrenska University Hospital, Gothenburg, Sweden; 4 Department of Oncology, Institution of Clinical Sciences, Sahlgrenska Academy at University of Gothenburg, Gothenburg, Sweden; 5 Department of Medical Radiation Sciences, Institute of Clinical Sciences, Sahlgrenska Academy at University of Gothenburg, Gothenburg, Sweden

**Keywords:** ^177^Lu-DOTATATE, Neuroendocrine, Single-photon emission tomography, SPECT/CT, Kidney dosimetry, Recovery coefficient

## Abstract

Segmentation of the whole-kidney parenchyma (WKP) is considered the reference method for kidney dosimetry of radiopharmaceuticals, as it provides the average absorbed dose to the fully delineated WKP. However manual segmentation of the WKP is time consuming, and automated segmentation requires operator verification and potential manual adjustments to the VOI. The aim is to determine if a small volume of interest (SV) method can generate similar kidney absorbed doses as the WKP method. Methods: We obtained SPECT/CT of 18 patients at 24, 48, and 168 h after injection of [^177^Lu]Lu-DOTATATE (7.3–7.8 GBq). The SPECTs were corrected for attenuation, scatter, and collimator detector response with Monte Carlo-based OSEM reconstruction (ASCC-SPECT) and post-filtered with a 0- to 12-mm Gaussian filter or were only attenuation corrected with a Hann post-filter (AC-SPECT). Kidney dosimetry based on the manually segmented WKP was used as reference method. Recovery coefficients (RCs) for each WKP were determined by Monte Carlo simulations, and normalisation factors, NFs, for SVs were determined relative to the WKP method. Kidney absorbed doses were estimated based on measured activity concentrations fitted using the mono-exponential function. The accuracy of the absorbed dose estimates for the SV methods, corrected with the NFs, were assessed using the standard deviation of the percentage difference in agreement with the reference method across all kidneys. Accuracy for kidney dosimetry using the SV method was calculated based on 1–5 VOIs with volumes of 4 mL (SV_4_), 2 mL (SV_2_), and 0.6 mL (SV_0.6_). Results: The mean RCs of the WKP volumes (31–243 mL) in non-filtered ASCC-SPECT and AC-SPECT were 0.85 (0.73–0.90) and 0.62 (0.46–0.51), respectively. In non-filtered images, the absorbed dose was overestimated by a factor of 1.22. However, applying a Gaussian filter with a kernel size of approximately 5 mm yielded absorbed dose estimates comparable to the reference WKP method. The accuracy of kidney dosimetry calculation based on one SV_4_ on each SPECT data-point was 12%. The accuracy improved as the number of VOIs increased from 1 to 5. With the SV_2_ method, using a mean of 5 VOIs per kidney parenchyma, the accuracy was 8.3%. Conclusion: The small volume of interest (SV) method can provide absorbed dose estimates comparable to the whole-kidney parenchyma (WKP) method when optimized. Non-filtered images overestimated doses by 1.22, but applying a 5 mm Gaussian filter aligned SV results with the WKP method. Using multiple VOIs improved accuracy, with five 2 mL SVs achieving 8.3%. The SV method provides a less time-consuming alternative to WKP; however, its implementation is recommended to be validated and adjusted against a reference method.

**Supplementary Information:**

The online version contains supplementary material available at 10.1186/s40658-025-00769-w.

## Introduction

Therapy using radiolabeled somatostatin analogues, e.g., [^177^Lu]Lu-DOTA-TATE, is an established option for the treatment of metastasized or inoperable neuroendocrine tumours (NETs) [1]. Because, the kidney is considered dose limiting for [^177^Lu]Lu-DOTA-TATE [2] it is desirable to perform individualized kidney dosimetry to improve treatment efficacy [3, 4] and define more accurate threshold absorbed doses [5, 6]. However, accurate kidney dosimetry depends on the quantitative analysis of multiple SPECT images, which typically require time-consuming manual segmentation of volumes of interest (VOI) or the use of automated segmentation, often AI-based, now introduced by some commercial software. Despite advancements, automated segmentation still requires operator verification and potential manual adjustments to the VOI.

To reduce manual segmentation time for the whole kidney parenchyma (WKP), Sandström et al. [2, 7] introduced the small VOI method (SV), in which a 4-mL spherical SV (SV_4_) is positioned at the highest uptake location in the kidney parenchyma. Using low-resolution SPECT images— specifically, attenuation-corrected OSEM reconstruction SPECT with post-filtering (AC-SPECT)—a linear correlation has been achieved between the absorbed doses estimated from SV and the manually segmented WKP. With this SPECT protocol, the SV method underestimates the absorbed dose for the right and left kidneys by median of 4.5% and 3.9%, respectively, compared to the manually segmented WKP for the patient cohort. The largest deviations between the methods were 25% for the right kidney and 24.8% for the left kidney [8].

Heikkonen et al. [9] applied the SV method using high-resolution SPECT—i.e., attenuation-, scatter-, and collimator detector-corrected OSEM reconstruction SPECT (ASCC-SPECT)—which resulted in overestimation of the WKP dose by a factor of 1.7 (range: 1.3–2.2). Hou et al. [10] asserted that neither Sandström et al. [2] nor Heikkonen et al. [9] had adjusted the WKP method for partial volume effects (PVEs). To account for PVEs, Hou et al. [10] applied a dual iterative adaptive thresholding technique to estimate the activity concentration in WKP. With high-resolution SPECTs, SV/WK ratios of 1.8 ± 0.6 and 1.7 ± 0.5 were obtained for right and left kidneys, respectively. The linear correlation coefficient was between 0.7 and 0.9. Recently, a smaller VOI with a diameter of 0.6 mL was applied not at the highest uptake region, but rather in a visually representative uptake region of the kidney parenchyma [11]. That study did not include any comparison with manually delineated WKP. The above-described studies demonstrate that the SV method can be useful and may potentially be improved, and that further evaluation is warranted.

In the present study, we aimed to analyze four different aspects that have the potential to improve the SV method. First, it may be possible to adjust the diameter of the spherical SV in accordance with size of the kidney parenchyma. The mean thickness of the kidney parenchyma is often less than the diameter of the SV_4_ because parenchyma thickness decreases with age and is affected by prior treatments [12, 13]. Second, the SV approach is used to estimate the mean activity concentrations within the kidney; therefore, it seems more rational to place the SV in a more representative location than the highest uptake region of the kidney. Third, more smooth SPECT image quality, compared to high-resolution SPECT, might be advantageous for obtaining representative SV measurement of the mean activity concentration. Finally, greater accuracy may be achieved by using several SVs placed at different representative locations compared to one single SV.

## Materials and methods

This retrospective study was based on 18 NETs patients who were enrolled for [^177^Lu]Lu-DOTA-TATE (Lutathera^®^, AAA) treatment at Sahlgrenska University Hospital. The patients included 8 men and 10 women, with an average age of 71 years (range: 56–86 years). The Swedish Ethics Review Board approved the study, waiving the requirement of consent to participate (2020–05092). SPECT data were acquired between 2019 and 2021 at Sahlgrenska University Hospital. For each patient, infusion of amino acid solution (Vamin^®^ 14 gN/L. 2000 mL Fresenius Kabi AG, Bad Homburg, Germany) was started 30 min prior to ^177^Lu-DOTATATE administration with an infusion speed of 400 mL/h. A total of 2 L amino acid solution was administered to each patient per treatment cycle to prevent nephrotoxicity. SPECT/CT acquisitions were performed at 24, 48, and 168 h after ^177^Lu-DOTATATE injection.

### Data acquisition and image reconstruction

Imaging data were acquired using the SPECT/CT system Discovery NM/CT 670 (GE healthcare, Waukesha, WI, USA), with a NaI (Tl) crystal thickness of 5/8” and a medium-energy parallel-hole collimator. A 20% energy window around the 208-keV photon peak was used for SPECT examinations. The lower scattering window was between 177.8 and 187.2 keV and the upper scattering window was between 228.8 and 240.2 keV. The SPECT/CT examinations were performed over the upper abdomen, including the kidneys, with an auto-contour system, using step-and-shoot mode with a clockwise and circular orbit. At a rate of 30 s per projection, a total of 120 projections were acquired, with a matrix size of 128 × 128, and a pixel size and slice thickness of 4.42 mm. For the CT, matrix size was 512 × 512, slice thickness was 5 mm, and pixel size was 0.98 mm.

SPECT data were reconstructed using the in-house PhONSAi 4.0 image platform [14]. Attenuation-, scatter-, and collimator detector-corrected ordered subset expectation maximization reconstructions (ASCC-OSEMs) were obtained using the Monte-Carlo code SARec [15]. The reconstructions were performed with 10 subsets and 6 iterations. To obtain different SPECT resolutions, Gaussian post-filtering was applied (sigma between 0 and 12 mm), where sigma denotes the standard deviation (σ). We also applied the low-resolution SPECT reconstruction protocol used by Sandström et al. [2, 7]—i.e., attenuation-corrected OSEM with 4 iterations and 8 subsets, post-filtered with a Hann filter with a cut-off of 0.85 (AC-OSEM).

### Kidney delineation and activity quantification

The activity concentration in segmented whole-kidney parenchyma volume (WKP), was considered as reference, i.e., used as the reference segmentation method and the reference activity measurement method. The segmented WKP - including the renal cortex and medulla, and excluding the renal pelvis was manually delineated on the CT images for all SPECT/CT acquisitions performed at 24 h, 48 h, and 168 h after ^177^Lu-DOTATATE injection. The delineated WKP was manually adjusted to fit the position of the kidney in the SPECT, which can differ from the CT position due to organ movements [16].

### Recovery coefficients for WKP

For each WKP at all time points, the recovery coefficient (RC) was determined by Monte Carlo simulations, using the SARec code [15]. The CT was used as a digital phantom, in which the WKPs of the left and right kidney was the source organs in the simulations. A fixed simulated activity, defined as (A_true, sim_) was uniformly distributed in the WKPs. All remaining organs in the CT based digital phantom had no activity. One hundred and twenty (120) SPECT projections around the CT phantom, were used in the SPECT reconstructions of the digital phantom. The SPECT acquisition parameters were the same as described above. The RC was determined as the ratio of the measured WKP activity in the simulated SPECT (A_measured, sim_) and A_true_.

To describe the general trend of the RCs versus volume, a non-linear function was fitted to the RCs using Eq. 1:

1 $$\:RC\left(V\right)=\frac{1}{{\left(\:1+\frac{\text{a}}{\text{V}}\right)}^{b}}$$

where a, and b are the parameters to fit, and V is the volume of the WKP.

### Activity concentration determination

The activity concentration (MBq/g) for each VOI (V) and time-point on patient SPECT images was determined using Eq. 2:

2 $$\:C\left(t\right)=\frac{\text{c}\left(\text{t}\right)}{\text{Q}\:\times\:\:\text{R}\text{C}\left(\text{V}\right)\:\times\:\:\:\text{V}\:\times\:\:{\uprho\:}\:}$$

Where c(t) is the counts per second (cps) in the VOI, Q is the SPECT calibration factor (cps/MBq) for the specific camera, reconstruction and post-filtering protocol used, ρ is the density of the kidney parenchyma (1.05 g/cm^3^), V represents the volume of the manually segmented WKP from CT imaging, and RC(V) is the recovery coefficient determined based on the segmented CT-based WKP, accounting for partial volume effects and system-related factors. The SPECT calibration factor for the WKP and SV methods was determined by filling a Jaszczak phantom with known activity and measured the counts in a large VOI centered inside the SPECT reconstructed phantom. The counts were divided by the known activity within the VOI volume for obtaining Q.

**Fig. 1:**
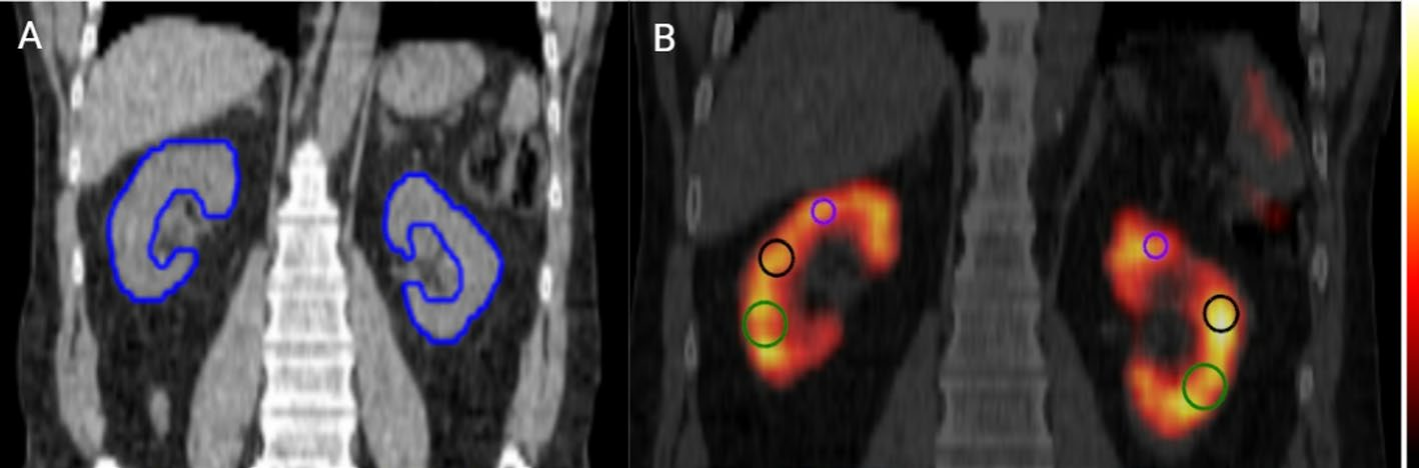
(**A**) The CT image with manually delineated whole-kidney parenchyma (WKP) volumes (blue). (**B**) ASCC-SPECT/CT image without post-filter, showing examples of typical locations of SV_4_ (green), SV_2_ (black), and SV_0.6_ (purple) in the kidney parenchyma

For evaluation of the small VOI method, up to 5 SVs—with diameters of 4 mL (SV_4_), 2 mL (SV_2_), or 0.6 mL (SV_0.6_)—were placed in regions aiming to have a radioactivity distribution representative of the whole kidney (Fig. 1). Specifically, they were located toward the kidney pelvis and at a slight distance from the outer cortex region. The activity concentration in each SV was determined according to Eq. 2, excluding the RC.

### Absorbed dose calculation

The time-integrated activity concentration (TIAC) was computed from time activity concentration curves obtained from a mono-exponential fit, integrated from time zero to infinity. The kidney absorbed doses were calculated for the WKP (D_WKP_) and SV (D_SV_) methods, assuming local energy deposit (LED) of the emitted electrons, using Eq. 3.

3 $$\:{D}_{m}=\text{T}\text{I}\text{A}\text{C}\times\:\:\text{L}\text{E}\text{D}$$

Where m indicates the segmentation method (WKP, SV_4_, SV_2_, or SV_0.6_) and LED is the average electron energy (147 keV) per disintegration emitted by ^177^Lu. This calculation omitted the self-absorption and cross-irradiation of photons from other organs.

### Normalisation factors for the small VOI methods

The normalization factor for the SV methods (NF_SV_) were estimated in relation to the WKP method. Three different (NF_SV_) factors were determined for three distinct SV volumes (V_SV_), using Eq. 4:

4 $$\:{NF}_{SV}\left({V}_{SV}\right)=\frac{\sum\:_{k=1}^{n}\frac{{D}_{SV}{(V}_{SV})}{{D}_{WKP}}}{n}$$

Where n is the number of evaluated kidneys for all single SVs, i.e. n is equal to 36 kidneys times 5 SVs making NF_SV_ the mean of 180 data points. The NFsv was applied as an apparent RC factor for the SV method. By dividing D_SV_ by NFsv, the normalized absorbed dose D_svn_ was obtained.

### Accuracy Estimation based on published absorbed dose data

To evaluate the accuracy in kidney absorbed dose calculations between the WKP method and the small VOI approach, data from Sandström et al. (2010) were re-analzsed. Specifically, accuracy was assessed by calculating the standard deviation of the differences in absorbed dose estimates between large VOI and SV of 4 mL for the right and left kidneys, using previously published data available in the cited publication [7]. These differences were normalized to the absorbed dose calculated using the large VOI for each kidney. While this specific analysis was not included in the original publication, it was performed in our study to quantify the impact of VOI size on absorbed dose estimates using published data.

### Statistical analysis

Statistical analysis was performed using MATLAB (The MathWorks, Inc; Natick, MA, USA). The correlation of the kidney absorbed dose estimate between the WKP and SV methods was evaluated using the Pearson correlation coefficient. Bland-Altman plots were used to analyze the agreement of the kidney absorbed dose rate estimate by evaluating the bias and accuracy between the SVs methods and the WKP method that was considered as the reference method. The bias and accuracy of the absorbed dose rate estimates for the SV methods were assessed using the mean and standard deviation of the percentage difference (PD) in agreement with the reference method across all kidneys. To assess the precision and variability of the measured WKP RCs, the Coefficient of variation (COV) was calculated. The COV was determined as the ratio of the standard deviation by the mean of the measurements, and the result was reported as a percentage. The uncertainty in the absorbed dose rate estimate with WKP is unknown but will be further investigated in a future study.

5 $$\:PD=\frac{{D}_{SVN}-\:{D}_{WKP}\:}{{D}_{WKP}}\times\:100$$

## Results

### Recovery coefficients for manually delineated kidneys

**Fig. 2:**
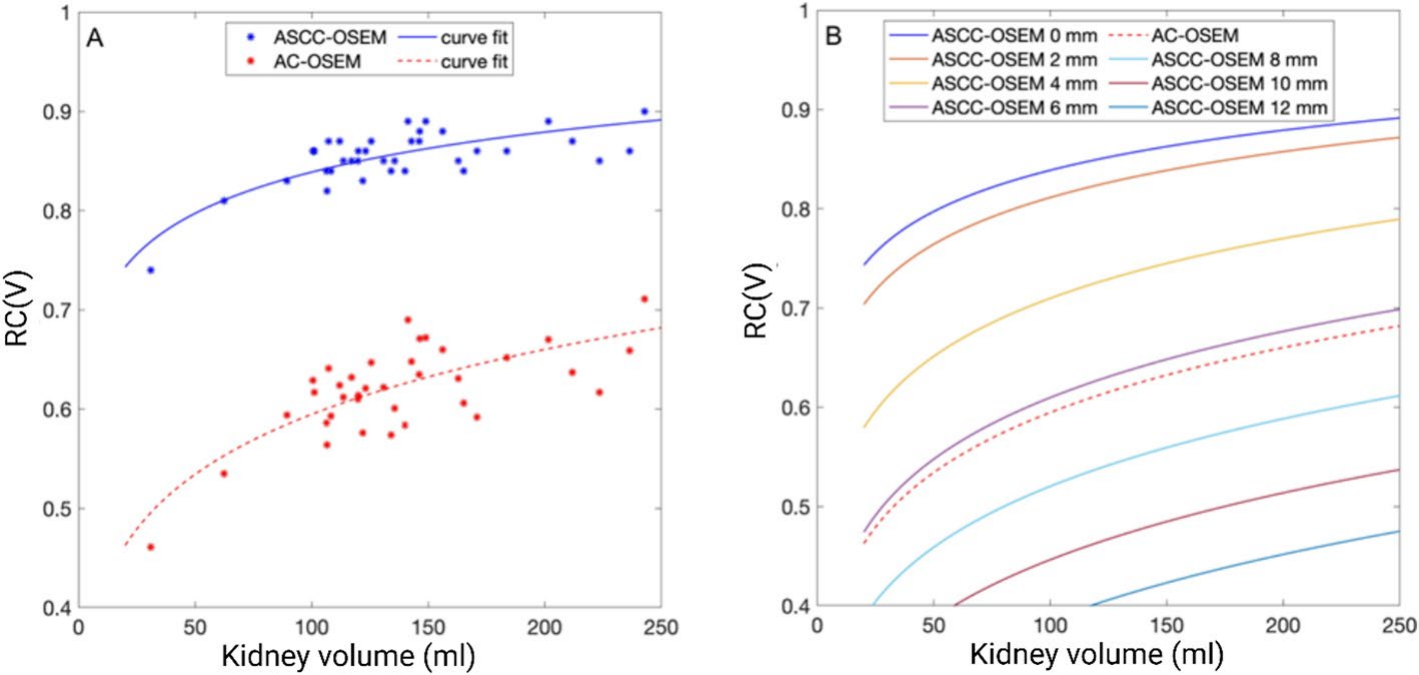
Recovery coefficients (RCs) for 36 manually delineated kidney volumes using different SPECT reconstruction methods. (**A**) ASCC-SPECT (blue dots and curve fit line) yielded higher RC values compared to AC-SPECT (red dots and curve fit line, reconstructed without Gaussian post-filter). (**B**) The curve fits of RC versus kidney volumes for AC-SPECT and ASCC-SPECT, with and without post-filtering. The ASCC-SPECT-based and AC-SPECT-based reconstructed images were post-processed with a Gaussian filter (0–12 mm) and a Hann filter (cut-off 0.85), respectively

For the 36 manually delineated kidneys on patient CT images, the volumes were between 31 and 243 mL. The corresponding RC had a mean (max-min) value of 0.85 (0.73–0.90) and 0.62(0.46–0.71) for ASCC-SPECT and AC-SPECT, respectively (Fig. 2A). The corresponding coefficient of variation values were 3.3% and 7.4%, respectively. In Fig. 2, the general trend of the RCs versus kidney volume is described using the non-linear equation as detailed in the Methods section. The fitting parameters are a = 552 and b = 0.0934.

Applying post-Gaussian filtering (GF) on ASCC-SPECT had a pronounced effect on RC (Fig. 2B). Using a 6-mm GF resulted in RC values similar to those achieved using AC-OSEM with a Hann filter with a cut-off of 0.85.

### Influence of the SV volume on kidney absorbed dose

The SVs were positioned in regions that were visually identified as representative of mean activity concentration areas, i.e., avoiding hotspots of high and low activity (Fig. 1). It was visually observed that the SV_4_ was often larger than the kidney parenchyma thickness, while SV_2_ closely matched the parenchyma thickness. SV_0.6_ was entirely within the kidney parenchyma. All data for the different segmentation methods are available as supplement (Table  S1-S54 supplemental data file).

**Fig. 3:**
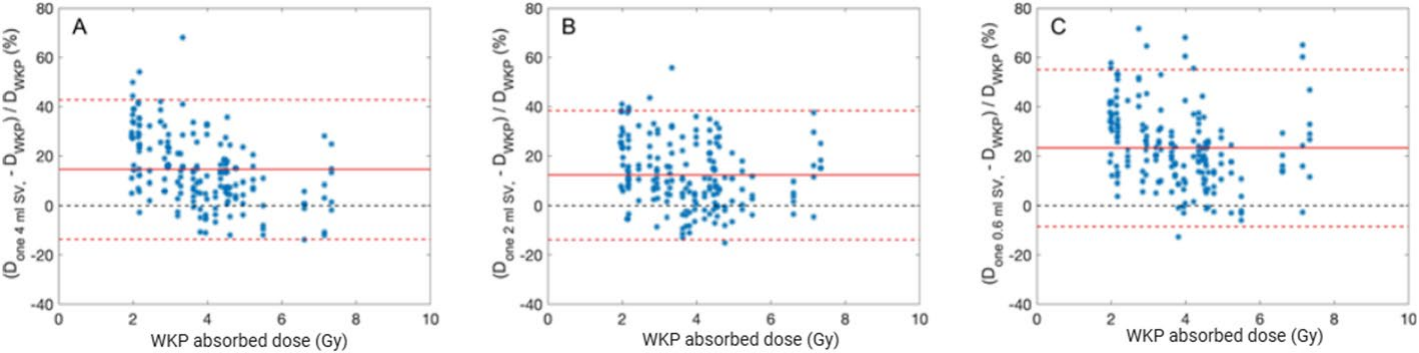
The Bland-Altman analysis of kidney absorbed doses estimated from the small volume of interest (SV) methods, with the whole-kidney parenchyma (WKP) as the reference method. No post-filtering was used. (**A**) One SV_4_. (**B**) One SV_2_. (**C**) One SV_0.6_. Solid red lines indicate the bias, and dashed red lines indicate the upper and lower 95% limits of agreement (calculated as bias ± 1.96 × SD)

The Pearson correlation coefficient against the WKP dose increased with the number of SVs used to estimate the mean absorbed dose. Correlation coefficient values of 0.92, 0.95, 0.97, 0.97, and 0.97, respectively, were obtained when including 1, 2, 3, 4, and 5 SV_4_ in the estimate of the mean kidney activity concentration. The corresponding correlation coefficients for SV_2_ ranged from 0.96 to 0.97. The bias and accuracy were lower for the SV_2_ method compared to the SV_4_ and SV_0.6_ methods (Fig. 3). The SV methods overestimated the kidney absorbed doses in non-post-filtered SPECT images. Applying post-filtering resulted in a decreased bias (Fig. S1– S2, supplemental data). Therefore, kidney absorbed doses calculated in post-filtered SPECT images were closer to the line of identity in a linear plot. However, applying excessive filtration resulted in decreased linear correlation to the line of identity (Fig. S1–S2).

### Accuracy and application of a normalization factor for the SV method with WKP as reference method

**Fig. 4:**
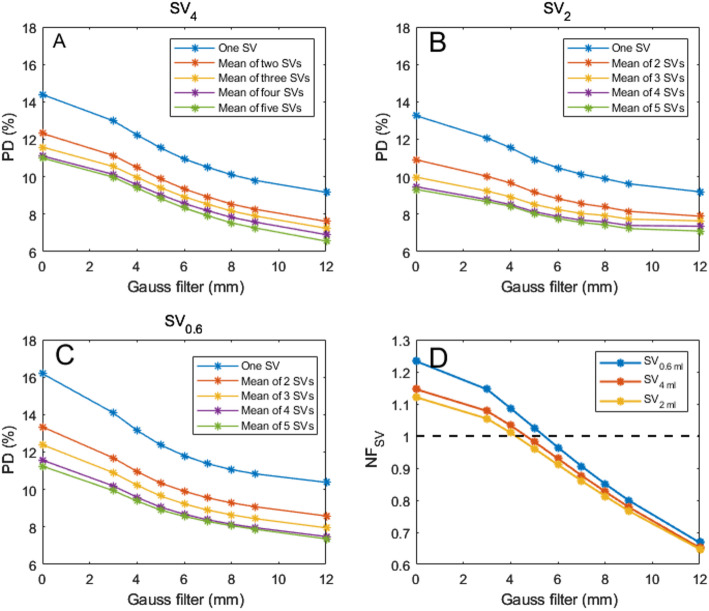
(**A**–**C**) Accuracy of the kidney absorbed doses with different numbers of small volumes of interest (SVs) in ASCC-SPECT post-filtered with a Gaussian filter of 0–12 mm. (**A**) SV_4_. (**B**) SV_2_. (**C**) SV_0.6_. (**D**) The apparent recovery coefficient (normalization factor) for the SVs, as determined from the ratio of the absorbed dose ratio between the SV and the whole-kidney parenchyma (WKP) method versus the sigma values used for post-filtering using a Gaussian filter. The dashed black line represents the line of identity

As shown in Fig. 4, the accuracy was dependent on the number of SVs and the SPECT resolution. With one SV_4_, the accuracy was about 14%, and decreased to about 11% with the mean of five SV_4_s in unfiltered SPECT images. The lowest accuracy was obtained with the smallest sphere size, i.e., SV_0.6_. The highest accuracy was obtained with the SV_2_. Using the mean of five SV_2_s yielded a accuracy of 9.4%. Increasing the number of SVs to above five had only minor impact on accuracy (data not shown). Notably, applying Gaussian post-filtering also improved accuracy. The bias, expressed as the normalization factor, were highest for SV_0.6_ and lowest for SV_2_ (Fig. 4D). Notably, a Gaussian filter with a sigma value of 5–6 mm resulted in recovery coefficients almost equal to unity. However, higher sigma values resulted in decreased recovery coefficients.

**Fig. 5:**
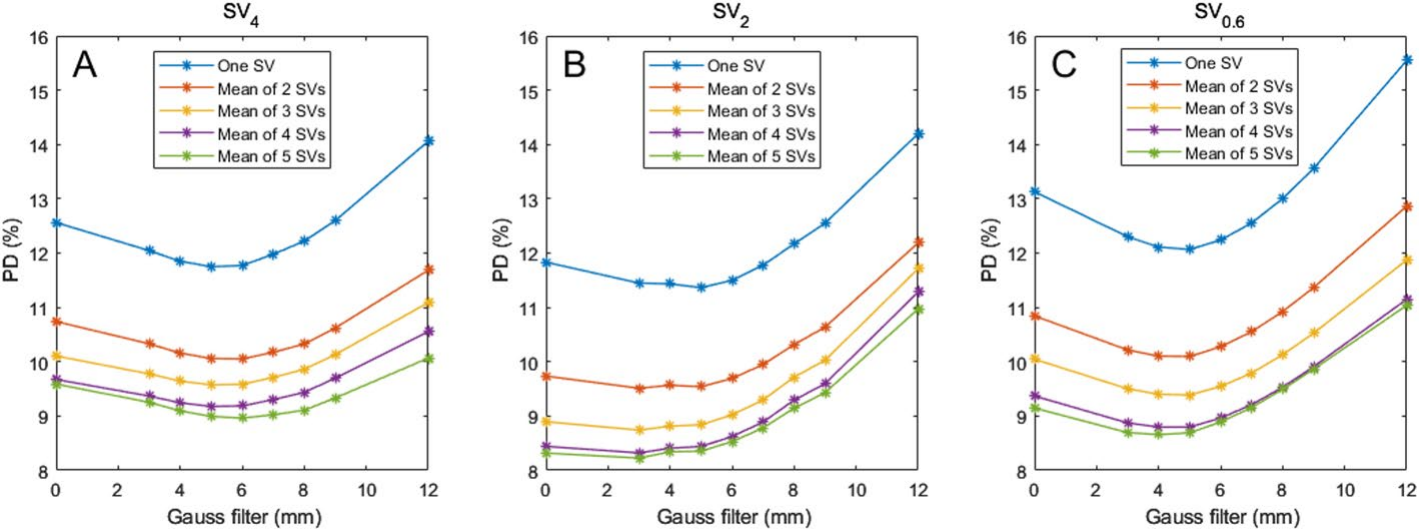
Accuracy for the normalized factor corrected kidney absorbed doses for different numbers of small volumes of interest (SVs) in ASCC-SPECT post-filtered with a Gaussian filter of 0–12 mm. (**A**) SV_4_. (**B**) SV_2_. (**C**) SV_0.6_

When the absorbed doses obtained using the SV methods were corrected with the NF, the accuracy were improved (Fig. 5). The highest accuracy was obtained for SV_2_. When using one SV_2_, and filtering with sigma values of 0–6 mm, the accuracy was between 11.5% and 11.8%. When using the mean of five SV_2_s, the corresponding values were 8.2% and 8.5%. Higher sigma values resulted in decreased accuracy due to the decreased correlation to the line of agreement between the SV and WKP method (Fig. S1-S2> Supplemental data). Table 1 summarizes the accuracy associated with the SVs method when using a 4 mm Gaussian filter.

**Table 1:** Accuracy for normalized factor corrected small volume of interests using a 4 mm Gaussian postfilter

Segmentation method	PD (%)				
	One SV	Two SVs	Three SVs	Four SVs	Five SVs
SV_4_	11.8	10.2	9.6	9.2	9.1
SV_2_	11.4	9.6	8.8	8.4	8.3
SV_0.6_	12.1	10.1	9.4	8.8	8.7

**Fig. 6:**
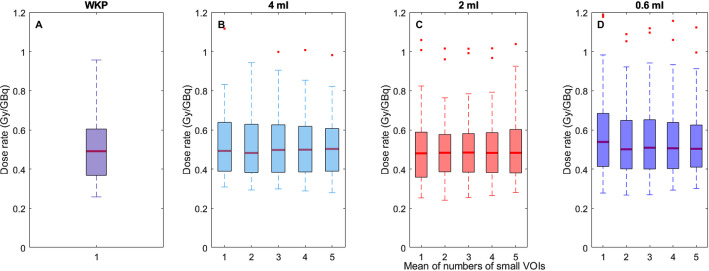
The kidney absorbed dose rate (Gy/GBq) distributions for the patient cohort when using the WKP method (**A**), the SV_4_ method (**B**), the SV_2_ method (**C**), and the SV_0.6_ method (**D**) in 4 mm Gaussian post-filtered SPECTs, respectively

The kidney absorbed dose rate distributions for the patient cohort using the different methods are presented in Fig. 6. The median absorbed dose rate is slightly higher for the WKP method than for the SV methods, although no other significant differences are observed between the dosimetry methods.

## Discussion

Our results demonstrate that the method using a single SV overestimated the kidney absorbed doses and is thus not optimal for kidney dosimetry with these imaging settings, calibrations and reconstructions. However, the SV methodology was easily improved by using multiple SVs per kidney parenchyma. Simply increasing the number of SVs from one to three increased the accuracy in the kidney absorbed doses to approximately 9%. Our findings support that an optimized and calibrated SV method including multiple SVs would be useful for estimating the mean kidney absorbed dose in the time-pressed clinical workflow [17, 18].

The reliability of the WKP method depends primarily on the accuracy in the WKP volume estimation. Manual segmentation of the WKP volume in CT images can be challenging, particularly when adjacent tissues have similar Hounsfield values. This introduces errors in the volume estimation, which subsequently affects the adopted RC value, as it is volume dependent. Additionally, spill-in from surrounding tissues with high uptake is not accounted for in the RC calculation. These two factors represent the primary sources of potential inaccuracy in the WKP method and would be valuable topics for further analysis in future studies.

For ASCC-SPECT, the mean value of the simulated RC was 0.85, with a range between 0.73 and 0.90, depending on volume and shape of the kidney parenchyma. This study reports the variability of RC values, and to apply kidney specific PVE corrections in kidney dosimetry. Notably, several studies have presented kidney RC values for ^177^Lu, and have used spherical phantoms for extrapolation of individual RCs [17, 18]. However, it has been demonstrated that the RC values for spherical phantoms do not account for the true shape of the kidney. Tran-Gia et al. [19] calculated an RC of 0.83 for a 100-mL sphere, while the RC was only 0.62 for a cortex phantom of 100 mL [19]. Moreover, Finocchioro et al. [20] found that the RC value for a 100-mL sphere was 0.82, while the RC was only 0.73 for a 142-mL kidney phantom. These previous results indicate that patient-specific RC values must be determined by simulations or in multiple printed parenchyma phantoms, as have been used for RC estimates of ^99m^Tc [20]. Our results demonstrate that the image resolution has a large impact on RC, addressing the importance of using a SPECT system and reconstruction-specific RCs. With AC-SPECT (or equivalent ASCC-SPECT with 6-mm GF), the mean RC was only 0.62, compared to an RC value of 0.85 with non-filtered ASCC-SPECT. This value of 0.62 is in good agreement with the phantom determined RC values of 0.61–0.64 for kidney volumes of 144–169 mL using ASCC-SPECT [21, 22]. Moreover, the RC accuracy was 3.3% and 7.4% for ASCC-SPECT and AC-SPECT, respectively, indicating the advantage of using ASCC-OSEM with a high number of updates and no post-filtering for estimating mean absorbed doses based on the WKP method. However, with the SV method, it seems to be a disadvantage to use too high image quality, since the highest accuracy were obtained with slightly filtered ASCC-OSEM. In the first publication with the single SV_4_ method, AC-SPECT with post-filtering was used; however, the accuracy were quite low: 24% and 15% for the right and left kidney, respectively. The results showed only a modest correlation with the WKP (*r* = 0.81 and *r* = 0.91) for right and the left kidneys, respectively, as determined from the data presented in the publication by Sandström et al. [7]. In our present study, with the equivalent reconstruction method, we obtained higher accuracy and a higher correlation (*r* = 0.92). This might be partly explained by the fact that in the present study, the SV_4_ was placed in a region representative of cortex/medulla of each kidney, toward the kidney pelvis, while Sandström et al. [8] located the SV_4_ in the highest uptake region. This interpretation is based on our hypothesis regarding methodological differences and should not be construed as a direct comparative analysis of the two approaches. When Heikkonen et al. [9] positioned the SV_4_ in the highest uptake region in ASCC-OSEM reconstructed SPECT, the mean absorbed dose was overestimated by 70% (33–123%). Additionally, Hou et al. [10] reported overestimated values by 80 ± 50% and 70 ± 40% for the right and left kidney, respectively. In our study, with SV_4_ located in a region representative of the mean activity, the corresponding bias and accuracy were 15 ± 14%. However, despite this improvement obtained by using a region representative of the mean activity concentration, it is not a well-defined guideline.

Our results indicates that more than one single SV should be used to obtain an estimated mean absorbed dose with increased accuracy. Consequently, we used the mean of up to five SVs, and found that this improved the overall accuracy from 12% to around 9%. Notably, the use of more than five SVs had minor influence on further improving the accuracy, and it is challenging to find further representative regions in small kidneys.

The accuracy could be further improved by using SVs smaller than the 4-mL SV (i.e., SV_2_). The rationale for using smaller SVs is that the 20 mm diameter of SV_4_ could exceed the parenchymal thickness for many kidneys; normal adults have a parenchymal thickness of 15–22 mm [12, 13]. The diameter of SV_2_ is 15 mm and should thus better match most kidneys, as was visually confirmed in this study. Ardenfors et al. [11] recently used SV_0.6_ in post-filtered ASCC-SPECT for kidney and tumor dosimetry, but they did not evaluate the accuracy or accuracy. Our present results demonstrated that the SV_0.6_ method might be an additional option if sufficient number of SV_0.6_ VOIs are positioned in representative uptake regions.

Hou et al. [10] and Heikonnen et al. [9] evaluated the SV method using ASCC-SPECT, with no RC correction applied, which resulted in mean absorbed dose estimates that were about 1.7-times higher for the SV_4_ method compared to the WKP method. No RC correction was used in the SV_0.6_ method. This makes it challenging to delve direct comparison without access to the actual images from published studies [9, 10, 11]. Furthermore, difference in methodology, such as the inclusion of a PVE correction in Sandström et al. (2010), the use of energy peaks at 113 keV and 208 keV without scatter correction for SPECT image reconstruction, and the use of large VOIs in the Heikkonen et al., study complicate the comparison. Sandström et al. [7] determined a calibration factor (CF) for AC-OSEM reconstructed SPECT, by using a water phantom with a centrally placed 100-mL spherical ^177^Lu source. The CF for SV_4_ was determined in the center of the source, and the CF for WKP was determined with a 120-mL VOI surrounding the source. As addressed above, a 100-mL sphere seems to poorly resemble the kidney parenchyma with regard to PVEs; therefore, it is advisable to use calibration methods other than spheres. In the present study, we applied NFs for the SV method based on the dosimetry ratio between the SVs methods and the reference method, i.e., the WKP doses as shown in Fig. 4D. However, this is a time-consuming methodology that hampers establishing a similar dosimetry protocol for new clinical radiopharmaceuticals.

In comparison with Sandström et al. (2015), who reported Pearson correlation coefficients ranging from 0.92 to 1.00 using a 4 mL SV VOI method, our study demonstrates similar strong correlations (0.96 to 0.97) with a 2 mL VOI. However, several factors complicate direct comparison between studies. For instance, Sandström et al. did not specify the correlation method used, and their study did not apply scatter correction. While the absence of scatter correction can affect quantitative accuracy—particulary in small volumes, where spill-in from scattered photons may be more pronounced—its specific impact on correlation strength remains unclear and warrants further investigation. In contrast, our study applied appropriate scatter correction methods, which improve quantification accuracy by mitigating spill-in effects. Additionally, the use of a smaller VOI size in our analysis may reduce partial volume effects (PVEs), which can otherwise bias measurements by including non-target areas. This reduction in PVEs may explain the slight differences in correlation strengths observed between the two studies. Despite these methodological differences, our findings support the clinical applicability of the 2 mL VOI approach, particularly in settings where the patient kidney parenchymal rim is thin.

In our study, the focus was on optimizing the SV method by integration modern correction technquies, including scatter and partial volume corrections, with AC-OSEM reconstructions employing resolution recovery. The application of Gaussian post-filtering mitigates potential Gibbs artifacts, thereby enhancing the reliability of quantifications. In contrast, the study by Curkic Kapidzic et al. [23] demonstrated the stability and accuracy of small VOI quantification using reconstructions without resolution recovery and system-specific RCs. While both approaches have their merits, direct comparisons of these methods across various reconstruction techniques are needed to fully understand the trade-offs between operator independence, the impact of Gibbs artifacts, and quantification accuracy. Such comparisons would help establish best practices for kidney dosimetry, tailored to clinical settings and available reconstruction tools.

Limitations of this study include that no proven true value is available, and the RCs were obtained from simulated data assuming uniform activity distribution in the kidney parenchyma, which does not account for possible non-uniform impacts on the RCs. Further studies must be performed to investigate how this factor might influence the RC values when accurate distribution data are available. In addition, the presence of high activity uptake in background due to liver or tumors, can influence determination of true RCs value, leading to underestimation or overestimation of the true activity concentrations in the WKP based on limited resolution of SPECTs. This background effect might also affect the SVs and needs to be further evaluated.

The higher value for the SV_0.6_ can be attributed to a PVE, which are amplified in SV due to the mismatch between the VOI size and the spatial resolution of SPECT images. For SV_0.6_ the counts are concentrated in fewer voxels, leading to an overestimation of the NFs. This is a common limitation in nuclear medicine imaging, where resolution effects can inflate the apparent signal for small objects. In contrast the 4 mL VOI, being larger than the kidney cortex rim, may include surrounding background activity, diluting the measured signal and resulting in lower NFs values. The 2 mL VOI more closely matches the typical width of the kidney cortex, minimizing both PVEs and background inclusion, making it the most accurate representation of the true activity distribution in this case.

Both the WKP and SV methods require that accurate estimates of the RCs are performed. In the recently published EANM guidelines a RC value of 0.85 is recommended for the WKP method [24]. However, it is characterized in this study that the RC is dependent on the volume and shape of the WKP as well as the reconstruction method. Therefore, it would be preferable to perform RC determination using printed kidney phantoms with a fillable parenchymal compartment of different sizes. Alternatively, one can use Monte Carlo simulation methods to determine individual RCs, however, for clinical use this would require a validated Monte Carlo method.

The drawback of the WKP method is that it is time-consuming, and the manually CT-delineated WKP may require adjustment to align with the SPECT image of the kidney. Furthermore, it is operator-dependent, which could result in slight variations in the determined volumes. The extent of these variations should be further investigated, and it would be beneficial for multiple research group to explore this in future studies. In contrast, SVs can be rapidly inserted into the SPECT volume of the kidney parenchyma. With our results in mind, we recommend using at least three SVs for determining the mean absorbed dose. The size of the SVs should be 2 mL or less for matching the size of the parenchyma width. Further, it is recommended to limit the number of updates with ASCC-OSEM reconstructed SPECT protocols since a high number of updates will increase the non-uniformity within the kidney parenchyma which will make it challenging to find representative SV regions of the mean kidney activity concentration. Consequently, post-filtering of high-resolution SPECT images is recommended. In our study, a Gaussian filter with a kernel size of approximately 2–6 mm produced the highest accuracy (Fig. 5). For the WKP method the opposite is to recommend, since increasing number of updates will decrease the spill out of counts and the RC will approach one, i.e., the accuracy in PVE correction will decrease.

The SV method was developed for decreasing the time-consuming manually delineation of kidneys. However, the rapid development of AI based segmentation tools might overcome this issue [16, 25]. Several vendors have already implemented AI based segmentation. These methods need validation of its accuracy and accuracy in the estimate of absorbed doses, estimate of time it will take to correct misplaced segmentations. Our experience is that AI based methods often fail to segment kidneys when cysts are present, which often is the case in elderly patients [26]. Nevertheless, AI based segmentation have potential to speed up the dosimetry workflow and become as fast as the application of SVs.

## Conclusions

This study demonstrates that the bias of mean absorbed dose for the SV method, compared to the reference method using PVE-corrected activity concentration estimates in manually segmented WKP, depends on both image quality and SV volume. In unfiltered images corrected for attenuation, scatter, and collimator detector response, the SV method overestimated the difference in agreement by up to 22%. However, applying a Gaussian filter with a kernel size of approximately 5 mm produced absorbed dose estimates comparable to the reference WKP method, highlighting the importance of appropriate filtering for accurate dose estimation.

Analysis of the original SV method, which used a single 4 mL SV, revealed its suboptimal performance for kidney dosimetry. Improved accuracy with the reference method were achieved by resizing the SV to better match the kidney parenchyma and strategically placing multiple SVs at representative locations within the kidney parenchyma in post-filtered SPECT images. The protocol that achieved the highest accuracy involved five 2 mL SVs, improving the accuracy to 8.3% compared to the WKP method.

## Electronic Supplementary Material

Below is the link to the electronic supplementary material.

Supplementary Material 1

Supplementary Material 2

## Data Availability

The data that support the findings of this study are available from the corresponding author upon reasonable request.
