# Supplementary material for: Evaluation of using small volume of interest regions for clinical kidney dosimetry in 177Lu-DOTATATE treatments
Source: EJNMMI Phys. 2025 Jul 8;12:66. doi: 10.1186/s40658-025-00769-w (PMC12234931; doi:10.1186/s40658-025-00769-w)
Supplement: Supplementary file 1 — Supplementary Material 1 [file 40658_2025_769_MOESM1_ESM.docx]

**Supplement**

**Evaluation of Using Small Volume of Interest Regions for Clinical Kidney Dosimetry in ^177^Lu-DOTATATE Treatments**

Jehangir Khan^1,2,5^, Tobias Rydèn^1^, Martijn Van Essen^3^, Johanna Svensson^4^, and Peter Bernhardt^1,5^

^1^Department of Medical Physics and Biomedical Engineering (MFT), Sahlgrenska University Hospital, Gothenburg, Sweden, ^2^Department of Medical Physics, Faculty of Medicine and Health, Örebro University Hospital, Örebro, Sweden, ^3^Department of Clinical Physiology, Sahlgrenska University Hospital, Gothenburg, Sweden, ^4^Department of Oncology, Institution of Clinical Sciences, Sahlgrenska Academy at University of Gothenburg, Sweden, ^5^Department of Medical Radiation Sciences, Institute of Clinical Sciences, Sahlgrenska Academy at University of Gothenburg, Sweden.

**Corresponding author:**

Jehangir Khan, Department of Medical Physics and Biomedical Engineering (MFT)

Sahlgrenska University Hospital, Gothenburg, SE-41345 Sweden; E-mail:

[Jehangir.khan@gu.se](mailto:Jehangir.khan@gu.se); Phone: +46- (0) – 736772531

**Running title:** Small VOI in ^177^Lu-DOTATATE dosimetry

**Number of figures and tables:** 3

**Key Words:** ^177^Lu-DOTATATE; neuroendocrine; single-photon emission tomography; SPECT/CT; kidney dosimetry; recovery coefficient


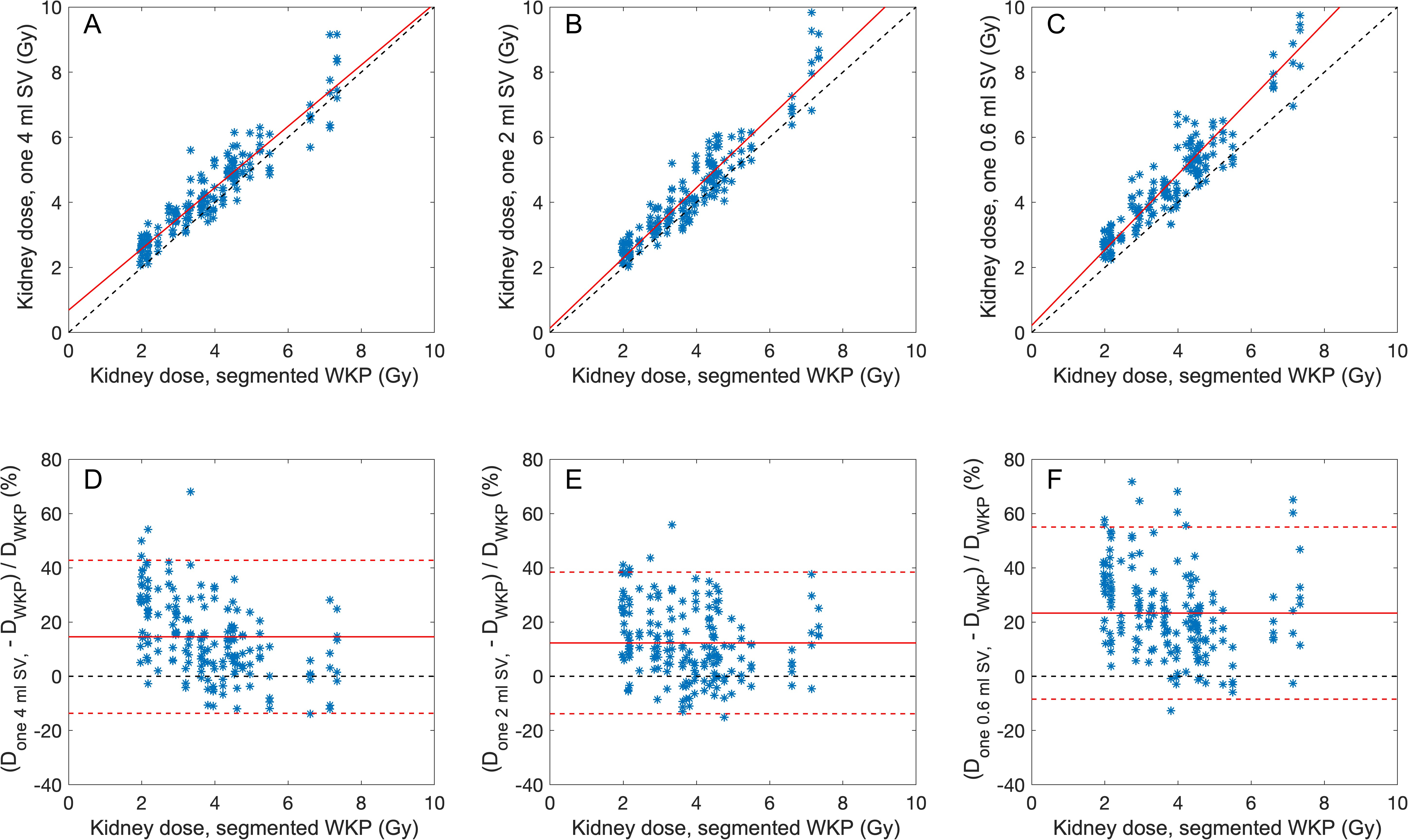


**FIGURE S1.** Bland-Altman and Pearson’s correlation plots of the kidney absorbed doses estimated from the whole-kidney parenchyma (WKP) and small volume of interest (SV) methods based on unfiltered ASCC-OSEM reconstructed images. (A–C) Pearson’s correlation plots of the kidney absorbed doses estimated using one SV_4_ (A), one SV_2_ (B), and one SV_0.6_ (C). (D–F) Bland-Altman plots of one SV_4_ (D), one SV_2_ (E), and one SV_0.6_ (F). Solid red lines indicate the best fit (A–C) and the mean of the differences (bias) value (D–F) between two methods. Dashed red lines indicate the upper and lower 95% limits of agreement (bias ± 1.96 × SD). The dashed black lines indicate the line of identity.


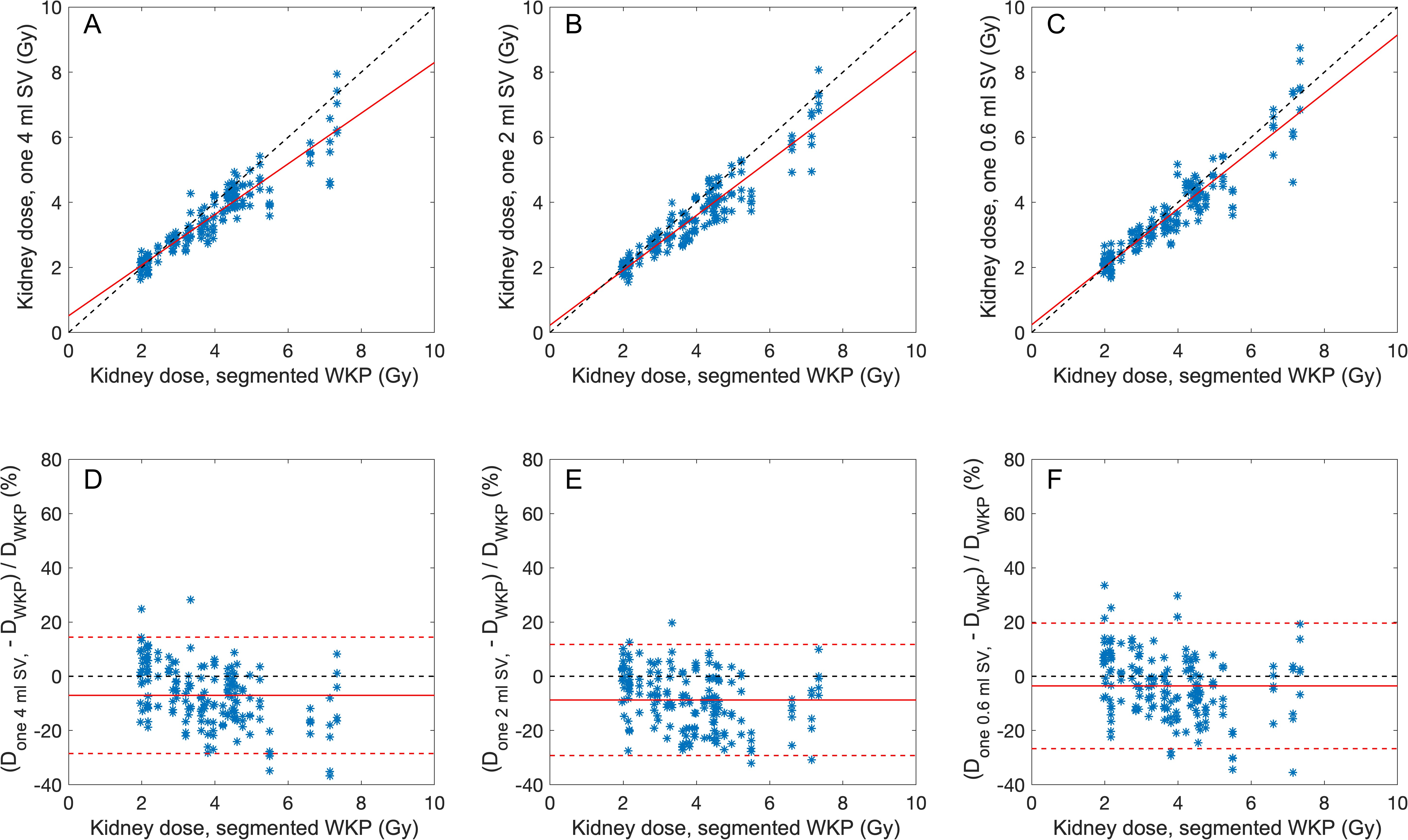


**FIGURE S2.** Bland-Altman and Pearson’s correlation plots of kidney absorbed doses estimated from the whole-kidney parenchyma (WKP) and small volume of interest (SV) methods in ASCC-OSEM reconstructed SPECTs and postfiltering by Gaussian filter (6 mm). (A–C) Pearson’s correlation plots of the kidney absorbed doses estimated using one SV_4_ (A), one SV_2_ (B), and one SV_0.6_ (C). (D–F) Bland-Altman plots of one SV_4_ (D), one SV_2_ (E), and one SV_0.6_ (F). Solid red lines indicate the best fit (A–C) and the mean of the differences (bias) value (D–F) between two methods. Dashed red lines indicate the upper and lower 95% limits of agreement (bias ± 1.96 × SD). The dashed black lines indicate the line of identity.

The activity concentrations for each patient determined using WKP and the small VOI (0.6, 2, and 4 mL) methods as shown in tabular form (Table S1-S54) are available in attached supplement data file.

Where, abbreviations WKP represented whole kidney parenchyma volume; VOI (volume of interest); sigma (Gaussian post-filtered with sigma value (0-12 mm); Time p.i. (time post injection; inj Act (injected activity); RC (recovery coefficients).
