# Supplementary material for: Evaluation of using small volume of interest regions for clinical kidney dosimetry in 177Lu-DOTATATE treatments
Source: EJNMMI Phys. 2025 Jul 8;12:66. doi: 10.1186/s40658-025-00769-w (PMC12234931; doi:10.1186/s40658-025-00769-w)
Supplement: Supplementary file 2 — Supplementary Material 2 [file 40658_2025_769_MOESM2_ESM.pdf]

Table S1 shows the activity concentrations measured using WKP and with 0.6 mL small VOI methods on SPECTs images post-filtered with Gaussian filter (0-12 mm) for patient number 1. Whereas, RC represented patient specific recovery coefficient of right and the left kidneys, respectively.

**Post-filtering (mm)**	**Data**	**Time p.i. (h)**	**Inj Act (MBq)**	**Right Kidney**							**Left Kidney**						
			7672	**VOI_1 (counts/mL)**	**VOI_2 (counts/mL)**	**VOI_3 (counts/mL)**	**VOI_4 (counts/mL)**	**VOI_5 (counts/mL)**	**WKP (counts/mL)**	**RC**	**VOI_1 (counts/mL)**	**VOI_2 (counts/mL)**	**VOI_3 (counts/mL)**	**VOI_4 (counts/mL)**	**VOI_5 (counts/mL)**	**WKP (counts/mL)**	**RC**
sigma = 0	T1_1	21,0		844,69	822,29	992,28	892,04	896,09	643,51	0,89	992,24	1039,52	1135,79	864,67	882,84	593,48	0,86
	T2_1	48,8		601,84	596,92	630,83	591,28	628,55	447,5		560,17	501,07	573,32	703,58	648,56	405,35	
	T7_1	170,4		106,72	133,93	140,85	119,69	126,26	76,42		171,91	142,09	114,04	124,86	105,5	65,31	
Sigma = 3	T1_1			778,8	772,93	930,49	851,24	851,76	643,51		883,27	961,26	1041,31	795,09	812,67	593,48	
	T2_1			547,24	566,55	602,91	568,7	578,32	447,5		518,86	486,42	513,98	638,48	586,36	405,35	
	T7_1			98,65	119,66	123,18	107,72	114,05	76,42		148,39	132,9	107,99	112,8	94,96	65,31	
sigma = 4	T1_1			737,97	738,19	886,58	823,01	817,42	643,51		817,31	910,72	978,21	752,23	766,44	593,48	
	T2_1			513,84	544,23	583,27	549,82	545,31	447,5		490,17	474,77	479,25	599,04	546,86	405,35	
	T7_1			93,3	111,87	113,7	100,68	106,01	76,42		135,64	126,15	103,95	106,46	88,97	65,31	
sigma = 5	T1_1			694,65	700,98	837,81	790,39	777,3	643,51		750,42	855,62	909,1	706,98	716,17	593,48	
	T2_1			479	519,06	560,64	525,6	508,8	447,5		458,67	459,06	444,78	557,91	504,89	405,35	
	T7_1			87,53	104,49	104,95	93,71	97,74	76,42		123,67	118,28	99,13	100,35	83,18	65,31	
sigma = 6	T1_1			650,54	664,44	788,15	754,54	734,49	643,51		686,29	798,86	838,25	661,43	664,73	593,48	
	T2_1			444,45	493,32	535,97	498,06	471,3	447,5		426,77	439,89	412,1	517,31	463,05	81,74	
	T7_1			81,74	97,74	97,24	87,17	89,95	76,42		112,79	109,79	93,64	94,4	77,79	65,31	
Sigma = 7	T1_1			607,37	630,44	740,39	716,98	691,26	643,51		627,22	743,04	769,18	617,26	614,67	593,48	
	T2_1			411,66	468,75	510,6	469,15	434,77	447,5		396,22	418,21	381,93	478,85	423,24	405,35	
	T7_1			76,21	91,72	90,64	81,23	82,94	76,42		103,03	101,16	87,79	88,55	72,81	65,31	
Sigma = 8	T1_1			566,39	599,78	695,93	679,05	648,95	643,51		574,07	689,58	704,14	575,5	567,57	593,48	
	T2_1			381,5	446,4	485,68	440,32	400,39	447,5		367,94	395,16	354,4	443,21	386,48	405,35	
	T7_1			71,1	86,42	85	75,9	76,7	76,42		94,29	92,81	81,9	82,78	68,18	65,31	
Sigma = 9	T1_1			528,36	572,47	655,24	641,75	608,29	643,51		526,84	639,04	644,35	536,58	524,24	593,48	
	T2_1			354,37	426,59	461,9	412,49	368,71	447,5		342,28	371,8	329,43	410,45	353,22	405,35	
	T7_1			66,5	81,81	80,12	71,12	71,14	76,42		86,42	85,03	76,2	77,13	63,38	65,31	
Sigma = 12	T1_1			434,37	505,55	554,29	538,77	498,82	643,51		415,63	506,59	499,44	436,61	417,04	593,48	
	T2_1			290,82	379,11	399,22	338,47	290,54	447,5		280,05	307,22	268,29	327,47	273,5	405,35	
	T7_1			55,79	71,13	68,47	59,32	57,4	76,42		67,32	66,48	61,27	61,65	52,22	65,31	

Table S2 shows the activity concentrations measured using WKP and with 0.6 mL small VOI methods on SPECTs images post-filtered with Gaussian filter (0-12 mm) for patient number 2. Whereas, RC represented patient specific recovery coefficient of right and the left kidneys, respectively.

Post-filtering (mm)	Data	Time p.i. (h)	Inj Act (MBq)	Right Kidney							Left Kidney						
			7692	VOI_1 (counts/mL)	VOI_2 (counts/mL)	VOI_3 (counts/mL)	VOI_4 (counts/mL)	VOI_5 (counts/mL)	WKP (counts/mL)	RC	VOI_1 (counts/mL)	VOI_2 (counts/mL)	VOI_3 (counts/mL)	VOI_4 (counts/mL)	VOI_5 (counts/mL)	WKP (counts/mL)	RC
sigma = 0	T1_1	22,8		874,14	832,06	981,44	998,19	916,89	637,65	0,89	959,78	1076,46	1047,93	1009,66	797,97	661,07	0,89
	T2_1	50,4		580,51	627,15	682,31	764,64	763,93	545,18		719,55	696,95	873,81	722,08	748,83	523,75	
	T7_1	171,3		136,33	133,5	156,86	135,43	132,55	103,62		131,69	112,11	154,08	122,57	131,15	105,79	
Sigma = 3	T1_1			825,74	770,46	918,03	947,7	860,33	637,65		900,72	1010,22	992,39	923,06	757,28	661,07	
	T2_1			540,58	582,8	642,9	707,67	693,67	545,18		669,28	657,59	804,29	662,91	687,43	523,75	
	T7_1			125,04	120,9	141,61	129,89	122	103,62		120,76	105,55	135,41	116,43	127,4	105,79	
sigma = 4	T1_1			789,64	737,95	876,37	907,56	824,74	637,65		858,4	965,28	953,06	872,31	730,59	661,07	
	T2_1			514,38	554,93	616,5	670,75	651,38	545,18		633,29	631,26	759,19	629,38	649,77	523,75	
	T7_1			117,21	114,51	133,18	125,43	115,71	103,62		114,88	101,4	125,09	112,61	123,07	105,79	
sigma = 5	T1_1			747,82	708,01	831,23	860,18	784,93	637,65		809,98	915,34	908,78	820,21	700,77	661,07	
	T2_1			485,87	526	587,66	631,1	607,69	545,18		592,01	601,49	711,85	596,21	610,03	523,75	
	T7_1			108,81	108,72	124,82	119,75	109,21	103,62		109,1	97,09	115,35	108,3	117,12	105,79	
sigma = 6	T1_1			769,12	826,25	918,42	979,47	874,05	637,65		920,94	877,38	1026,15	1057,59	998,16	661,07	
	T2_1			456,65	498,39	558,09	591,29	564,43	545,18		548,41	569,45	665,28	564,26	569,82	523,75	
	T7_1			100,52	103,5	116,94	113,38	102,73	103,62		103,41	92,9	106,65	103,61	110,61	105,79	
Sigma = 7	T1_1			738,76	799,11	882,23	925,42	825,55	637,65		865,34	838,26	969,31	1005,96	935,06	661,07	
	T2_1			427,99	473,37	529,08	553,11	522,95	545,18		505,12	536,59	620,96	533,79	530,51	523,75	
	T7_1			92,72	98,75	109,76	106,79	96,41	103,62		97,85	88,92	98,99	98,65	102,76	105,79	
Sigma = 8	T1_1			706,26	771,44	843,93	871,92	777,32	637,65		810,92	800,76	913,08	953,91	874,19	661,07	
	T2_1			400,72	451,15	501,32	517,4	483,99	545,18		463,96	504,19	579,39	504,73	492,93	523,75	
	T7_1			85,58	94,3	103,27	100,3	90,33	103,62		92,47	85,14	92,22	93,57	95,34	105,79	
Sigma = 9	T1_1			672,84	743,31	804,81	820,5	730,65	637,65		759,03	764,98	858,9	902,48	816,41	661,07	
	T2_1			375,26	431,31	475,05	484,36	447,83	545,18		425,94	473,24	540,73	476,96	457,55	523,75	
	T7_1			79,14	90,01	97,4	94,1	84,56	103,62		87,29	81,52	86,15	88,48	88,18	105,79	
Sigma = 12	T1_1			575,31	656,55	690,98	684,48	605,34	637,65		623,9	666,3	714,37	757,4	664,28	661,07	
	T2_1			310,51	379,59	405,19	399,76	355,89	545,18		333,09	393,24	441,43	400,77	365,97	523,75	
	T7_1			63,7	77,49	82,42	77,85	69,44	103,62		73,38	71,42	71,09	73,92	69,35	105,79	

Table S3 shows the activity concentrations measured using WKP and with 0.6 mL small VOI methods on SPECTs images post-filtered with Gaussian filter (0-12 mm) for patient number 3. Whereas, RC represented patient specific recovery coefficient of right and the left kidneys, respectively.

Post-filtering (mm)	Data	Time p.i. (h)	Inj Act (MBq)	Right Kidney							Left Kidney						
			7670	VOI_1 (counts/mL)	VOI_2 (counts/mL)	VOI_3 (counts/mL)	VOI_4 (counts/mL)	VOI_5 (counts/mL)	WKP (counts/mL)	RC	VOI_1 (counts/mL)	VOI_2 (counts/mL)	VOI_3 (counts/mL)	VOI_4 (counts/mL)	VOI_5 (counts/mL)	WKP (counts/mL)	RC
sigma = 0	T1_1	23,0		1377,13	1860,13	1660,34	1235,52	1374,27	903,77	0,81	1731,44	1814,87	1974,86	1748,51	1697,21	1233,85	0,90
	T2_1	50,2		1057,09	1325,75	1316,63	1074,11	848,36	683,92		1243,42	1229	1257,67	1394,64	1076,64	864,09	
	T7_1	169,5		255,15	435,57	452,28	297,87	262,26	207,99		249,63	288	312,81	317,51	245,92	193,94	
Sigma = 3	T1_1			1249,62	1633,67	1460,08	1120,22	1210,49	903,77		1642,69	1692,85	1873,31	1666,09	1597,29	1233,85	
	T2_1			953,19	1156,93	1147,56	959,09	753,52	683,92		1148,19	1144,27	1206,74	1329,76	1023,45	864,09	
	T7_1			237,1	377,51	378,23	261,62	227,07	207,99		248,89	265,33	293,82	286,03	241,2	193,94	
sigma = 4	T1_1			1171,63	1497,33	1340,45	1046,14	1119,64	903,77		1580,52	1614,75	1800,33	1615,84	1522,07	1233,85	
	T2_1			891,03	1060,87	1048,11	884,65	694,72	683,92		1088,56	1092,23	1168,83	1281,19	985,37	864,09	
	T7_1			225,84	342,11	338,14	240,68	206,62	207,99		245,68	252	283,3	269,31	233,52	193,94	
sigma = 5	T1_1			1091,96	1359,88	1222,7	969,41	1032,05	903,77		1508,72	1533,65	1717,59	1560,5	1435,02	1233,85	
	T2_1			826,96	966,07	949,65	808,58	634,46	683,92		1025,76	1036,54	1125,14	1224,35	940,05	864,09	
	T7_1			214,18	306,62	300,68	220,27	186,58	207,99		239,85	239,11	272,08	253,39	222,64	193,94	
sigma = 6	T1_1			1014,75	1228,9	1111,94	894,76	951,53	903,77		1430,58	1454,68	1630,73	1500,65	1343,19	1233,85	
	T2_1			766,11	876,32	857,41	736,37	576,29	683,92		962,97	979,26	1078,81	1162,89	889,46	864,09	
	T7_1			202,84	273,47	267,02	201,18	167,96	207,99		231,8	227,36	260,28	238,61	209,91	193,94	
Sigma = 7	T1_1			942,99	1109,33	1011,1	824,86	879,58	903,77		1349,69	1380,84	1544,05	1437,73	1252,19	1233,85	
	T2_1			710,59	794,35	774,31	670,74	522,5	683,92		902,5	922,7	1032,05	1099,98	836,18	864,09	
	T7_1			192,2	243,98	237,59	183,82	151,22	207,99		222,27	217	248,27	225,01	196,52	193,94	
Sigma = 8	T1_1			876,52	1003,09	920,82	760,44	815,83	903,77		1269,06	1313,3	1460,22	1373,3	1165,44	1233,85	
	T2_1			660,85	721,25	701,03	612,22	474,05	683,92		845,62	868,42	986,13	1037,93	782,53	864,09	
	T7_1			182,33	218,54	212,26	168,19	136,44	207,99		211,96	207,99	236,47	212,45	183,27	193,94	
Sigma = 9	T1_1			816,65	910,15	840,61	701,36	759,13	903,77		1190,91	1252,12	1380,68	1308,83	1084,64	1233,85	
	T2_1			616,49	657,06	637,01	560,31	431,07	683,92		792,91	817,29	941,78	978,32	730,3	864,09	
	T7_1			173,2	197	190,62	154,14	123,51	207,99		201,48	200,17	225,17	200,78	170,62	193,94	
Sigma = 12	T1_1			669,56	698,8	650,26	552,54	619,74	903,77		982,48	1099,79	1173,15	1125,85	880,6	1233,85	
	T2_1			508,53	510,59	489,53	436,2	330,78	683,92		659,94	685,32	821,97	821,41	591,32	864,09	
	T7_1			149,12	151,02	142,86	119,85	93,88	207,99		172,1	181,77	195,51	170,15	137,86	193,94	

Table S4 shows the activity concentrations measured using WKP and with 0.6 mL small VOI methods on SPECTs images post-filtered with Gaussian filter (0-12 mm) for patient number 4. Whereas, RC represented patient specific recovery coefficient of right and the left kidneys, respectively.

Post-filtering (mm)	Data	Time p.i. (h)	Inj Act (MBq)	Right Kidney							Left Kidney						
			7777	VOI_1 (counts/mL)	VOI_2 (counts/mL)	VOI_3 (counts/mL)	VOI_4 (counts/mL)	VOI_5 (counts/mL)	WKP (counts/mL)	RC	VOI_1 (counts/mL)	VOI_2 (counts/mL)	VOI_3 (counts/mL)	VOI_4 (counts/mL)	VOI_5 (counts/mL)	WKP (counts/mL)	RC
sigma = 0	T1_1	23,3		1159,17	1417,3	1345,67	1284,01	1015	879,22	0,87	1073,57	1118,65	1450,45	1214,82	1184,72	829,16	0,86
	T2_1	51,1		767,51	906,57	852,18	522,43	555,67	479,36		678,18	723,26	846,92	733,68	670,97	528,34	
	T9_1	217,8		71,81	94,4	110,21	75,39	66,33	46,72		65,83	80,28	73,28	75,73	90,41	47,8	
Sigma = 3	T1_1			1062,22	1317,08	1230,31	1189,53	925,3	879,22		986,03	1045,75	1310,99	1120,06	1100,69	829,16	
	T2_1			729,71	834,84	787,35	485,47	540,97	479,36		659,18	688,85	779,96	687,59	632,39	528,34	
	T7_1			69,22	86,31	92,04	70,49	60,73	46,72		59,54	71,26	65,83	67,51	79,52	47,8	
sigma = 4	T1_1			1007,69	1248,18	1160,36	1125,65	874,69	879,22		933,06	997,89	1229,25	1063,11	1040,53	829,16	
	T2_1			703,6	791,21	746,08	463,24	529,35	479,36		640,39	663,98	740,82	655,58	604,84	528,34	
	T9_1			67,11	81,09	82,73	67,07	57,33	46,72		55,86	66,04	61,62	63,21	73,5	47,8	
sigma = 5	T1_1			953,7	1172,75	1089,13	1055,78	824,08	879,22		878,17	947,12	1145,76	1003,81	972,63	829,16	
	T2_1			674,6	746,01	701,68	440,28	514,92	479,36		615,19	636,11	699,98	620,3	572,76	528,34	
	T9_1			64,53	75,58	74,5	63,27	53,69	46,72		52,23	60,92	57,52	59,11	67,4	47,8	
sigma = 6	T1_1			902,56	1096,69	1020	984,02	774,13	879,22		823,79	896,57	1064,41	944,8	902,59	829,16	
	T2_1			644,55	701,32	656,6	417,49	498,01	479,36		585,45	607,3	658,49	584,24	537,99	528,34	
	T9_1			61,68	70,09	67,46	59,24	49,95	46,72		48,88	56,17	53,65	55,19	61,46	47,8	
Sigma = 7	T1_1			855,67	1023,58	954,92	913,81	725,56	879,22		771,81	848,66	988,04	888,2	834,14	829,16	
	T2_1			614,81	658,44	612,88	395,4	479,14	479,36		553,44	579,22	617,75	549,06	502,23	528,34	
	T9_1			58,73	64,87	61,48	55,14	46,23	46,72		45,98	51,93	50,11	51,47	55,89	47,8	
Sigma = 8	T1_1			813,46	955,38	894,57	847,29	678,82	879,22		723,3	804,82	917,93	834,95	769,26	829,16	
	T2_1			586,02	617,98	571,7	374,2	458,92	479,36		521,13	552,9	578,81	515,7	466,84	528,34	
	T7_1			55,82	60,05	56,34	51,1	42,46	46,72		43,63	48,42	46,92	47,94	50,8	47,8	
Sigma = 9	T1_1			775,68	892,94	838,93	785,48	634,27	879,22		678,72	765,73	854,37	785,21	708,88	829,16	
	T2_1			558,36	580,16	533,57	353,95	437,92	479,36		489,87	528,85	542,37	484,52	432,82	528,34	
	T7_1			53,03	55,7	51,87	47,22	39,25	46,72		41,89	45,12	44,07	44,6	46,21	47,8	
Sigma = 12	T1_1			681,23	739,48	696,96	629,18	516,49	879,22		568,29	674,94	699,71	654,04	556,38	829,16	
	T2_1			481,14	482,45	437,6	298,67	374,72	479,36		408,09	469,46	450,11	403,48	343,94	528,34	
	T7_1			45,74	45,26	41,28	37,11	30,64	46,72		40,34	39,01	37,03	35,87	35,3	47,8	

Table S5 shows the activity concentrations measured using WKP and with 0.6 mL small VOI methods on SPECTs images post-filtered with Gaussian filter (0-12 mm) for patient number 5. Whereas, RC represented patient specific recovery coefficient of right and the left kidneys, respectively.

Post-filtering (mm)	Data	Time p.i. (h)	Inj Act (MBq)	Right Kidney							Left Kidney						
			7726	VOI_1 (counts/mL)	VOI_2 (counts/mL)	VOI_3 (counts/mL)	VOI_4 (counts/mL)	VOI_5 (counts/mL)	WKP (counts/mL)	RC	VOI_1 (counts/mL)	VOI_2 (counts/mL)	VOI_3 (counts/mL)	VOI_4 (counts/mL)	VOI_5 (counts/mL)	WKP (counts/mL)	RC
sigma = 0	T1_1	19,9		1077,78	1062,66	1461,57	1338,3	1278,14	1011,9	0,87	962,01	1125,14	829,98	928,39	1323,51	708,57	0,74
	T2_1	43,8		1065,82	1063,9	1225,38	1126,35	1092,62	846,1		765,66	791,88	841,54	828,21	793,55	599,16	
	T7_1	169,2		207,56	244,41	230,07	190,34	191,75	140,46		151,91	115,32	175,41	146,67	174,25	98,76	
Sigma = 3	T1_1			1056,7	1044,47	1366,62	1259,18	1205,62	1011,9		874,76	1010,58	772,01	860,05	1181,21	708,57	
	T2_1			1032,32	1023,66	1146,06	1055,85	994,17	846,1		699,76	718,65	758,1	775,7	708,31	599,16	
	T7_1			194,04	225,17	210,97	189,65	177,04	140,46		134,49	108,99	156,73	137,87	152,19	98,76	
sigma = 4	T1_1			1033,86	1030,81	1307,18	1208,89	1157,98	1011,9		818,66	937,55	736,99	817,92	1090,19	708,57	
	T2_1			1002,13	994,33	1096,69	1008,99	928,37	846,1		655,24	672,52	708,54	734,11	653,3	599,16	
	T7_1			185,75	213,3	200,14	185,97	168,21	140,46		124,09	104,23	144,61	131,17	139,39	98,76	
Sigma = 5	T1_1			1000,54	1013,9	1244,9	1154,8	1103,91	1011,9		758,87	859,37	700,41	773,53	994,04	708,57	
	T2_1			962,17	961,06	1044,68	956,62	857,8	846,1		605,72	623,29	657,91	684,89	595,92	599,16	
	T7_1			176,83	201,16	189,39	180,02	158,89	140,46		113,6	98,84	132,31	123,35	126,68	98,76	
Sigma = 6	T1_1			958,67	994,06	1183,04	1099,08	1045,33	1011,9		698,49	780,65	662,95	728,46	899,22	708,57	
	T2_1			914,59	925,33	992,41	901,42	787,94	846,1		554,36	573,27	608,5	632,35	540,43	599,16	
	T7_1			167,28	189,11	179,02	172,39	149,32	140,46		103,51	93,31	120,73	114,95	114,62	98,76	
Sigma = 7	T1_1			911,22	971,52	1123,64	1043,55	984,51	1011,9		639,81	705,1	625,03	684,25	810,24	708,57	
	T2_1			862,38	887,99	941,34	846,07	722,32	846,1		504,07	524,43	561,89	580,18	489,25	599,16	
	T7_1			157,35	177,41	169,19	163,66	139,73	140,46		94,2	87,97	110,34	106,56	103,73	98,76	
Sigma = 8	T1_1			861,03	946,3	1067,46	989,37	923,42	1011,9		584,28	635,08	587,04	641,87	729,48	708,57	
	T2_1			808,26	849,65	891,98	792,4	662,52	846,1		456,83	478,18	518,84	530,82	443,43	599,16	
	T7_1			147,33	166,21	159,91	154,37	130,37	140,46		85,88	82,99	101,25	98,64	94,23	98,76	
Sigma = 9	T1_1			810,29	918,28	1014,56	937,12	863,61	1011,9		532,84	571,83	549,55	601,82	657,79	708,57	
	T2_1			754,37	810,79	844,41	741,44	608,86	846,1		413,73	435,49	479,61	485,63	403,24	599,16	
	T7_1			137,49	155,58	151,14	144,99	121,4	140,46		78,58	78,43	93,39	91,48	86,17	98,76	
Sigma = 12	T1_1			667,72	819,02	872,07	793,08	701,56	1011,9		406,41	424,14	446,63	497,05	494,19	708,57	
	T2_1			605,92	694,79	712,45	607,6	480,96	846,1		311,31	332,51	384,22	378,23	313,27	599,16	
	T7_1			110,89	127,47	127,55	118,95	97,89	140,46		62,12	67,09	75,89	75,19	69,39	98,76	

Table S6 shows the activity concentrations measured using WKP and with 0.6 mL small VOI methods on SPECTs images post-filtered with Gaussian filter (0-12 mm) for patient number 6. Whereas, RC represented patient specific recovery coefficient of right and the left kidneys, respectively.

Post-filtering (mm)	Data	Time p.i. (h)	Inj Act (MBq)	Right Kidney (counts/mL)							Left Kidney (counts/mL)						
			7723	VOI_1 (counts/mL)	VOI_2 (counts/mL)	VOI_3 (counts/mL)	VOI_4 (counts/mL)	VOI_5 (counts/mL)	WKP (counts/mL)	RC	VOI_1 (counts/mL)	VOI_2 (counts/mL)	VOI_3 (counts/mL)	VOI_4 (counts/mL)	VOI_5 (counts/mL)	WKP (counts/mL)	RC
	T1_1	23,2		1016,07	958,16	1018,91	1232,08	1036,53	742,34	0,85	1011,9	913,01	1086,03	951,84	812,71	732,77	0,88
sigma = 0	T2_1	47,3		836,48	617,18	750,55	804,03	765,46	554,34		730,98	678,22	809,76	724,74	791,11	547,84	
	T7_1	170,7		179,59	180,3	174,46	176,81	191,41	134,56		175,48	172,65	180,13	195,4	197,19	132,64	
	T1_1			925,48	921,98	969,26	1134,93	951,92	742,34		937,17	871,04	1017,34	876,81	762,2	732,77	
Sigma = 3	T2_1			755,76	601,73	747,61	775,24	689,1	554,34		674,27	644,18	765,19	666,5	721,7	547,84	
	T7_1			164,15	171,86	165,48	171,05	172,52	134,56		162,15	165,41	176,14	185,53	181,57	132,64	
	T1_1			867,99	892,62	935,05	1077	897,46	742,34		886,06	843,3	976,17	835,07	725,47	732,77	
sigma = 4	T2_1			706,54	592,87	735,01	753,8	648,53	554,34		637,89	626,96	735,42	634,88	677,69	547,84	
	T7_1			155,32	166,24	160,74	166,47	160,76	134,56		154,63	160,03	172,14	178,5	171,86	132,64	
	T1_1			808,54	856,97	896,97	1015,06	840,29	742,34		831,06	813,14	933,53	792,45	683,9	732,77	
Sigma = 5	T2_1			656,03	581,97	714,83	727,25	607,34	554,34		599,37	610,82	703,87	603,09	632,73	547,84	
	T7_1			146,61	160,25	156,39	161,08	148,6	134,56		147,12	154,12	166,84	170,82	161,76	132,64	
	T1_1			750,99	817,64	857,06	951,73	784,03	742,34		775,65	782,36	890,84	750,15	640,87	732,77	
Sigma = 6	T2_1			606,98	568,98	689,02	696,36	565,56	554,34		560,86	595,29	672,15	571,81	589,74	547,84	
	T7_1			138,29	154,14	152,38	155,17	136,71	134,56		139,83	148,28	160,76	163,08	151,84	132,64	
	T1_1			697,53	776,62	816,75	889,4	730,83	742,34		722,19	752,41	849,02	709,46	598,68	732,77	
Sigma = 7	T2_1			561,2	554	659,4	662,39	524,52	554,34		523,97	579,88	640,99	541,77	549,97	547,84	
	T7_1			130,48	148,03	148,45	148,94	125,53	134,56		132,95	142,85	154,39	155,52	142,43	132,64	
	T1_1			648,91	735,26	776,9	829,57	681,34	742,34		672,02	724,09	808,59	671,04	558,57	732,77	
Sigma = 8	T2_1			519,42	537,15	627,62	626,73	485,38	554,34		489,55	564,12	610,73	513,3	513,61	547,84	
	T7_1			123,18	141,93	144,34	142,49	115,26	134,56		126,55	137,94	148,04	148,19	133,64	132,64	
	T1_1			605,11	694,45	737,98	773,05	635,51	742,34		625,77	697,61	769,89	635,03	521,04	732,77	
Sigma = 9	T2_1			481,72	518,6	594,93	590,58	448,83	554,34		457,97	547,77	581,55	486,48	480,37	547,84	
	T7_1			116,38	135,83	139,84	135,91	105,96	134,56		120,62	133,47	141,91	141,07	125,45	132,64	
	T1_1			498,05	581,22	628,85	626,13	517,1	742,34		510,79	625,95	665,2	539,91	424,56	732,77	
Sigma = 12	T2_1			390,23	455,81	499,2	486,99	356,3	554,34		379,79	494,68	501,62	415,25	395,4	547,84	
	T7_1			98,56	117,73	123,98	116,01	83,59	134,56		105,14	121,69	125,31	120,84	103,91	132,64	

Table S7 shows the activity concentrations measured using WKP and with 0.6 mL small VOI methods on SPECTs images post-filtered with Gaussian filter (0-12 mm) for patient number 7. Whereas, RC represented patient specific recovery coefficient of right and the left kidneys, respectively.

Post-filtering (mm)	Data	Time p.i. (h)	Inj Act (MBq)	Right Kidney (counts/mL)							Left Kidney (counts/mL)						
			7713	VOI_1 (counts/mL)	VOI_2 (counts/mL)	VOI_3 (counts/mL)	VOI_4 (counts/mL)	VOI_5 (counts/mL)	WKP (counts/mL)	RC	VOI_1 (counts/mL)	VOI_2 (counts/mL)	VOI_3 (counts/mL)	VOI_4 (counts/mL)	VOI_5 (counts/mL)	WKP (counts/mL)	RC
sigma = 0	T1_1	22,3		1418,02	1196,16	1197,68	1156,67	1195,19	815,15	0,86	1411,64	1189,85	1382,49	1470,96	1100,38	755,35	0,86
	T2_1	48,1		560,74	654,32	675,64	705,5	732,54	536,03		788,16	688,37	753,22	699,72	665,89	489,57	
	T7_1	170,2		140,01	155,51	146,69	144,67	122,39	108,46		173,55	130,33	186,14	144,06	170,93	100,9	
Sigma = 3	T1_1			1312,4	1183,79	1161,73	1113,89	1107,64	815,15		1312,07	1153,26	1273,03	1351,03	1029,53	755,35	
	T2_1			545,14	628,89	634,62	654,37	668,79	536,03		731,7	651,02	702,02	653,09	617,52	489,57	
	T7_1			128,09	148,24	140,31	133,28	109,49	108,46		156,54	128,43	165,41	134,08	152,49	100,9	
sigma = 4	T1_1			1245,14	1170,22	1137,07	1080,95	1050,63	815,15		1246,1	1124,22	1207,34	1271,01	985,2	755,35	
	T2_1			534,77	610,06	608,82	621	627,23	536,03		694,46	626,78	667,94	619,5	583,85	489,57	
	T7_1			121,28	142,98	135,82	126,06	102,77	108,46		146,23	126,34	153,73	128,18	140,48	100,9	
Sigma = 5	T1_1			1172,98	1151,3	1108,7	1041,19	988,47	815,15		1173,93	1088,76	1140,3	1184,52	937,67	755,35	
	T2_1			523,41	587,54	581,73	584,75	582,36	536,03		653,38	600,94	630,79	581,83	545,61	489,57	
	T7_1			114,52	137,31	130,96	118,46	96,36	108,46		136,05	123,6	142,55	121,71	128,13	100,9	
Sigma = 6	T1_1			1099,4	1126,63	1077,33	996,8	924,77	815,15		1098,97	1049,02	1075,43	1097,43	888,22	755,35	
	T2_1			511,57	562,38	554,62	547,5	536,84	536,03		610,48	574,96	592,95	543,01	505,24	489,57	
	T7_1			108,07	131,53	126,09	110,9	90,33	108,46		126,48	120,31	132,34	114,83	116,37	100,9	
Sigma = 7	T1_1			1027,26	1095,77	1043,5	950,07	862,65	815,15		1024,3	1006,74	1015,11	1014,11	837,93	755,35	
	T2_1			499,39	535,7	528,25	510,72	492,84	536,03		567,76	549,68	556,23	505,04	464,97	489,57	
	T7_1			102,14	125,7	121,36	103,61	84,7	108,46		117,77	116,58	123,24	107,85	105,59	100,9	
Sigma = 8	T1_1			958,3	1058,77	1007,51	902,79	803,97	815,15		952,23	963,15	960,17	936,93	787,64	755,35	
	T2_1			486,69	508,55	502,83	475,29	451,69	536,03		526,79	525,4	521,69	468,93	426,36	489,57	
	T7_1			96,84	119,88	116,79	96,7	79,46	108,46		109,97	112,53	115,2	101,04	95,89	100,9	
Sigma = 9	T1_1			893,56	1016,48	969,66	856,19	749,61	815,15		884,44	919,14	910,39	866,84	738,09	755,35	
	T2_1			473,21	481,82	478,27	441,7	414,03	536,03		488,61	502,17	489,82	435,1	390,37	489,57	
	T7_1			92,21	114,18	112,35	90,22	74,57	108,46		103,08	108,32	108,14	94,61	87,24	100,9	
Sigma = 12	T1_1			728,49	873,11	848,82	726,27	613,07	815,15		714,88	791,94	784,44	697,2	600,79	755,35	
	T2_1			427,24	410,11	408,95	353,98	322,4	536,03		394,89	438,59	410,19	347,74	301,17	489,57	
	T7_1			82,69	98,93	99,44	73,47	61,95	108,46		87,5	96,14	91,71	78,4	66,95	100,9	

Table S8 shows the activity concentrations measured using WKP and with 0.6 mL small VOI methods on SPECTs images post-filtered with Gaussian filter (0-12 mm) for patient number 8. Whereas, RC represented patient specific recovery coefficient of right and the left kidneys, respectively.

Post-filtering (mm)	Data	Time p.i. (h)	Inj Act (MBq)	Right Kidney							Left Kidney						
			7690	VOI_1 (counts/mL)	VOI_2 (counts/mL)	VOI_3 (counts/mL)	VOI_4 (counts/mL)	VOI_5 (counts/mL)	WKP (counts/mL)	RC	VOI_1 (counts/mL)	VOI_2 (counts/mL)	VOI_3 (counts/mL)	VOI_4 (counts/mL)	VOI_5 (counts/mL)	WKP (counts/mL)	RC
sigma = 0	T1_1	19,5		940,34	798,47	884,49	929,57	944,65	691,31	0,87	1001,89	1035,22	1120,46	968,34	883,98	758,2	0,88
	T2_1	46,7		915,25	700,09	731,01	823,97	618,06	512,72		782,25	776,11	862,74	713,69	675,86	541,35	
	T7_1	169,1		167,6	133,08	145,62	137,06	106,77	99,5		153,75	126,81	123,98	137,47	140,99	96,43	
Sigma = 3	T1_1			886,18	775,6	841,83	879,06	871,97	691,31		959,88	991,78	1069,03	904,22	842,65	758,2	
	T2_1			820,59	676,81	692,13	743,45	566,57	512,72		738,18	750,04	780,35	665,49	631,2	541,35	
	T7_1			148,36	132,51	135,31	129,3	101,01	99,5		136,59	120,8	121,88	127,52	134,04	96,43	
sigma = 4	T1_1			849,19	758,81	816,3	847,91	824,86	691,31		930,54	965,06	1029,68	862,07	810,66	758,2	
	T2_1			763,01	657,53	668,41	695,32	536,35	512,72		707,96	727,76	733,56	634,2	601,03	541,35	
	T7_1			138,36	131,5	129,17	124,1	97,54	99,5		126,83	116,64	119,62	121,15	128,33	96,43	
Sigma = 5	T1_1			807,97	739,95	790,23	815,72	773,94	691,31		895,95	935,81	984,54	815,47	770,87	758,2	
	T2_1			703,91	635,37	643,12	647,38	505,76	512,72		673,16	699,81	686,67	600,3	566,95	541,35	
	T7_1			129	130,21	123,61	118,31	93,79	99,5		117,28	112,03	116,44	114,82	121,02	96,43	
Sigma = 6	T1_1			764,79	720,63	764,96	783,65	721,88	691,31		857,02	904,46	936,98	766,98	725,85	758,2	
	T2_1			646,74	612,55	617,04	601,88	475,68	512,72		635,45	668,75	641,59	565,79	531,02	541,35	
	T7_1			120,4	128,93	119,06	112,32	89,61	99,5		108,28	107,21	112,48	108,87	112,77	96,43	
Sigma = 7	T1_1			721,55	701,95	741,32	752,28	670,97	691,31		815,15	871,98	889,25	719,19	678,56	758,2	
	T2_1			593,79	590,16	590,89	560,02	446,74	512,72		596,76	636,87	599,65	532,11	494,9	541,35	
	T7_1			112,67	127,77	115,53	106,37	84,97	99,5		100,03	102,42	107,98	103,38	104,26	96,43	
Sigma = 8	T1_1			679,57	684,29	719,38	721,7	622,64	691,31		771,83	839,31	842,62	673,82	631,41	758,2	
	T2_1			546,07	568,6	565,16	522,09	419,21	512,72		558,64	605,74	561,32	500,1	459,75	541,35	
	T7_1			105,89	126,69	112,8	100,61	80	99,5		92,62	97,91	103,18	98,28	95,99	96,43	
Sigma = 9	T1_1			639,71	667,46	698,69	691,77	577,57	691,31		728,4	807,08	797,78	631,72	586	758,2	
	T2_1			503,76	547,87	540,11	487,89	393,25	512,72		522,19	576,18	526,56	470,15	426,34	541,35	
	T7_1			100,05	125,55	110,61	95,14	74,9	99,5		86,05	93,79	98,31	93,49	88,22	96,43	
Sigma = 12	T1_1			536,81	617,77	638,4	604,36	462,71	691,31		606,55	714,7	676,88	525,67	467,19	758,2	
	T2_1			405,75	489,13	469,99	403,97	324,94	512,72		427,25	498,22	440,45	392,65	339,92	541,35	
	T7_1			87,54	120,92	105,04	80,77	60,65	99,5		70,85	83,91	84,68	80,26	68,86	96,43	

Table S9 shows the activity concentrations measured using WKP and with 0.6 mL small VOI methods on SPECTs images post-filtered with Gaussian filter (0-12 mm) for patient number 9. Whereas, RC represented patient specific recovery coefficient of right and the left kidneys, respectively.

Post-filtering (mm)	Data	Time p.i. (h)	Inj Act (MBq)	Right Kidney							Left Kidney (counts/mL)						
			7738	VOI_1 (counts/mL)	VOI_2 (counts/mL)	VOI_3 (counts/mL)	VOI_4 (counts/mL)	VOI_5 (counts/mL)	WKP (counts/mL)	RC	VOI_1 (counts/mL)	VOI_2 (counts/mL)	VOI_3 (counts/mL)	VOI_4 (counts/mL)	VOI_5 (counts/mL)	WKP (counts/mL)	RC
sigma = 0	T1_1	4,4		817,27	763,06	883,65	780,29	807,45	530,54	0,86	769,82	898,77	902,3	791,43	633,5	489,49	0,87
	T2_1	51,0		378,87	391,95	473,11	399,16	370,56	279,86		424,8	386,8	428,73	385,53	349,32	249,22	
	T7_1	171,3		92,44	74,73	76,68	78,16	94,98	55,15		95,25	100,27	114,02	72,71	96,73	52,24	
Sigma = 3	T1_1			792,75	715,89	821,79	746,82	755,74	530,54		714,1	817,3	824,22	728,59	609,63	489,49	
	T2_1			351,28	365,28	436,63	379,32	360,59	279,86		393,12	366,75	410,98	362,83	315,02	249,22	
	T7_1			83,81	71,36	73,1	71,68	86,1	55,15		83,69	87,12	97,78	63,99	81,84	52,24	
sigma = 4	T1_1			770,37	688,1	784,38	725,53	720,96	530,54		677,48	765,38	776,98	689,73	588,74	489,49	
	T2_1			333,91	350,42	414,95	365,39	351,85	279,86		372,02	351,43	396,17	348,03	294,44	249,22	
	T7_1			78,37	69,03	70,56	68,45	80,66	55,15		77,34	79,8	90,09	59,79	73,64	52,24	
Sigma = 5	T1_1			742,76	661,04	746	701,59	681,5	530,54		638,35	711,17	728,88	648,94	562,49	489,49	
	T2_1			316,02	335,68	392,48	349,97	340,13	279,86		349,32	333,47	377,7	331,93	274,01	249,22	
	T7_1			72,75	66,45	67,65	65,56	74,94	55,15		71,23	72,81	83,36	56,24	65,93	52,24	
Sigma = 6	T1_1			713,03	636,24	708,04	675,42	639,72	530,54		599,04	658,37	682,2	608,61	532,9	489,49	
	T2_1			298,75	321,65	370,23	334,27	326,14	279,86		326,48	314,35	357,13	315,09	254,9	249,22	
	T7_1			67,34	63,82	64,64	63,01	69,23	55,15		65,56	66,47	77,42	53,29	59,07	52,24	
Sigma = 7	T1_1			683,72	614,09	671,48	647,78	597,8	530,54		561,04	609,3	638,18	570,29	501,86	489,49	
	T2_1			282,77	308,75	349,04	319	310,66	279,86		304,46	295,26	335,9	298,06	237,6	249,22	
	T7_1			62,44	61,33	61,73	60,69	63,78	55,15		60,41	60,92	72,13	50,78	53,22	52,24	
Sigma = 8	T1_1			656,54	594,42	636,85	619,44	557,31	530,54		525,11	564,95	597,27	534,65	470,81	489,49	
	T2_1			268,45	297,22	329,38	304,45	294,45	279,86		283,76	277,03	315,04	281,31	222,04	249,22	
	T7_1			58,17	59,1	59,04	58,45	58,72	55,15		55,81	56,16	67,35	48,55	48,34	52,24	
Sigma = 9	T1_1			632,35	576,93	604,39	591,03	519,16	530,54		491,53	525,41	559,5	501,85	440,74	489,49	
	T2_1			255,95	287,14	311,43	290,67	278,14	279,86		264,67	260,07	295,18	265,18	208,01	249,22	
	T7_1			54,56	57,16	56,6	56,2	56,11	55,15		51,75	52,11	62,98	46,47	44,27	52,24	
Sigma = 12	T1_1			578,09	535,6	520,59	509,47	421,45	530,54		405,02	431,28	463,39	418,64	360,59	489,49	
	T2_1			228,95	264,75	267,82	253,73	232,45	279,86		217,52	217,56	243,72	222,27	172,92	249,22	
	T7_1			47,32	52,89	50,62	49,09	42,92	55,15		42,58	43,15	51,86	40,56	35,42	52,24	

Table S10 shows the activity concentrations measured using WKP and with 0.6 mL small VOI methods on SPECTs images post-filtered with Gaussian filter (0-12 mm) for patient number 10. Whereas, RC represented patient specific recovery coefficient of right and the left kidneys, respectively.

Post-filtering (mm)	Data	Time p.i. (h)	Inj Act (MBq)	Right Kidney							Left Kidney						
			7738	VOI_1 (counts/mL)	VOI_2 (counts/mL)	VOI_3 (counts/mL)	VOI_4 (counts/mL)	VOI_5 (counts/mL)	WKP (counts/mL)	RC	VOI_1 (counts/mL)	VOI_2 (counts/mL)	VOI_3 (counts/mL)	VOI_4 (counts/mL)	VOI_5 (counts/mL)	WKP (counts/mL)	RC
sigma = 0	T1_1	22,2		1035,21	845,93	1060,88	950,18	1040,37	670,81	0,83	963,86	1047,67	960,5	775,93	720,14	717,2	0,82
	T2_1	119,1		172,13	158,37	173,31	179,91	149,41	126,71		173,47	134,47	122,7	132,51	159,14	123,39	
	T7_1	173,2		75	87,46	82,3	67,71	81,06	58,98		52,84	57,69	47,67	54,28	51,03	60,76	
Sigma = 3	T1_1			932,2	812,33	999,46	867,44	910,85	670,81		886,56	976,35	904	733,87	678,16	717,2	
	T2_1			163,38	146,93	161,03	169,84	139,46	126,71		161,17	129,96	114,12	123,7	149,04	123,39	
	T7_1			66,8	76,86	75,26	66,54	74,53	58,98		53,16	60,67	49,8	53,57	52,5	60,76	
sigma = 4	T1_1			871,96	789,3	956,6	815,55	837,31	670,81		837,7	932,38	868,98	704,9	647,47	717,2	
	T2_1			157,21	139,98	153,43	162,28	133,09	126,71		153,1	126,06	109,3	117,94	141,32	123,39	
	T7_1			63,37	71,62	70,67	64,47	70,1	58,98		53,42	61,32	50,57	52,91	53,23	60,76	
Sigma = 5	T1_1			810,35	762,64	908,29	760,77	765,78	670,81		786,26	887,54	832,39	672,75	612,49	717,2	
	T2_1			150,06	132,9	145,83	153,51	126,26	126,71		144,66	121,75	104,46	112,1	132,96	123,39	
	T7_1			60,76	67,03	65,85	61,58	65,42	58,98		53,72	61,07	51,07	52	53,6	60,76	
Sigma = 6	T1_1			749,44	733,57	857,42	706,59	699,2	670,81		735,32	844,6	795,97	640,01	575,98	717,2	
	T2_1			142,38	126,04	138,54	144,34	119,42	126,71		136,25	117,69	99,92	106,63	124,66	123,39	
	T7_1			58,76	63,09	61,09	58,25	60,08	58,98		54,11	60,21	51,35	50,83	53,48	60,76	
Sigma = 7	T1_1			691,14	703,23	806,32	655,44	639,2	670,81		686,76	805,06	760,83	608,65	539,95	717,2	
	T2_1			134,48	119,57	131,66	135,31	112,79	126,71		128,22	114,29	95,98	101,71	116,82	123,39	
	T7_1			57,17	59,71	56,64	54,78	56,44	58,98		54,5	59,07	51,51	49,44	52,83	60,76	
Sigma = 8	T1_1			636,7	672,38	756,53	608,45	586,11	670,81		641,46	769,22	727,46	579,61	505,57	717,2	
	T2_1			126,59	113,56	125,18	126,76	106,43	126,71		120,81	111,66	92,75	97,32	109,57	123,39	
	T7_1			55,8	56,77	52,6	51,39	52,41	58,98		54,83	57,9	51,58	47,86	51,74	60,76	
Sigma = 9	T1_1			586,73	641,52	708,96	565,89	539,53	670,81		599,68	736,67	696,05	553,16	473,46	717,2	
	T2_1			118,87	108,05	119,07	118,81	100,39	126,71		114,11	109,75	90,21	93,35	102,96	123,39	
	T7_1			54,54	54,18	49,02	48,19	48,75	58,98		55,02	56,9	51,61	46,17	50,33	60,76	
Sigma = 12	T1_1			464,19	551,94	582,9	462,14	431,77	670,81		494,08	651,62	612,91	487,14	391,7	717,2	
	T2_1			97,91	93,98	102,61	98,66	84,43	126,71		98,08	106,5	85,72	82,93	86,43	123,39	
	T7_1			50,68	47,72	40,66	40,07	39,75	58,98		54,56	55,58	51,6	40,99	45,19	60,76	

Table S11 shows the activity concentrations measured using WKP and with 0.6 mL small VOI methods on SPECTs images post-filtered with Gaussian filter (0-12 mm) for patient number 11. Whereas, RC represented patient specific recovery coefficient of right and the left kidneys, respectively.

Post-filtering (mm)	Data	Time p.i. (h)	Inj Act (MBq)	Right Kidney							Left Kidney						
			7736	VOI_1 (counts/mL)	VOI_2 (counts/mL)	VOI_3 (counts/mL)	VOI_4 (counts/mL)	VOI_5 (counts/mL)	WKP (counts/mL)	RC	VOI_1 (counts/mL)	VOI_2 (counts/mL)	VOI_3 (counts/mL)	VOI_4 (counts/mL)	VOI_5 (counts/mL)	WKP (counts/mL)	RC
sigma = 0	T1_1	22,7		745,37	736,82	852,93	782,57	842,84	609,22	0,87	806,51	845,44	745,29	678	703,96	578,46	0,86
	T2_1	119,7		155,15	169,86	206,25	185,81	179,97	120,09		171,57	159,58	161,16	199	132,74	120,57	
	T7_1	172,1		98,52	82,72	106,81	146,3	114,54	69,89		93,3	107,69	141,93	112,53	107,85	66,14	
Sigma = 3	T1_1			745,37	736,82	852,93	782,57	842,84	609,22		806,51	845,44	745,29	678	703,96	578,46	
	T2_1			154,28	164,74	182,59	168,13	156,61	120,09		156,1	151,17	150,87	173,57	136,88	120,57	
	T7_1			90,69	81,16	97,26	123,1	102,89	69,89		87,68	103,44	123,66	99,21	95,63	66,14	
sigma = 4	T1_1			684,93	700,35	780,65	710,81	710,64	609,22		703,91	736,34	676,14	634,88	617,39	578,46	
	T2_1			151,34	162,19	171,95	158,1	143,24	120,09		146,43	145,96	144,06	160,29	135,97	120,57	
	T7_1			85,17	80,13	92,65	111,76	96,27	69,89		83,92	100,49	114,06	92,24	92,24	66,14	
Sigma = 5	T1_1			654,02	682,32	750,86	677,63	658,52	609,22		659,05	692,74	648,88	612,93	580,92	578,46	
	T2_1			146,72	159,34	163,33	148,26	130,75	120,09		136,44	140,36	137,02	148,11	132,38	120,57	
	T7_1			80,51	79,01	88,7	101,56	89,66	69,89		79,78	97,39	105,39	85,71	81,27	66,14	
Sigma = 6	T1_1			620,31	662,36	720,4	642,89	607,85	609,22		612,52	650,45	621,08	588,5	542,82	578,46	
	T2_1			141,04	155,83	156,13	138,89	119,82	120,09		126,76	134,7	130,21	137,25	126,8	120,57	
	T7_1			75,47	77,85	85,4	92,56	83,24	69,89		75,52	94,38	97,9	79,79	74,5	66,14	
Sigma = 7	T1_1			585,48	641,19	689,99	608,35	560,51	609,22		566,66	611,51	593,21	562,73	504,46	578,46	
	T2_1			134,84	151,68	149,69	130,18	110,47	120,09		117,77	129,28	123,91	127,61	120,1	120,57	
	T7_1			70,86	76,67	82,65	84,74	77,11	69,89		71,33	91,6	91,55	74,51	68,29	66,14	
Sigma = 8	T1_1			550,77	619,15	659,91	575,11	517,29	609,22		523,14	576,8	565,59	536,34	467,08	578,46	
	T2_1			128,48	147	143,59	122,15	102,38	120,09		109,64	124,31	118,14	118,95	112,97	120,57	
	T7_1			66,79	75,44	80,29	77,98	71,31	69,89		67,38	89,05	86,14	68,8	62,67	66,14	
Sigma = 9	T1_1			517,12	596,32	630,3	543,67	478,31	609,22		482,95	546,34	538,53	509,76	431,6	578,46	
	T2_1			122,17	141,99	137,67	114,84	95,24	120,09		102,38	119,84	112,83	111,05	105,87	120,57	
	T7_1			63,27	74,13	78,18	72,14	65,88	69,89		63,75	86,68	81,43	65,56	57,63	66,14	
Sigma = 12	T1_1			427,45	524,18	545,83	460,56	384,2	609,22		384,83	475,01	463,25	431,68	340,73	578,46	
	T2_1			104,66	126,39	121,07	96,85	77,78	120,09		85,31	108,94	98,6	90,8	86,76	120,57	
	T7_1			55,29	69,6	72,52	59,15	52,03	69,89		55,21	79,85	70,01	54,97	45,6	66,14	

Table S12 shows the activity concentrations measured using WKP and with 0.6 mL small VOI methods on SPECTs images post-filtered with Gaussian filter (0-12 mm) for patient number 12. Whereas, RC represented patient specific recovery coefficient of right and the left kidneys, respectively.

Post-filtering (mm)	Data	Time p.i. (h)	Inj Act (MBq)	Right Kidney (counts/mL)							Left Kidney (counts/mL)						
			7616	VOI_1 (counts/mL)	VOI_2 (counts/mL)	VOI_3 (counts/mL)	VOI_4 (counts/mL)	VOI_5 (counts/mL)	WKP (counts/mL)	RC	VOI_1 (counts/mL)	VOI_2 (counts/mL)	VOI_3 (counts/mL)	VOI_4 (counts/mL)	VOI_5 (counts/mL)	WKP (counts/mL)	RC
sigma = 0	T1_1	22,9		549,56	696,56	560,49	669,97	566,57	409,6	0,85	702,67	655,92	685,67	726,25	523,01	418,29	0,84
	T2_1	50,1		380,74	380,21	411,66	339,18	316,13	260,19		418,7	423,47	424,72	425,39	331,03	251,38	
	T7_1	173,0		64,13	54,62	46,53	41,56	41,55	35,25		45,44	57,14	81,43	71,11	67,58	34,67	
Sigma = 3	T1_1			496,11	633,06	543,51	632,62	541,94	409,6		627,13	615,41	643,11	669,16	483,12	418,29	
	T2_1			337,31	359,83	381,78	328,7	297,45	260,19		375,35	399,49	391,46	391,38	308,4	251,38	
	T7_1			54,79	51,3	45,05	38,21	36,75	35,25		45,17	52,7	70,26	61,2	60,23	34,67	
sigma = 4	T1_1			462,16	594,35	531,72	604,75	521,99	409,6		584,81	589,26	611,09	629,69	458,23	418,29	
	T2_1			312,1	345,28	363,21	320,11	284,35	260,19		351,45	382,24	370,03	370,29	294,68	251,38	
	T7_1			49,76	49,47	44,44	36,4	33,9	35,25		44,22	49,92	64,05	56,13	56,15	34,67	
Sigma = 5	T1_1			427,34	554,41	517,27	572,58	497,65	409,6		543,97	560,94	575,7	586,92	431,97	418,29	
	T2_1			287,46	328,45	343,85	309,02	269,66	260,19		328,27	362,3	347,32	347,86	279,99	251,38	
	T7_1			45,15	47,67	43,96	34,73	31,11	35,25		42,6	47,05	58,3	51,6	52,18	34,67	
Sigma = 6	T1_1			394,39	515,58	500,27	538,44	470,8	409,6		506,03	531,58	539,64	544,04	405,78	418,29	
	T2_1			264,78	310,44	324,72	295,8	254,44	260,19		306,51	340,97	324,74	325,28	264,88	251,38	
	T7_1			41,15	45,84	43,52	33,27	28,54	35,25		40,52	44,29	53,26	47,61	48,43	34,67	
Sigma = 7	T1_1			364,94	479,45	481,09	504,3	443,15	409,6		471,49	501,94	504,84	503,24	380,59	418,29	
	T2_1			244,77	292,26	306,39	281,17	239,57	260,19		286,51	319,52	303,22	303,55	249,81	251,38	
	T7_1			37,81	43,97	42,98	32,04	26,29	35,25		38,26	41,75	48,97	44,11	44,97	34,67	
Sigma = 8	T1_1			339,64	446,76	460,34	471,45	416	409,6		440,28	472,63	472,24	465,62	356,81	418,29	
	T2_1			227,63	274,64	289,08	265,92	225,58	260,19		268,35	298,81	283,25	283,22	235,03	251,38	
	T7_1			35,06	42,05	42,22	30,98	24,41	35,25		36,04	39,45	45,31	41,01	41,78	34,67	
Sigma = 9	T1_1			318,47	417,67	438,73	440,57	390,16	409,6		412,13	444,19	442,12	431,51	334,54	418,29	
	T2_1			213,24	258,04	272,77	250,75	212,74	260,19		251,97	279,35	264,94	264,5	220,75	251,38	
	T7_1			32,8	40,1	41,21	30,05	22,88	35,25		34,01	37,37	42,14	38,22	38,86	34,67	
Sigma = 12	T1_1			274,81	349,01	374,39	361,53	323,64	409,6		343,22	367,83	365,46	348,31	276,24	418,29	
	T2_1			183,13	215,79	229,26	209,42	180,65	260,19		212,4	230,06	219,15	217,23	181,83	251,38	
	T7_1			28,07	34,44	36,86	27,54	19,96	35,25		29,4	32,26	34,61	31,33	31,45	34,67	

Table S13 shows the activity concentrations measured using WKP and with 0.6 mL small VOI methods on SPECTs images post-filtered with Gaussian filter (0-12 mm) for patient number 13. Whereas, RC represented patient specific recovery coefficient of right and the left kidneys, respectively.

Post-filtering (mm)	Data	Time p.i. (h)	Inj Act (MBq)	Right Kidney							Left Kidney						
			7589	VOI_1 (counts/mL)	VOI_2 (counts/mL)	VOI_3 (counts/mL)	VOI_4 (counts/mL)	VOI_5 (counts/mL)	WKP (counts/mL)	RC	VOI_1 (counts/mL)	VOI_2 (counts/mL)	VOI_3 (counts/mL)	VOI_4 (counts/mL)	VOI_5 (counts/mL)	WKP (counts/mL)	RC
sigma = 0	T1_1	22,9		585,94	504,3	635,12	579,1	603,47	353,04	0,85	569,83	565,71	558,92	563,2	490,98	330,61	0,84
	T2_1	50,3		428,02	404,6	504,91	471,32	481,58	290,42		448,17	493,14	466,26	421,56	468,25	259,69	
	T7_1	171,8		133,82	124,05	194,06	162,03	112,39	88,37		146,44	181,09	151,03	149,3	146,36	86,14	
Sigma = 3	T1_1			533,18	470,77	580,11	549,69	535,79	353,04		512,64	508,85	511,96	518,53	452,17	330,61	
	T2_1			408,49	380,85	467,75	440,93	435,37	290,42		396,93	441,94	432,3	398,42	420,52	259,69	
	T7_1			121,19	114,31	164,27	146,64	113,15	88,37		127,28	154,18	133,38	139,12	125,62	86,14	
sigma = 4	T1_1			499,95	447,01	545,07	527,76	496,9	353,04		478,26	476,18	484,07	487,66	426,77	330,61	
	T2_1			389,02	362,89	438,66	418,35	407,58	290,42		365,13	411,23	409,71	378,62	391,95	259,69	
	T7_1			112,82	107,87	148,12	136,9	112,17	88,37		118,03	139,65	124,08	131,52	114,36	86,14	
Sigma = 5	T1_1			465,1	420,1	507,75	501,37	458,9	353,04		443,67	444,55	455,75	454,43	399,25	330,61	
	T2_1			364,76	342,81	405,79	392,85	378,7	290,42		333,38	380,04	384,82	356,91	363,78	259,69	
	T7_1			103,83	101,09	133,14	126,75	109,88	88,37		110,27	126,05	115,43	122,89	103,79	86,14	
Sigma = 6	T1_1			430,73	392,04	470,17	472,12	423,3	353,04		410,72	414,95	428,11	421,21	371,23	330,61	
	T2_1			338,24	322,38	372,27	366,61	350,41	290,42		304,1	350,3	359,29	335,47	337,3	259,69	
	T7_1			94,91	94,49	120	116,8	106,4	88,37		104,21	113,91	107,57	114,08	94,44	86,14	
Sigma = 7	T1_1			398,07	364,49	434,02	441,81	390,75	353,04		380,76	387,79	401,64	389,44	344,02	330,61	
	T2_1			311,53	302,48	340,29	341,15	323,76	290,42		278,85	323,31	334,28	315,04	312,93	259,69	
	T7_1			86,54	88,3	108,81	107,45	101,97	88,37		99,83	103,53	100,49	105,66	86,44	86,14	
Sigma = 8	T1_1			367,64	338,51	400,28	411,85	361,28	353,04		354,48	363,21	376,47	359,82	318,34	330,61	
	T2_1			285,94	283,52	310,99	317,22	299,25	290,42		258,26	299,74	310,5	295,74	290,66	259,69	
	T7_1			78,79	82,85	99,32	98,89	96,92	88,37		96,86	94,92	94,08	97,9	79,69	86,14	
Sigma = 9	T1_1			339,62	314,66	369,42	383,16	334,68	353,04		332,16	341,23	352,62	332,61	294,46	330,61	
	T2_1			262,15	265,67	284,78	295,03	276,97	290,42		242,32	279,79	288,37	277,51	270,31	259,69	
	T7_1			72,26	77,33	91,18	91,19	91,53	88,37		94,93	87,98	88,2	90,83	73,93	86,14	
Sigma = 12	T1_1			270,2	257,03	294,28	308,31	269,1	353,04		288,92	290,81	289,48	265,33	233,73	330,61	
	T2_1			203,51	218,97	223,63	238,56	222,2	290,42		218,31	239,14	233,49	228,94	219,13	259,69	
	T7_1			56,86	64,12	72,37	72,51	75,38	88,37		91,92	74,81	73,11	73,3	60,45	86,14	

Table S14 shows the activity concentrations measured using WKP and with 0.6 mL small VOI methods on SPECTs images post-filtered with Gaussian filter (0-12 mm) for patient number 14. Whereas, RC represented patient specific recovery coefficient of right and the left kidneys, respectively.

Post-filtering (mm)	Data	Time p.i. (h)	Inj Act (MBq)	Right Kidney							Left Kidney						
			7614	VOI_1 (counts/mL)	VOI_2 (counts/mL)	VOI_3 (counts/mL)	VOI_4 (counts/mL)	VOI_5 (counts/mL)	WKP (counts/mL)	RC	VOI_1 (counts/mL)	VOI_2 (counts/mL)	VOI_3 (counts/mL)	VOI_4 (counts/mL)	VOI_5 (counts/mL)	WKP (counts/mL)	RC
sigma = 0	T1_1	24,5		758,11	669,93	719,41	763,03	749,42	486,91	0,87	807,39	564,21	649,77	693,76	680,39	470	0,83
	T2_1	51,2		429,97	510,28	467,88	509,78	510,86	346,22		483,26	401,86	425,76	397,88	489,65	316,25	
	T7_1	172,5		101,56	76,56	100,41	100,3	72,5	57,37		120,83	74,06	87,53	106,77	96,68	55,8	
Sigma = 3	T1_1			717,07	639,7	681,48	707,2	689,36	486,91		745,09	560,09	640,6	656,04	645,53	470	
	T2_1			398,64	456,32	448,91	486,5	492,72	346,22		450,51	377,18	391,49	373,92	448,97	316,25	
	T7_1			97,41	78,22	91,32	89,01	73,26	57,37		109,56	74,69	78,69	86,59	90,44	55,8	
sigma = 4	T1_1			686,42	618,57	656	674,04	651,74	486,91		705,4	556,96	628,31	628,75	617,11	470	
	T2_1			378,85	426,58	434,04	468,33	476,43	346,22		428,55	362,05	370,98	359,74	422,39	316,25	
	T7_1			93,34	77,93	85,74	83,16	72,54	57,37		102,03	74,23	74,6	78,91	85,28	55,8	
Sigma = 5	T1_1			650,44	595,11	628,03	640,14	612,3	486,91		662,62	552	609,96	597,43	582,45	470	
	T2_1			357,8	398,79	416,06	447,11	455,38	346,22		403,89	346,83	350,45	345,26	393,36	316,25	
	T7_1			87,95	76,56	80,22	77,86	70,52	57,37		93,98	73,03	70,9	72,99	78,9	55,8	
Sigma = 6	T1_1			611,52	571,1	599,73	606,57	572,85	486,91		618,36	544,11	586,74	563,93	544,31	470	
	T2_1			336,49	374,02	396,44	424,72	430,99	346,22		377,64	332,45	331,18	330,9	363,37	316,25	
	T7_1			81,75	74,4	75,12	73,21	67,33	57,37		86,02	71,29	67,43	68,2	72,03	55,8	
Sigma = 7	T1_1			571,75	547,52	572,32	573,77	534,6	486,91		574,17	532,41	560,36	529,95	505,13	470	
	T2_1			315,64	352,54	376,56	402,47	404,85	346,22		350,97	319,15	313,82	316,67	333,88	316,25	
	T7_1			75,28	71,69	70,65	69,08	63,38	57,37		78,6	69,11	64,12	64,12	65,24	55,8	
Sigma = 8	T1_1			532,69	524,52	546,15	541,9	498,17	486,91		531,23	516,64	532,37	496,6	466,65	470	
	T2_1			295,67	333,88	357,32	380,94	378,25	346,22		324,86	306,64	298,38	302,4	305,92	316,25	
	T7_1			69	68,63	66,79	65,29	59,07	57,37		71,92	66,61	60,95	60,45	58,89	55,8	
Sigma = 9	T1_1			495,37	501,88	521,03	511,09	463,9	486,91		490,42	497,14	503,88	464,48	429,98	470	
	T2_1			276,83	317,33	339,15	360,22	352,11	346,22		300,03	294,44	284,49	287,99	280,08	316,25	
	T7_1			63,18	65,36	63,43	61,65	54,72	57,37		65,99	63,87	57,92	57,02	53,15	55,8	
Sigma = 12	T1_1			398,2	433,83	448,7	425,83	374,85	486,91		384,7	424,78	420,94	377,52	335,27	470	
	T2_1			227,93	274,39	290,7	302,13	281,2	346,22		236,34	256,97	247,78	244,61	216,81	316,25	
	T7_1			49,15	55,4	54,92	51,2	43,02	57,37		52,04	54,99	49,62	47,57	39,77	55,8	

Table S15 shows the activity concentrations measured using WKP and with 0.6 mL small VOI methods on SPECTs images post-filtered with Gaussian filter (0-12 mm) for patient number 15. Whereas, RC represented patient specific recovery coefficient of right and the left kidneys, respectively.

Post-filtering (mm)	Data	Time p.i. (h)	Inj Act (MBq)	Right Kidney							Left Kidney						
			7620	VOI_1 (counts/mL)	VOI_2 (counts/mL)	VOI_3 (counts/mL)	VOI_4 (counts/mL)	VOI_5 (counts/mL)	WKP (counts/mL)	RC	VOI_1 (counts/mL)	VOI_2 (counts/mL)	VOI_3 (counts/mL)	VOI_4 (counts/mL)	VOI_5 (counts/mL)	WKP (counts/mL)	RC
sigma = 0	T1_1	20,7		721,55	596,4	648,61	607,65	585,06	419,72	0,85	582,18	579,18	607,91	639,65	569,96	426,56	0,84
	T2_1	48,4		392,95	327,79	378,57	396,76	415,99	243,98		407,54	378,76	375,93	391,91	333,46	235,34	
	T7_1	169,1		73,81	48,55	60,12	46,94	56,89	41,39		49,13	82,04	61,68	57,01	51,09	42,58	
Sigma = 3	T1_1			674,81	565,09	601,5	577,54	533,74	419,72		528,23	536,92	588,93	593,8	538,35	426,56	
	T2_1			354,72	305,49	361,68	367,17	368,35	243,98		372,14	349,7	350,02	355,39	305,22	235,34	
	T7_1			63,66	45,71	54,06	46,23	48,63	41,39		45,02	70,32	56,91	57,74	48,75	42,58	
sigma = 4	T1_1			638,07	543,17	571,78	553,22	501,29	419,72		494,16	510,74	571,33	565,68	517,29	426,56	
	T2_1			330,94	293,39	349,26	348,56	342,63	243,98		350,52	332,63	334,73	334,05	289,79	235,34	
	T7_1			58,07	43,93	51,15	45,29	44,17	41,39		42,3	64,29	53,88	57,01	46,88	42,58	
Sigma = 5	T1_1			594,86	518,59	540,3	524,21	467,51	419,72		458,77	484,09	549,09	535,49	494,5	426,56	
	T2_1			305,99	281,69	334,34	329,05	317,81	243,98		328,18	315,17	318,77	312,52	274,76	235,34	
	T7_1			52,7	42,04	48,54	44,09	39,92	41,39		39,46	58,92	50,69	55,3	44,57	42,58	
Sigma = 6	T1_1			549,09	493,05	508,54	493,02	434,63	419,72		424,17	458,58	523,98	504,08	471,27	426,56	
	T2_1			281,45	270,55	317,66	309,55	294,27	243,98		305,95	297,93	302,48	291,67	260,31	235,34	
	T7_1			47,78	40,18	46,09	42,67	36,06	41,39		36,77	54,26	47,46	52,81	41,94	42,58	
Sigma = 7	T1_1			503,79	467,49	477,38	461,75	403,76	419,72		391,64	434,8	497,42	472,65	448,19	426,56	
	T2_1			258,32	259,85	300,13	290,61	272,36	243,98		284,47	281,38	286,21	272,05	246,47	235,34	
	T7_1			43,45	38,44	43,76	41,07	32,69	41,39		34,36	50,24	44,32	49,88	39,16	42,58	
Sigma = 8	T1_1			460,88	442,52	447,41	431,75	375,26	419,72		361,76	412,71	470,5	442,21	425,43	426,56	
	T2_1			237,05	249,34	282,56	272,53	252,2	243,98		264,13	265,8	270,23	253,91	233,12	235,34	
	T7_1			39,72	36,88	41,54	39,29	29,8	41,39		32,27	46,74	41,34	46,77	36,4	42,58	
Sigma = 9	T1_1			421,34	418,5	419,07	403,72	349,1	419,72		334,67	392,07	444,02	413,4	403,03	426,56	
	T2_1			217,77	238,78	265,51	255,44	233,78	243,98		245,09	251,3	254,76	237,29	220,18	235,34	
	T7_1			36,55	35,49	39,44	37,38	27,32	41,39		30,5	43,69	38,56	43,69	33,74	42,58	
Sigma = 12	T1_1			324,79	353,65	345,73	332,27	282,91	419,72		268,77	336,48	371,13	338,81	338,52	426,56	
	T2_1			171,16	206,15	219,91	210,47	187,74	243,98		196,23	213,9	212,71	195,58	183,68	235,34	
	T7_1			29,7	32,05	33,86	31,42	21,66	41,39		26,62	36,45	31,53	35,29	26,85	42,58	

Table S16 shows the activity concentrations measured using WKP and with 0.6 mL small VOI methods on SPECTs images post-filtered with Gaussian filter (0-12 mm) for patient number 16. Whereas, RC represented patient specific recovery coefficient of right and the left kidneys, respectively.

Post-filtering (mm)	Data	Time p.i. (h)	Inj Act (MBq)	Right Kidney							Left Kidney						
			7488	VOI_1 (counts/mL)	VOI_2 (counts/mL)	VOI_3 (counts/mL)	VOI_4 (counts/mL)	VOI_5 (counts/mL)	WKP (counts/mL)	RC	VOI_1 (counts/mL)	VOI_2 (counts/mL)	VOI_3 (counts/mL)	VOI_4 (counts/mL)	VOI_5 (counts/mL)	WKP (counts/mL)	RC
sigma = 0	T1_1	23,2		676,45	626,19	661,62	568,16	677,94	440,12	0,86	682,47	685,11	721,1	674,37	568,03	410,14	0,85
	T2_1	50,2		441,3	330,46	366,17	365,78	421,42	235,88		367,54	305,69	285,24	460,73	362,74	254,2	
	T7_1	171,3		56,15	58,24	77,26	55,44	87,73	45,27		90,56	58,81	87,36	72,43	96,96	44,04	
Sigma = 3	T1_1			646,36	596,29	626,37	534,04	632,76	440,12		582,87	613,37	652,86	633,09	543,32	410,14	
	T2_1			406,68	313,87	349,58	355,46	408,96	235,88		315,7	269,87	269,6	418,75	353,62	254,2	
	T7_1			56,97	60,9	70,97	52,5	82,7	45,27		75,01	56,14	71,95	65,86	78,71	44,04	
sigma = 4	T1_1			625,14	575,45	602,32	514,63	602,85	440,12		525,03	572,45	612,49	605,6	521,78	410,14	
	T2_1			383,98	305,2	338,06	343,93	396,38	235,88		286,5	249,39	260,38	392,18	344,41	254,2	
	T7_1			56,72	61,4	66,88	50,99	78,29	45,27		66,61	53,94	64,67	62,27	69,85	44,04	
Sigma = 5	T1_1			600,23	552,67	575,3	494,28	569,91	440,12		468,12	532,38	572,17	575,26	494,88	410,14	
	T2_1			359,87	297,26	325,13	328,97	379,6	235,88		258,17	229,52	250,98	364,58	332,19	254,2	
	T7_1			56,02	61,07	62,7	49,39	73,25	45,27		58,85	51,2	58,85	58,91	62,33	44,04	
Sigma = 6	T1_1			572,8	529,84	546,83	473,1	535,75	440,12		415,34	494,66	533,57	543,55	465,16	410,14	
	T2_1			335,94	290,12	311,53	312,7	359,97	235,88		231,74	211,09	241,5	337,74	317,66	254,2	
	T7_1			55	60,06	58,81	47,63	68,14	45,27		52,02	48,14	53,53	55,85	56,19	44,04	
Sigma = 7	T1_1			543,97	508,01	518,24	451,66	501,88	440,12		368,37	460,03	497,54	511,87	434,83	410,14	
	T2_1			313,26	283,48	297,78	296,52	338,88	235,88		207,95	194,55	232,03	312,87	301,54	254,2	
	T7_1			53,71	58,57	55,35	45,76	63,28	45,27		46,27	44,96	49,34	53,04	51,23	44,04	
Sigma = 8	T1_1			514,73	487,43	490,46	430,55	469,33	440,12		327,58	428,53	464,31	481,21	405,44	410,14	
	T2_1			292,33	276,86	284,29	281,09	317,49	235,88		187,1	179,98	222,62	290,42	284,55	254,2	
	T7_1			52,17	56,75	52,28	43,81	58,77	45,27		41,58	41,84	45,77	50,37	47,16	44,04	
Sigma = 9	T1_1			485,81	467,93	464,11	410,19	438,7	440,12		292,76	399,92	433,83	452,16	377,87	410,14	
	T2_1			273,25	269,84	271,32	266,64	296,64	235,88		169,18	167,3	213,32	270,42	267,33	254,2	
	T7_1			50,41	54,74	49,53	41,83	54,66	45,27		37,84	38,91	42,68	47,75	43,72	44,04	
Sigma = 12	T1_1			405,78	412,72	395,52	355,46	360,4	440,12		218,43	328,8	357,34	375,88	307,88	410,14	
	T2_1			226,15	244,73	236,49	228,4	241,14	235,88		130,75	138,67	186,49	222,32	218,62	254,2	
	T7_1			44,37	48,34	42,66	36,12	44,43	45,27		30,88	31,69	35,38	40,18	35,58	44,04	

Post-filtering (mm)

Post-filtering (mm)	Data	Time p.i. (h)	Inj Act (MBq)	Right Kidney (counts/mL)							Left Kidney (counts/mL)						
			7584	VOI_1 (counts/mL)	VOI_2 (counts/mL)	VOI_3 (counts/mL)	VOI_4 (counts/mL)	VOI_5 (counts/mL)	WKP (counts/mL)	RC	VOI_1 (counts/mL)	VOI_2 (counts/mL)	VOI_3 (counts/mL)	VOI_4 (counts/mL)	VOI_5 (counts/mL)	WKP (counts/mL)	RC
sigma = 0	T1_1	23,0		640,21	798,76	813,58	827,13	774,62	624,72	0,84	877,49	851,84	856,69	968,81	928,39	692,25	0,85
	T2_1	50,8		580,61	607,19	624,77	570	562,09	423,35		624,35	519,73	558,63	536,32	595,74	463,18	
	T7_1	171,7		107,68	87,06	106,98	94,96	98,25	71,03		105,34	94,55	113,6	94,12	101,19	70,29	
Sigma = 3	T1_1			614,27	740,85	780,03	790,08	735,08	624,72		827,56	819,23	814,02	894,5	853,27	692,25	
	T2_1			524,81	575,98	595,69	542,05	514,64	423,35		589,8	481,75	524,83	521,13	542,31	463,18	
	T7_1			99,62	83,64	100,01	95,11	93,39	71,03		93,8	91,2	105,97	85,88	91,46	70,29	
sigma = 4	T1_1			595,44	707,2	757,21	761,6	706,13	624,72		791,56	798,46	788,26	847,77	805,4	692,25	
	T2_1			494,04	553,55	574,58	521,44	484,18	423,35		564,83	460,18	503,79	507,14	512,45	463,18	
	T7_1			94,16	81,05	94,73	93,22	89,74	71,03		87,2	89,05	100,41	80,74	85,2	70,29	
Sigma = 5	T1_1			573,09	673,77	731,38	728,49	672,18	624,72		749,86	776,4	761,48	798,8	755,12	692,25	
	T2_1			464,36	527,91	549,99	496,79	451,55	423,35		535,44	439,5	482,07	488,47	483,33	463,18	
	T7_1			88,13	78,25	88,87	89,9	85,26	71,03		80,79	86,87	94,16	75,48	78,66	70,29	
Sigma = 6	T1_1			548,49	641,99	703,53	693,16	635	624,72		704,89	753,84	734,43	750,13	705,8	692,25	
	T2_1			436,79	500,89	523,21	469,48	418,58	423,35		502,95	420,77	460,85	466,51	455,92	463,18	
	T7_1			81,92	75,12	83	85,63	80,29	71,03		74,98	84,89	87,83	70,48	72,33	70,29	
Sigma = 7	T1_1			522,97	612,48	674,58	657,4	596,48	624,72		659,02	730,79	707,51	703,57	659,39	692,25	
	T2_1			411,86	473,82	495,37	440,92	386,69	423,35		469,09	404,29	440,92	442,96	430,67	463,18	
	T7_1			75,81	71,77	77,45	80,89	75,13	71,03		69,89	83,15	81,89	65,86	66,5	70,29	
Sigma = 8	T1_1			497,55	585,1	645,12	622,26	558,16	624,72		614,05	706,8	680,8	660,01	616,51	692,25	
	T2_1			389,55	447,45	467,23	412,19	356,72	423,35		435,44	389,7	422,6	419,26	407,52	463,18	
	T7_1			70,03	68,29	72,34	76,01	70,04	71,03		65,51	81,57	76,54	61,66	61,27	70,29	
Sigma = 9	T1_1			472,85	559,36	615,46	588,23	521,11	624,72		571,18	681,48	654,32	619,8	577,07	692,25	
	T2_1			369,46	422,13	439,35	384,07	329	423,35		403,18	376,34	405,88	396,41	386,19	463,18	
	T7_1			64,69	64,79	67,69	71,24	65,18	71,03		61,74	80,01	71,85	57,82	56,64	70,29	
Sigma = 12	T1_1			404,88	486,92	527,2	493,99	422,34	624,72		460,63	598,12	576,83	518,47	475,81	692,25	
	T2_1			317,83	353,05	360,64	307,79	259,43	423,35		320,62	338,63	362,38	336,99	329,68	463,18	
	T7_1			51,55	54,84	56,14	58,44	52,62	71,03		53,15	74,83	61,16	48,13	45,85	70,29	

Table S18 shows the activity concentrations measured using WKP and with 0.6 mL small VOI methods on SPECTs images post-filtered with Gaussian filter (0-12 mm) for patient number 18. Whereas, RC represented patient specific recovery coefficient of right and the left kidneys, respectively.

Post-filtering (mm)	Data	Time p.i. (h)	Inj Act (MBq)	Right Kidney							Left Kidney						
			7252	VOI_1 (counts/mL)	VOI_2 (counts/mL)	VOI_3 (counts/mL)	VOI_4 (counts/mL)	VOI_5 (counts/mL)	WKP (counts/mL)	RC	VOI_1 (counts/mL)	VOI_2 (counts/mL)	VOI_3 (counts/mL)	VOI_4 (counts/mL)	VOI_5 (counts/mL)	WKP (counts/mL)	RC
sigma = 0	T1_1	22,7		992,2	1122,88	1170,97	1131,01	946,44	780,59	0,85	1068,38	1011,81	1032,24	1163,14	1028,43	776,11	0,84
	T2_1	50,8		627,78	709,11	763,03	726,97	639,02	522,33		740,71	655,48	788,17	665,63	609,66	505,82	
	T7_1	172,2		142,74	150,23	142,88	120,42	151,19	93,34		175,29	170,69	128,47	126,69	127,85	109,71	
Sigma = 3	T1_1			928,19	1057,69	1092,46	1063,42	927,59	780,59		1009,83	981,1	978,57	1070,42	937,45	776,11	
	T2_1			603,07	658,21	697,48	667	607,49	522,33		691,58	629,87	719,27	619,87	576,28	505,82	
	T7_1			130,42	140,76	135,74	113,69	128,98	93,34		165,42	153,4	124,57	113,16	117,88	109,71	
sigma = 4	T1_1			886,82	1010,95	1041,06	1018,06	905,22	780,59		972,33	958,02	940,2	1010,77	882,64	776,11	
	T2_1			585,83	627,09	658,9	631,5	582,9	522,33		658,49	610,85	673,84	588,12	552,09	505,82	
	T7_1			123,33	135,08	130,7	109,34	117,67	93,34		158,35	143,93	120,19	105,66	111,97	109,71	
Sigma = 5	T1_1			843,3	957,98	985,44	967,04	873,61	780,59		932,27	929,37	897,24	948,44	826,56	776,11	
	T2_1			566,8	594,55	619,97	595,46	553,85	522,33		622,52	588,1	625,94	552,96	523,37	505,82	
	T7_1			116,37	128,94	125,02	104,43	107,43	93,34		150,34	134,58	116,16	98,36	105,57	109,71	
Sigma = 6	T1_1			800,77	902,6	928,25	912,91	835,07	780,59		892,01	896,26	852,5	887,33	771,86	776,11	
	T2_1			547,54	561,91	581,97	560,09	522,65	522,33		586,31	562,68	579,42	516,76	491,4	505,82	
	T7_1			109,86	122,48	118,91	99,06	98,32	93,34		142	125,44	110,57	91,5	98,74	109,71	
Sigma = 7	T1_1			761,27	847,75	871,73	858,04	792,35	780,59		853,42	860,2	807,86	829,6	720,31	776,11	
	T2_1			529,25	530,32	545,7	526,24	491,13	522,33		551,79	535,77	536,5	481,51	457,75	505,82	
	T7_1			104	115,85	112,51	93,4	90,34	93,34		133,76	116,69	104,54	85,18	91,68	109,71	
Sigma = 8	T1_1			725,68	795,37	817,34	804,24	747,94	780,59		817,33	822,7	764,34	776,01	672,64	776,11	
	T2_1			512,49	500,64	511,51	494,26	460,52	522,33		520	508,45	497,94	448,43	423,91	505,82	
	T7_1			98,81	109,21	105,96	87,67	83,37	93,34		125,91	108,46	98,39	79,39	84,67	109,71	
Sigma = 9	T1_1			694,01	746,61	765,87	752,72	703,72	780,59		783,69	785,01	722,42	726,56	628,95	776,11	
	T2_1			497,22	473,39	479,58	464,23	431,52	522,33		491,33	481,59	463,68	418,02	391,08	505,82	
	T7_1			94,21	102,72	99,39	82,02	77,26	93,34		118,55	100,86	92,36	74,08	77,94	109,71	
Sigma = 12	T1_1			616,69	624,56	631,52	616,61	582,17	780,59		692,15	677,39	608,56	599,95	518,75	776,11	
	T2_1			455,99	406,72	397,81	385,25	356,33	522,33		422,07	408,57	381,63	342,14	305,32	505,82	
	T7_1			83,22	85,14	80,96	66,73	62,66	93,34		99,5	82,03	76,03	60,57	60,72	109,71	

Table S19 shows the activity concentrations measured using WKP and with 2 mL small VOI methods on SPECTs images post-filtered with Gaussian filter (0-12 mm) for patient number 1. Whereas, RC represented patient specific recovery coefficient of right and the left kidneys, respectively.

Post-filtering (mm)	Data	Time p.i. (h)	Inj Act (MBq)	Right Kidney							Left Kidney						
			7672	VOI_1 (counts/mL)	VOI_2 (counts/mL)	VOI_3 (counts/mL)	VOI_4 (counts/mL)	VOI_5 (counts/mL)	WKP (counts/mL)	RC	VOI_1 (counts/mL)	VOI_2 (counts/mL)	VOI_3 (counts/mL)	VOI_4 (counts/mL)	VOI_5 (counts/mL)	WKP (counts/mL)	RC
sigma = 0	T1_1	21,0		787,18	888,54	863,83	748,22	860,52	643,51	0,89	987,89	847,29	788,69	860,77	832,87	593,48	0,86
	T2_1	48,8		536,68	624,33	541,12	520,69	611,95	447,5		616,12	698,48	594,4	506,46	537,05	405,35	
	T7_1	170,4		118,36	127,63	117,65	99,91	109,83	76,42		110,77	139,48	116,65	104,35	123,34	65,31	
Sigma = 3	T1_1			741,93	856,08	818,28	723,54	801,85	643,51		915,48	781,72	730,38	822,53	768,13	593,48	
	T2_1			508,84	590,38	516,65	499,8	579,27	447,5		572,61	647,79	567,57	480,27	489,52	405,35	
	T7_1			108,07	116,37	110,41	97,65	102,7	76,42		103,21	129,51	110,32	94,02	112,84	65,31	
sigma = 4	T1_1			711,26	829,9	785,41	705,28	763,76	643,51		866,07	737,94	693,47	795,45	728,88	593,48	
	T2_1			489,2	565,55	498,67	485,68	557,44	447,5		542,94	613,46	546,76	462,41	458,55	405,35	
	T7_1			101,69	109,82	105,8	95,9	97,99	76,42		98,29	122,6	105,72	87,66	106,12	65,31	
Sigma = 5	T1_1			676,84	797,22	748,09	683,93	722,8	643,51		811,11	690,62	653,85	763,72	687,77	593,48	
	T2_1			466,43	536,55	478,35	469,96	533,33	447,5		510,13	575,35	521,35	442,33	426,05	405,35	
	T7_1			95,14	103,39	100,9	93,68	92,8	76,42		92,96	115,39	100,45	81,15	99,04	65,31	
Sigma = 6	T1_1			640,37	759,58	708,98	660,57	681,65	643,51		753,92	642,92	613,33	728,28	646,32	593,48	
	T2_1			441,65	505,15	457,21	453,42	508,21	447,5		476,23	535,76	492,67	420,92	394,57	405,35	
	T7_1			88,82	97,39	95,98	90,98	87,42	76,42		87,54	107,34	94,82	74,89	92,8	65,31	
Sigma = 7	T1_1			603,28	718,7	669,93	635,97	642,37	643,51		697,37	596,95	573,53	690,2	605,87	593,48	
	T2_1			415,93	473,03	436,24	436,56	483,24	447,5		442,95	496,56	462,29	398,93	365,63	405,35	
	T7_1			82,89	91,96	91,23	87,89	82,06	76,42		82,29	99,4	89,06	69,14	85,5	65,31	
Sigma = 8	T1_1			566,65	676,22	632	610,73	606,09	643,51		643,44	553,83	535,55	650,49	567,19	593,48	
	T2_1			390,14	441,45	415,89	419,66	459,17	447,5		411,34	459,04	431,63	377,01	339,75	405,35	
	T7_1			77,39	87,07	86,78	84,52	76,89	76,42		77,33	91,61	83,34	63,97	79,42	65,31	
Sigma = 9	T1_1			531,19	633,55	595,72	585,28	573,25	643,51		593,33	514,08	499,99	610,16	530,57	593,48	
	T2_1			364,94	411,2	396,34	402,89	436,43	447,5		381,96	423,94	401,81	355,67	316,92	405,35	
	T7_1			72,28	82,64	82,66	80,99	72,03	76,42		72,78	84,28	77,76	59,36	73,82	65,31	
Sigma = 12	T1_1			435,58	513,78	497,94	510,57	494	643,51		414,82	408,88	494,54	433,51	410,36	593,48	
	T2_1			296,86	331,9	342,25	354,26	376,83	447,5		307,95	335,86	323,93	298,91	262,8	405,35	
	T7_1			58,89	71,33	72,14	70,54	59,95	76,42		61,79	66,19	62,63	48,32	59,59	65,31	

Table S20 shows the activity concentrations measured using WKP and with 2 mL small VOI methods on SPECTs images post-filtered with Gaussian filter (0-12 mm) for patient number 2. Whereas, RC represented patient specific recovery coefficient of right and the left kidneys, respectively.

Post-filtering (mm)	Data	Time p.i. (h)	Inj Act (MBq)	Right Kidney								Left Kidney					
			7692	VOI_1 (counts/mL)	VOI_2 (counts/mL)	VOI_3 (counts/mL)	VOI_4 (counts/mL)	VOI_5 (counts/mL)	WKP (counts/mL)	RC	VOI_1 (counts/mL)	VOI_2 (counts/mL)	VOI_3 (counts/mL)	VOI_4 (counts/mL)	VOI_5 (counts/mL)	WKP (counts/mL)	RC
sigma = 0	T1_1	22,8		812,77	834	699,31	728,89	862,67	637,65		797,87	913,9	819,38	826,53	959,09	661,07	
	T2_1	50,4		608,94	601,76	623,78	592,81	635,84	545,18		644,39	701,33	678,94	572,37	716,99	523,75	
	T7_1	171,3		113,52	96,01	104,04	127,54	125,37	103,62		102,8	119,93	133,22	126,16	121,58	105,79	
Sigma = 3	T1_1			765,55	786,81	676,1	700,53	809,78	637,65		745,75	842,78	772,84	803,65	922,6	661,07	
	T2_1			566,75	554,66	580,33	571,45	605,21	545,18		610,93	653,02	631,15	544,33	686,31	523,75	
	T7_1			103,21	93,15	99,37	117,23	117,65	103,62		95,8	112,37	125,39	121,2	118,4	105,79	
sigma = 4	T1_1			732,61	753,78	659,37	682,2	776,12	637,65		712,22	797,72	741,61	784,85	892,32	661,07	
	T2_1			538,98	525,27	550,56	557,2	584,39	545,18		587,78	620,72	598,17	525,78	662,06	523,75	
	T7_1			96,7	91,18	96,22	111,31	112,84	103,62		91,38	107,39	120,11	117,65	115,81	105,79	
Sigma = 5	T1_1			695,15	715,89	639,42	662,7	740,25	637,65		675,91	749,4	706,4	761,27	858,89	661,07	
	T2_1			508,9	494,11	517,33	541,67	560,98	545,18		561,1	584,97	561	505,33	632,43	523,75	
	T7_1			89,99	88,87	92,87	105,5	107,67	103,62		86,68	103,18	114,23	113,36	112,55	105,79	
Sigma = 6	T1_1			655,58	675,33	616,87	642,91	704,28	637,65		638,63	700,2	668,65	734,32	812,82	661,07	
	T2_1			478,26	462,58	482,74	523,36	536,15	545,18		531,96	547,57	521,92	484,04	599,24	523,75	
	T7_1			83,58	86,22	89,57	100,06	102,34	103,62		81,95	98,08	108,03	108,51	108,79	105,79	
Sigma = 7	T1_1			616,06	634,16	592,33	622,93	669,58	637,65		602,05	652,3	630,08	705,37	768,57	661,07	
	T2_1			448,35	431,76	448,61	504,24	510,88	545,18		501,68	510,1	483,05	462,77	564,46	523,75	
	T7_1			77,73	83,28	86,43	95,08	97,03	103,62		77,41	92,71	101,78	103,39	104,63	105,79	
Sigma = 8	T1_1			578,02	593,86	566,41	602,44	636,75	637,65		567,26	607,1	591,97	675,58	724,2	661,07	
	T2_1			419,95	402,33	416,16	483,83	485,83	545,18		471,36	473,71	445,84	442,07	529,77	523,75	
	T7_1			72,54	80,11	83,45	90,54	91,85	103,62		73,16	87,2	95,63	98,2	100,18	105,79	
Sigma = 9	T1_1			542,27	555,4	539,68	581,15	605,84	637,65		534,82	565,32	555,26	645,77	681,21	661,07	
	T2_1			393,41	374,68	386,02	462,38	461,46	545,18		441,83	439,16	411,19	422,23	496,36	523,75	
	T7_1			67,98	76,79	80,56	86,37	86,89	103,62		69,23	81,68	89,68	93,08	95,57	105,79	
Sigma = 12	T1_1			450,57	454,92	459,93	512,3	522,59	637,65		452,48	461,51	458,15	561,04	567,4	661,07	
	T2_1			325,65	303,72	310,5	395,47	394,25	545,18		362,17	349,94	325,15	368,57	409,15	523,75	
	T7_1			57,23	66,64	71,78	75,31	73,78	103,62		59,06	66,26	73,48	78,57	81,66	105,79	

Table S21 shows the activity concentrations measured using WKP and with 2 mL small VOI methods on SPECTs images post-filtered with Gaussian filter (0-12 mm) for patient number 3. Whereas, RC represented patient specific recovery coefficient of right and the left kidneys, respectively.

Post-filtering (mm)	Data	Time p.i. (h)	Inj Act (MBq)	Right Kidney							Left Kidney						
			7670	VOI_1 (counts/mL)	VOI_2 (counts/mL)	VOI_3 (counts/mL)	VOI_4 (counts/mL)	VOI_5 (counts/mL)	WKP (counts/mL)	RC	VOI_1 (counts/mL)	VOI_2 (counts/mL)	VOI_3 (counts/mL)	VOI_4 (counts/mL)	VOI_5 (counts/mL)	WKP (counts/mL)	RC
sigma = 0	T1_1	23,0		1165,82	1711,62	1372,07	1108,85	1050,36	903,77	0,81	1492,55	1672,98	1788,99	1566,18	1785,49	1233,85	0,90
	T2_1	50,2		979,14	1121,6	1095,33	1007,18	807,75	683,92		1158,89	1095,96	1115,95	1042,36	1196,39	864,09	
	T7_1	169,5		262,17	340,5	376,89	274,15	244,39	207,99		232,49	260,84	254,05	297,24	277,09	193,94	
Sigma = 3	T1_1			1095,58	1518,21	1249,36	1031,69	949,45	903,77		1399,16	1575,21	1685,36	1444,19	1707,47	1233,85	
	T2_1			894,13	1015,64	995,21	900,52	736,77	683,92		1078,09	1061,43	1053,67	997,3	1152,91	864,09	
	T7_1			240,89	305,69	331,55	244,65	221,7	207,99		215,77	243,89	246,44	283,12	267,35	193,94	
sigma = 4	T1_1			1048,37	1399,54	1171,74	979,85	886,46	903,77		1336,37	1509,71	1613,02	1402,95	1652,9	1233,85	
	T2_1			843,5	947,89	929,7	835,73	689,39	683,92		1025,02	1032,83	1010,3	968,57	1122,34	864,09	
	T7_1			228,05	283,49	302,21	227,29	207,26	207,99		205,33	233,09	239,95	273,57	260,32	193,94	
Sigma = 5	T1_1			996,28	1277,94	1089,71	922,25	821,17	903,77		1262,72	1436,87	1530,98	1357,39	1590,18	1233,85	
	T2_1			791,48	876,57	859,58	769,58	638,13	683,92		967,67	996,95	962,2	937,84	1087,13	864,09	
	T7_1			214,79	260,18	274,29	209,73	191,89	207,99		194,48	221,68	231,63	263,03	252,09	193,94	
Sigma = 6	T1_1			942,2	1160,84	1007,65	861,84	757,68	903,77		1197,59	1360,25	1443,99	1309,86	1522,85	1233,85	
	T2_1			740,25	806,37	789,43	705,88	586,49	683,92		909,62	955,77	912,63	906,78	1048,82	864,09	
	T7_1			201,9	237,33	246,87	192,78	176,41	207,99		183,89	210,3	221,98	252,18	243,04	193,94	
Sigma = 7	T1_1			888,49	1053,05	928,95	801,23	698,63	903,77		1129,41	1282,88	1356,34	1262,33	1454,25	1233,85	
	T2_1			691,72	740,46	722,66	646,95	537,02	683,92		853,63	911,13	863,78	876,57	1008,91	864,09	
	T7_1			189,53	216,13	222,06	177,03	161,51	207,99		174,03	199,31	211,65	241,47	233,49	193,94	
Sigma = 8	T1_1			836,59	956,52	855,51	742,22	645,11	903,77		1065,37	1206,91	1271,11	1215,97	1386,91	1233,85	
	T2_1			646,8	680,39	661,13	593,56	491,12	683,92		801,31	864,75	816,89	847,79	968,57	864,09	
	T7_1			178,28	197,19	200,23	162,7	147,58	207,99		165,13	188,87	201,2	231,15	223,74	193,94	
Sigma = 9	T1_1			787,27	871,41	788,05	686,02	597,3	903,77		1006,65	1133,78	1190,27	1171,27	1322,44	1233,85	
	T2_1			605,7	626,63	605,6	545,58	449,37	683,92		753,4	818,08	772,64	820,57	928,7	864,09	
	T7_1			168,13	180,68	181,3	149,8	134,84	207,99		157,29	179,01	191,06	221,38	214,03	193,94	
Sigma = 12	T1_1			656,31	647,85	620,51	539,79	484,09	903,77		863,63	938,93	981,48	1047,07	1152,03	1233,85	
	T2_1			502,44	499,68	473,57	428,88	348,49	683,92		635,66	686,67	657,55	746,23	817,23	864,09	
	T7_1			143,31	143,96	139,01	118,53	103,78	207,99		139,48	152,91	164,43	195,6	186,79	193,94	

Table S22 shows the activity concentrations measured using WKP and with 2 mL small VOI methods on SPECTs images post-filtered with Gaussian filter (0-12 mm) for patient number 4. Whereas, RC represented patient specific recovery coefficient of right and the left kidneys, respectively.

Post-filtering (mm)	Data	Time p.i. (h)	Inj Act (MBq)	Right Kidney							Left Kidney						
			7777	VOI_1 (counts/mL)	VOI_2 (counts/mL)	VOI_3 (counts/mL)	VOI_4 (counts/mL)	VOI_5 (counts/mL)	WKP (counts/mL)	RC	VOI_1 (counts/mL)	VOI_2 (counts/mL)	VOI_3 (counts/mL)	VOI_4 (counts/mL)	VOI_5 (counts/mL)	WKP (counts/mL)	RC
sigma = 0	T1_1	23,3		695,9	1008,7	1212,62	1185,18	1268,68	879,22	0,87	799,49	951,66	958,38	944,07	1084,12	829,16	0,86
	T2_1	51,1		514,51	674,73	700,73	710,34	745,39	479,36		656,08	567,95	608,28	654,11	660,21	528,34	
	T9_1	217,8		63,11	57,59	81,67	85,18	84,62	46,72		74,5	72,86	73,73	67,69	75,65	47,8	
Sigma = 3	T1_1			662,23	960,9	1141,31	1134,88	1196,71	879,22		746,72	888,38	907,65	894,53	1025,68	829,16	
	T2_1			492,77	641,1	660,74	668,85	689,68	479,36		590,13	534,91	572,31	625,21	621,49	528,34	
	T7_1			59,95	55,9	76,22	79,59	77,28	46,72		65,64	69,27	67,92	62,88	69,71	47,8	
sigma = 4	T1_1			642,59	925,07	1090,07	1097,22	1143,86	879,22		710,91	845,6	875,29	863,82	985,57	829,16	
	T2_1			478,26	616,52	631,76	639,52	654	479,36		549,55	512,48	549,87	604,2	594,91	528,34	
	T9_1			57,52	54,87	72,51	75,59	72,92	46,72		60,36	66,69	64,75	60,07	66,06	47,8	
Sigma = 5	T1_1			623,33	882,71	1031,53	1053,2	1083,96	879,22		672,01	798,77	840,26	832,31	941,16	829,16	
	T2_1			461,94	587,3	598,26	606,47	616,16	479,36		507,84	487,39	526,23	579,89	565,72	528,34	
	T9_1			54,73	53,74	68,47	71,04	68,4	46,72		55,22	63,78	61,91	57,26	62,23	47,8	
Sigma = 6	T1_1			605,2	836,33	969,57	1005,38	1021,65	879,22		632,86	750,9	803,95	802,08	895,55	829,16	
	T2_1			444,37	554,74	562,35	571,94	578,41	479,36		467,52	460,88	502,49	553,68	536,07	528,34	
	T9_1			51,84	52,52	64,37	66,28	63,9	46,72		50,59	60,75	59,57	54,61	58,41	47,8	
Sigma = 7	T1_1			588,28	787,99	907,3	995,79	960,12	879,22		595,57	704,3	767,63	774,29	851,2	829,16	
	T2_1			425,97	520,35	525,86	537,64	542,35	479,36		430,22	433,95	479,43	526,87	507,62	528,34	
	T9_1			49,05	51,23	60,42	60,59	59,54	46,72		46,65	57,76	57,79	52,27	54,76	47,8	
Sigma = 8	T1_1			572,12	739,37	846,82	905,83	901,33	879,22		561,33	660,35	732,24	749,21	809,69	829,16	
	T2_1			407,04	485,46	490,09	504,57	508,79	479,36		396,58	407,32	457,35	500,41	481,42	528,34	
	T7_1			46,49	49,88	56,7	57,17	55,41	46,72		43,39	54,94	56,55	50,35	51,38	47,8	
Sigma = 9	T1_1			556,07	691,74	789,34	856,46	846,3	879,22		530,63	619,82	698,33	726,57	771,84	829,16	
	T2_1			387,81	451,15	455,81	473,25	478,01	479,36		366,64	381,51	436,24	474,92	458,03	528,34	
	T7_1			44,21	48,48	53,27	53,12	51,53	46,72		52,33	55,81	48,91	48,35	42,62	47,8	
Sigma = 12	T1_1			504,56	562,47	638,88	717,34	705,72	879,22		458,41	519,81	608,03	668,2	680,28	829,16	
	T2_1			330,59	358,21	365,33	390,74	401,07	479,36		296,11	312,13	377,38	406,71	404,62	528,34	
	T7_1			38,92	44,23	44,76	43,24	41,41	46,72		35,29	45,97	55,97	47,34	51,51	47,8	

Table S23 shows the activity concentrations measured using WKP and with 2 mL small VOI methods on SPECTs images post-filtered with Gaussian filter (0-12 mm) for patient number 5. Whereas, RC represented patient specific recovery coefficient of right and the left kidneys, respectively.

Post-filtering (mm)	Data	Time p.i. (h)	Inj Act (MBq)	Right Kidney							Left Kidney						
			7726	VOI_1 (counts/mL)	VOI_2 (counts/mL)	VOI_3 (counts/mL)	VOI_4 (counts/mL)	VOI_5 (counts/mL)	WKP (counts/mL)	RC	VOI_1 (counts/mL)	VOI_2 (counts/mL)	VOI_3 (counts/mL)	VOI_4 (counts/mL)	VOI_5 (counts/mL)	WKP (counts/mL)	RC
sigma = 0	T1_1	19,9		1128,45	1144,79	1193,45	1321,12	1336,99	1011,9	0,87	881,35	973,32	896,85	835,52	975,81	708,57	0,74
	T2_1	43,8		929,13	971,85	1005,15	1021,94	1055,93	846,1		766,32	835,33	893,69	912,35	864,94	599,16	
	T7_1	169,2		164,48	176,15	171,44	176,42	189,52	140,46		137,44	136,49	107,91	144,54	173,04	98,76	
Sigma = 3	T1_1			1060,08	1072,51	1138	1262,32	1273,91	1011,9		811,89	883,38	837,32	791,73	928,68	708,57	
	T2_1			859,45	923,33	957,54	960,89	1000,4	846,1		692,1	740,45	811,79	824,04	778,7	599,16	
	T7_1			156,34	169,11	167,61	170,26	178,04	140,46		123,31	130,12	100,14	131,37	146,47	98,76	
sigma = 4	T1_1			1012,27	1025,56	1099,56	1221,01	1230,08	1011,9		765,74	824,6	795,44	760,04	891,79	708,57	
	T2_1			812,41	889,66	925,63	922,02	963,17	846,1		644,13	681,18	758,64	765,32	721,27	599,16	
	T7_1			150,27	163,56	164,22	166,16	171,46	140,46		114,98	125,53	95,23	123,33	130,58	98,76	
Sigma = 5	T1_1			959,95	974,2	1055,57	1174,37	1180,88	1011,9		714,93	760,8	747,32	723,98	847,53	708,57	
	T2_1			760,44	850,7	889,95	880,66	922,45	846,1		592,39	619,73	701,66	702,06	659,7	599,16	
	T7_1			134,01	156,7	159,76	161,49	164,74	140,46		106,72	120,14	89,99	115,01	114,92	98,76	
Sigma = 6	T1_1			900,34	920,73	1008,26	1125,01	1129,24	1011,9		662,23	695,84	695,56	686,15	798,9	708,57	
	T2_1			706,98	807,98	852,1	838,96	880,69	846,1		539,96	559,87	644,28	638,73	598,51	599,16	
	T7_1			134,94	148,95	154,36	156,29	157,99	140,46		99,03	114,26	84,7	106,91	100,59	98,76	
Sigma = 7	T1_1			842,25	867,21	959,76	1074,78	1077,33	1011,9		609,98	632,91	642,83	648,27	748,47	708,57	
	T2_1			654,86	763,19	813,36	798,28	839,62	846,1		489,45	504,09	589,05	578,52	540,72	599,16	
	T7_1			126,49	140,75	148,23	150,61	151,34	140,46		92,21	108,24	79,59	99,36	88,1	98,76	
Sigma = 8	T1_1			785,7	815,04	911,52	1024,81	1026,44	1011,9		559,82	574,07	591,32	611,36	698,24	708,57	
	T2_1			605,77	717,85	774,53	759,17	800,08	846,1		442,53	453,57	537,51	523,21	487,97	599,16	
	T7_1			118,05	132,49	141,59	144,56	144,84	140,46		86,35	102,37	74,79	92,51	77,52	98,76	
Sigma = 9	T1_1			732,03	765,09	864,41	975,73	977,25	1011,9		512,83	520,43	542,61	576,03	649,73	708,57	
	T2_1			560,55	673,17	736,02	721,74	762,3	846,1		400,01	408,69	490,48	473,61	440,94	599,16	
	T7_1			109,89	124,4	134,69	138,26	138,53	140,46		81,41	96,83	70,37	86,41	68,68	98,76	
Sigma = 12	T1_1			593,92	632,29	733,05	836,18	841,25	1011,9		395,64	392,93	420,97	481,83	522,34	708,57	
	T2_1			449,12	550,8	624,78	618,72	658,14	846,1		299,76	306,03	377,8	358,78	333,26	599,16	
	T7_1			88,67	102,46	114,14	119,17	120,61	140,46		70,86	82,82	59,39	72,17	50,33	98,76	

Table S24 shows the activity concentrations measured using WKP and with 2 mL small VOI methods on SPECTs images post-filtered with Gaussian filter (0-12 mm) for patient number 6. Whereas, RC represented patient specific recovery coefficient of right and the left kidneys, respectively.

Post-filtering (mm)	Data	Time p.i. (h)	Inj Act (MBq)	Right Kidney							Left Kidney						
			7723	VOI_1 (counts/mL)	VOI_2 (counts/mL)	VOI_3 (counts/mL)	VOI_4 (counts/mL)	VOI_5 (counts/mL)	WKP (counts/mL)	RC	VOI_1 (counts/mL)	VOI_2 (counts/mL)	VOI_3 (counts/mL)	VOI_4 (counts/mL)	VOI_5 (counts/mL)	WKP (counts/mL)	RC
sigma = 0	T1_1	23,2		849,2	872,39	946,01	1095,86	1020,31	742,34	0,85	950,97	889,83	1054,29	1000,35	961,82	732,77	0,88
	T2_1	47,3		621,51	670,92	796,13	770,05	718,57	554,34		631,06	655,93	612,38	710,81	791,45	547,84	
	T7_1	170,7		172,09	149,84	167,75	169,07	159,76	134,56		165,2	187,01	151,3	155,72	153,19	132,64	
Sigma = 3	T1_1			804,45	806,68	909,94	1037,68	986,8	742,3		897,8	823,2	1017,2	945,1	899,3	732,8	
	T2_1			586,85	638,42	760,43	748,25	692,82	554,3		575,2	608,7	592,8	679,8	743,8	547,8	
	T7_1			159,82	140,64	161,75	163,73	153,63	134,6		150,6	172,7	148,5	152,5	152,7	132,6	
sigma = 4	T1_1			772,66	763,11	882,85	996,99	961,82	742,34		860,3	780,58	990,09	908,22	858,89	732,77	
	T2_1			562,45	615,06	735,37	729,82	675,8	554,34		541,82	578,39	579,76	658,98	713,34	547,84	
	T7_1			151,46	134,76	156,94	159,93	149,56	134,56		141,87	163,53	146,44	150,37	151,9	132,64	
Sigma = 5	T1_1			735,75	715,22	850,25	951,44	931,86	742,34		818,1	735,54	960,04	868,17	815,73	732,77	
	T2_1			534,55	588,34	706,08	706,1	657,05	554,34		507,97	545,99	565,13	635,8	680,87	547,84	
	T7_1			142,2	128,46	150,96	155,37	145,14	134,56		133,02	153,8	144,05	148,08	150,59	132,64	
Sigma = 6	T1_1			695,47	665,87	813,45	903,79	898,32	742,34		773,85	691,09	926,17	824,12	772	732,77	
	T2_1			504,54	559,74	673,54	677,95	636,99	554,34		475,23	513,23	549,61	611,41	648,32	547,84	
	T7_1			132,61	122,06	144,12	150,14	140,64	134,56		124,53	144,18	141,4	145,67	148,72	132,64	
Sigma = 7	T1_1			653,49	617,43	773,75	855,88	862,37	742,34		729,63	649,22	890,39	789,58	729,35	732,77	
	T2_1			473,9	530,44	639,04	646,81	615,63	554,34		444,87	481,53	533,85	586,85	616,9	547,84	
	T7_1			123,17	115,81	136,83	144,42	139,15	134,56		116,73	135,13	138,47	143,14	146,27	132,64	
Sigma = 8	T1_1			611,35	571,41	732,34	808,72	824,87	742,34		686,75	610,89	853,68	747,36	688,63	732,77	
	T2_1			443,73	501,22	603,78	614,08	592,86	554,34		417,44	451,74	518,19	562,85	587,15	547,84	
	T7_1			114,16	109,8	129,43	138,39	131,7	134,56		109,72	126,86	135,24	140,46	143,3	132,64	
Sigma = 9	T1_1			570,24	528,63	690,24	762,88	786,52	742,34		645,99	576,3	816,88	709,88	650,18	732,77	
	T2_1			414,79	472,6	568,69	580,92	568,71	554,34		392,97	424,24	502,72	539,84	559,28	547,84	
	T7_1			105,75	104,07	122,19	132,2	127,23	134,56		103,51	119,43	131,72	137,56	139,9	132,64	
Sigma = 12	T1_1			460,17	421,34	568,11	635,92	671,71	742,34		539	492,01	710,74	608,87	547,99	732,77	
	T2_1			338,22	392,93	470,14	485,84	491,05	554,34		334,64	355,49	456,44	477,38	486,15	547,84	
	T7_1			84,61	88,4	102,61	113,85	113,39	134,56		88,95	101,41	119,72	127,46	128,17	132,64	

Table S25 shows the activity concentrations measured using WKP and with 2 mL small VOI methods on SPECTs images post-filtered with Gaussian filter (0-12 mm) for patient number 7. Whereas, RC represented patient specific recovery coefficient of right and the left kidneys, respectively.

Post-filtering (mm)	Data	Time p.i. (h)	Inj Act (MBq)	Right Kidney									Left Kidney				
			7713	VOI_1 (counts/mL)	VOI_2 (counts/mL)	VOI_3 (counts/mL)	VOI_4 (counts/mL)	VOI_5 (counts/mL)	WKP (counts/mL)	RC	VOI_1 (counts/mL)	VOI_2 (counts/mL)	VOI_3 (counts/mL)	VOI_4 (counts/mL)	VOI_5 (counts/mL)	WKP (counts/mL)	RC
sigma = 0	T1_1	22,3		900,4	1090,39	1091,44	1043,36	1008,16	815,15	0,86	812,55	974,09	935,94	912,27	992,28	755,35	0,86
	T2_1	48,1		601,83	700,58	634,09	533,38	574,41	536,03		703,81	761,96	710,34	648,37	638,38	489,57	
	T7_1	170,2		137,44	132,59	106,52	133,07	127,01	108,46		165,23	151,18	139,77	161,65	119	100,09	
Sigma = 3	T1_1			849,72	1014,28	1022,98	990,49	954,98	815,2		778,9	917,3	880,1	867,7	947,7	755,35	
	T2_1			562,06	647,26	587,53	508,09	542,48	536,0		653,0	713,5	679,1	627,7	607,9	489,57	
	T7_1			131,82	127,08	106,31	129,03	125,1	108,5		149,3	145,6	136,5	152,1	116,4	100,09	
sigma = 4	T1_1			813,5	961,88	975,21	951,12	920,18	815,15		756,85	879,01	840,58	835,93	917,85	755,35	
	T2_1			534,59	611,5	557,12	491,06	522,13	536,03		619,74	679,72	656,9	613	587,53	489,57	
	T7_1			127,29	123,57	105,74	125,67	123	108,46		140,05	141,43	133,34	145,17	114	100,09	
Sigma = 5	T1_1			771,82	903,54	921,53	905,49	881,84	815,15		733,08	836,54	795,53	799,23	884,39	755,35	
	T2_1			503,8	572,18	524,3	472,03	500,61	536,03		583,77	641,41	630,73	595,79	564,96	489,57	
	T7_1			121,96	119,84	104,93	121,67	120,04	108,46		130,77	136,41	129,25	137,54	110,73	100,09	
Sigma = 6	T1_1			727,16	842,89	865,26	856,72	841,69	815,15		709,42	792,37	747,66	759,4	848,52	755,35	
	T2_1			471,55	531,65	490,92	451,93	479,11	536,03		547,21	600,78	601,65	576,47	541,45	489,57	
	T7_1			116,27	116,1	103,99	117,41	116,37	108,46		121,88	130,79	124,63	129,84	106,77	100,09	
Sigma = 7	T1_1			681,67	782,8	809	807,28	801,17	815,15		687,35	748,78	699,68	718,29	811,41	755,35	
	T2_1			439,49	491,79	458,32	431,51	458,33	536,03		511,73	560	570,86	555,49	517,97	489,57	
	T7_1			110,56	112,39	102,92	113,08	112,11	108,46		113,71	124,88	119,72	122,44	102,32	100,09	
Sigma = 8	T1_1			636,9	725,09	754,44	758,85	761,26	815,15		667,69	707,45	653,64	677,48	774,02	755,35	
	T2_1			408,74	453,8	427,22	411,26	438,53	536,03		478,38	520,68	539,53	533,31	495,13	489,57	
	T7_1			105,09	108,73	101,65	108,82	107,43	108,46		106,37	118,93	114,75	115,53	97,57	100,09	
Sigma = 9	T1_1			593,89	670,77	702,57	712,4	722,51	815,15		650,58	669,48	610,96	638,25	737,15	755,35	
	T2_1			379,92	418,35	397,98	391,43	419,72	536,03		447,78	483,93	508,66	510,44	473,23	489,57	
	T7_1			100,05	105,15	100,13	104,65	102,49	108,46		99,92	113,15	109,88	109,16	92,73	100,09	
Sigma = 12	T1_1			480,39	531,66	566,91	588,66	615,7	815,15		609,3	578,7	509,1	538,07	636,1	755,35	
	T2_1			306,9	329,25	322,3	335,48	367,88	536,03		374,62	393,51	425,78	442,96	414	489,57	
	T7_1			88,55	95,55	93,91	92,89	87,61	108,46		85,92	98,18	96,86	93,51	79,06	109	

Table S26 shows the activity concentrations measured using WKP and with 2 mL small VOI methods on SPECTs images post-filtered with Gaussian filter (0-12 mm) for patient number 8. Whereas, RC represented patient specific recovery coefficient of right and the left kidneys, respectively.

Post-filtering (mm)	Data	Time p.i. (h)	Inj Act (MBq)	Right Kidney							Left Kidney						
			7690	VOI_1 (counts/mL)	VOI_2 (counts/mL)	VOI_3 (counts/mL)	VOI_4 (counts/mL)	VOI_5 (counts/mL)	WKP (counts/mL)	RC	VOI_1 (counts/mL)	VOI_2 (counts/mL)	VOI_3 (counts/mL)	VOI_4 (counts/mL)	VOI_5 (counts/mL)	WKP (counts/mL)	RC
sigma = 0	T1_1	19,5		629,88	774,03	889,3	865,46	812,28	691,31	0,87	765,56	984,27	964,99	839,8	884,36	758,2	0,88
	T2_1	46,7		502,35	558,53	602	660,92	593,51	512,72		585,21	683,22	663,44	615,67	747,77	541,35	
	T7_1	169,1		135,94	157,1	129,07	118,39	100,23	99,5		113,04	166,96	124,31	146,18	130,05	96,43	
Sigma = 3	T1_1			608,29	733,02	857,58	834,3	785,28	691,3		730,2	937,5	929,3	828,4	856,4	758,2	
	T2_1			477,85	529,55	582,41	620,5	575,53	512,7		544,8	641,1	635,8	593,3	697,0	541,4	
	T7_1			126,21	152,75	124,92	110,83	98,86	99,5		108,8	152,6	118,9	139,2	127,6	96,4	
sigma = 4	T1_1			595,1	702,76	832,29	812,16	767,17	691,31		706,29	905,62	902,23	819,5	838,22	758,2	
	T2_1			460,96	508,07	566,64	595,54	562,73	512,72		519,06	612,65	614,5	577,79	665,24	541,35	
	T7_1			119,89	150,11	122,47	106,46	97,3	99,5		105,47	143,54	115,52	134,42	124,63	96,43	
Sigma = 5	T1_1			581,09	667,35	801,28	786,5	747,39	691,31		679,76	869,16	869,35	807,69	817,97	758,2	
	T2_1			442,38	482,78	546,83	569,23	547,87	512,72		491,58	580,93	588,48	560,52	631,94	541,35	
	T7_1			113,2	147,31	120,17	102,1	94,81	99,5		101,53	134,14	111,88	128,88	120,49	96,43	
Sigma = 6	T1_1			566,52	628,98	766,15	758,5	727,19	691,31		654,95	829,51	831,96	792,67	796,33	758,2	
	T2_1			423,16	455,17	523,81	542,81	531,63	512,72		463,85	547,54	559,08	542,66	599,02	541,35	
	T7_1			106,63	144,47	118,27	97,91	91,43	99,5		97,17	124,92	108,18	122,95	115,48	96,43	
Sigma = 7	T1_1			551,26	589,66	782,41	729,12	707,17	691,31		624,02	788,12	791,64	774,51	774,08	758,2	
	T2_1			403,73	426,66	498,7	517,13	514,78	512,72		437,12	514,01	527,9	525,14	567,87	541,35	
	T7_1			100,5	141,7	116,89	93,96	87,37	99,5		92,64	116,21	104,6	116,99	109,96	96,43	
Sigma = 8	T1_1			534,98	550,91	689,36	699,08	687,41	691,31		596,73	746,2	749,89	753,54	751,73	758,2	
	T2_1			384,24	398,33	472,49	492,57	497,88	512,72		412,13	481,45	496,43	508,51	539,14	541,35	
	T7_1			95	139,04	115,84	90,26	82,89	99,5		113,04	152,71	82,32	86,15	103,94	96,43	
Sigma = 9	T1_1			517,4	513,74	650,03	668,84	667,66	691,31		570,54	704,76	708	730,21	729,47	758,2	
	T2_1			364,74	370,93	446,03	469,16	481,23	512,72		389,2	450,58	465,78	492,89	513,04	541,35	
	T7_1			90,2	136,48	115,05	86,78	78,25	99,5		83,79	100,76	98,11	105,95	98,68	96,43	
Sigma = 12	T1_1			457,39	416,15	537,59	579,69	606,16	691,31		499,89	589,66	590,33	651,48	662,46	758,2	
	T2_1			307,61	298,04	370,48	405,07	433,25	512,72		332,49	371,09	384,59	449,95	448,6	541,35	
	T7_1			79,73	129,17	112,58	77,45	65,14	99,5		72,38	82,55	89,83	92,43	83,71	96,43	

Table S27 shows the activity concentrations measured using WKP and with 2 mL small VOI methods on SPECTs images post-filtered with Gaussian filter (0-12 mm) for patient number 9. Whereas, RC represented patient specific recovery coefficient of right and the left kidneys, respectively.

Post-filtering (mm)	Data	Time p.i. (h)	Inj Act (MBq)	Right Kidney							Left Kidney						
			7738	VOI_1 (counts/mL)	VOI_2 (counts/mL)	VOI_3 (counts/mL)	VOI_4 (counts/mL)	VOI_5 (counts/mL)	WKP (counts/mL)	RC	VOI_1 (counts/mL)	VOI_2 (counts/mL)	VOI_3 (counts/mL)	VOI_4 (counts/mL)	VOI_5 (counts/mL)	WKP (counts/mL)	RC
sigma = 0	T0_1	4,4		699,09	719,97	693,73	710,55	747,14	530,54	0,86	582,54	744,62	677,17	736,88	688,81	489,49	0,87
	T2_1	51,0		323,92	362,9	361,7	372,42	433,88	279,86		281,66	400,98	315,26	332,2	386,52	249,22	
	T7_1	171,3		85,25	88,23	68,03	80,71	73,05	55,15		56,6	76,43	68,02	67,99	92,84	52,24	
Sigma = 3	T1_1			650,31	689,62	653,57	697,23	732,45	530,5		551,1	699,5	643,9	683,9	652,8	489,5	
	T2_1			309,55	349,48	342,58	353,28	414,36	279,9		266,1	371,1	306,1	319,8	363,5	249,2	
	T7_1			77,3	83,49	65,19	75,38	71,05	55,2		53,6	71,6	64,0	66,9	85,3	52,2	
sigma = 4	T0_1			617,47	667,35	627,91	686,21	719,74	530,54		529,8	668,83	621,25	649,82	630	489,49	
	T2_1			300,13	339,64	330,15	341,08	395,65	279,86		255,72	351,27	299,12	310,81	348,83	249,22	
	T7_1			72,16	80,28	63,49	72,12	69,7	55,15		51,38	68,33	61,34	65,96	80,77	52,24	
Sigma = 5	T0_1			581,41	640,93	600,46	671,41	703,08	530,54		506,15	634,43	595,72	613,44	605,13	489,49	
	T2_1			289,44	327,84	316,75	328,02	375,65	279,86		244,37	329,58	290,64	300,28	332,92	249,22	
	T7_1			66,75	76,66	61,81	68,79	68,18	55,15		48,93	64,74	58,51	64,62	76,1	52,24	
Sigma = 6	T0_1			544,34	611,57	572,52	652,88	682,89	530,54		481,49	598,18	568,61	577,06	578,91	489,49	
	T2_1			277,76	314,39	302,98	314,65	355,48	279,86		232,58	307,43	281,02	288,78	316,39	249,22	
	T7_1			61,47	72,75	60,21	65,59	66,57	55,15		46,4	61,09	55,61	62,88	71,55	52,24	
Sigma = 7	T0_1			508,02	580,6	544,99	631,21	656,84	530,54		457,08	561,75	541,16	542,34	552,08	489,49	
	T2_1			265,35	299,81	289,3	301,42	339,04	279,86		220,92	285,92	270,6	276,82	299,79	249,22	
	T7_1			56,53	68,73	58,66	62,65	64,93	55,15		43,94	57,52	52,77	60,75	67,22	52,24	
Sigma = 8	T1_1			473,6	549,14	518,3	607,24	634,81	530,54		433,85	526,35	514,27	510,09	525,2	489,49	
	T2_1			252,49	284,61	275,97	288,61	317,87	279,86		209,8	265,8	259,69	264,84	283,53	249,22	
	T7_1			52,03	64,71	57,12	59,99	63,31	55,15		41,67	54,12	50,08	58,34	63,13	52,24	
Sigma = 9	T1_1			441,67	518,06	492,67	581,84	608,72	530,54		412,38	492,74	488,46	480,53	498,71	489,49	
	T2_1			239,44	269,29	263,18	276,37	301,22	279,86		199,53	247,49	248,55	253,1	267,9	249,22	
	T7_1			47,98	60,77	55,55	57,62	61,73	55,15		39,64	50,94	47,59	55,74	59,3	52,24	
Sigma = 12	T1_1			361,73	432,02	422,76	504,57	532,26	530,54		359,16	406,01	418,9	406,34	424,76	489,49	
	T2_1			201,76	225,88	228,83	243,62	260,88	279,86		175,07	204,22	215,77	220,67	226,26	249,22	
	T7_1			38,24	49,98	50,47	51,88	57,4	55,15		35,04	43,04	41,31	47,84	49,27	52,24	

Table S28 shows the activity concentrations measured using WKP and with 2 mL small VOI methods on SPECTs images post-filtered with Gaussian filter (0-12 mm) for patient number 10. Whereas, RC represented patient specific recovery coefficient of right and the left kidneys, respectively.

Post-filtering (mm)	Data	Time p.i. (h)	Inj Act (MBq)	Right Kidney									Left Kidney				
			7738	VOI_1 (counts/mL)	VOI_2 (counts/mL)	VOI_3 (counts/mL)	VOI_4 (counts/mL)	VOI_5 (counts/mL)	WKP (counts/mL)	RC	VOI_1 (counts/mL)	VOI_2 (counts/mL)	VOI_3 (counts/mL)	VOI_4 (counts/mL)	VOI_5 (counts/mL)	WKP (counts/mL)	RC
sigma = 0	T1_1	22,2		845,04	759,47	794,68	783,08	705,84	670,81	0,83	813,36	829,45	1038,26	1098,01	846,76	717,2	0,82
	T2_1	119,1		152,64	106,75	102,79	147,74	152,51	126,71		139,97	167,54	123,32	116,58	116,58	123,39	
	T7_1	173,2		79,1	67,65	63,86	66,87	62,46	58,98		57,92	52,52	52,19	51,22	43,45	60,76	
Sigma = 3	T1_1			784,75	716,88	745,14	708,44	664,86	670,8		766,6	797,4	987,5	1035,5	804,5	717,2	
	T2_1			139,2	106,24	100,54	139,92	145,28	126,7		127,8	154,7	118,0	112,1	110,1	123,4	
	T7_1			72,48	64,19	58,7	60,78	58,18	59,0		54,1	50,9	51,1	51,1	46,2	60,8	
sigma = 4	T1_1			745,06	689,93	709,79	663,28	635,75	670,81		735,96	772,37	953,2	995,14	774,64	717,2	
	T2_1			130,93	105,5	99,38	135,39	140,42	126,71		120,97	146,37	114,55	108,56	105,99	123,39	
	T7_1			68,34	61,9	55,33	57,05	55,35	58,98		51,48	49,82	50,24	50,58	47,05	60,76	
Sigma = 5	T1_1			702,84	661,25	669,99	616,66	602,89	670,81		702,38	742,39	915,59	950,9	741,47	717,2	
	T2_1			122,37	104,24	98,29	130,81	134,99	126,71		114,28	137,48	111,03	104,71	101,78	123,39	
	T7_1			64,07	59,28	51,82	53,32	52,38	58,98		48,78	48,57	49,18	49,64	47,23	60,76	
Sigma = 6	T1_1			661,33	632,16	628,56	570,92	568,68	670,81		667,45	709,3	876,96	904,75	707,44	717,2	
	T2_1			113,99	102,36	97,29	126,24	129,22	126,71		107,9	128,63	107,91	101,03	97,84	123,39	
	T7_1			60,02	56,46	48,44	49,86	49,5	58,98		46,19	47,19	47,94	48,39	46,82	60,76	
Sigma = 7	T1_1			622,5	603,36	587,55	527,87	535,06	670,81		632,45	674,72	838,62	858,64	674,21	717,2	
	T2_1			106,2	99,86	96,29	121,78	123,4	126,71		102	120,28	105,45	97,86	94,41	123,39	
	T7_1			56,33	53,53	45,32	46,76	46,82	58,98		43,86	45,74	46,59	46,93	45,91	60,76	
Sigma = 8	T1_1			587,15	575,09	548,21	488,4	503,21	670,81		598,21	639,91	801,19	813,91	642,72	717,2	
	T2_1			99,17	96,85	95,2	117,43	117,68	126,71		96,66	112,7	103,72	95,33	91,58	123,39	
	T7_1			53,05	50,56	42,5	4,01	44,37	58,98		41,83	44,29	45,16	45,35	44,62	60,76	
Sigma = 9	T1_1			555,37	547,41	511,19	452,79	473,71	670,81		565,22	605,75	764,86	771,34	613,31	717,2	
	T2_1			92,95	93,43	93,96	113,17	112,16	126,71		91,9	105,99	102,64	93,49	89,35	123,39	
	T7_1			50,15	47,61	39,97	41,58	42,17	58,98		40,11	42,85	43,68	43,71	43,11	60,76	
Sigma = 12	T1_1			477,09	468,4	415,75	367,15	399,82	670,81		475,76	511,68	662,49	658,72	536,91	717,2	
	T2_1			78,41	82,17	88,78	100,77	96,99	126,71		80,73	90,61	101,58	91	85,2	123,39	
	T7_1			43,15	39,54	33,74	35,73	36,8	58,98		36,41	38,81	39,24	38,85	38,17	60,76	

Table S29 shows the activity concentrations measured using WKP and with 2 mL small VOI methods on SPECTs images post-filtered with Gaussian filter (0-12 mm) for patient number 11. Whereas, RC represented patient specific recovery coefficient of right and the left kidneys, respectively.

Post-filtering (mm)	Data	Time p.i. (h)	Inj Act (MBq)	Right Kidney							Left Kidney						
			7736	VOI_1 (counts/mL)	VOI_2 (counts/mL)	VOI_3 (counts/mL)	VOI_4 (counts/mL)	VOI_5 (counts/mL)	WKP (counts/mL)	RC	VOI_1 (counts/mL)	VOI_2 (counts/mL)	VOI_3 (counts/mL)	VOI_4 (counts/mL)	VOI_5 (counts/mL)	WKP (counts/mL)	RC
sigma = 0	T1_1	22,7		695,26	769,93	684,98	735,12	832,93	609,22	0,87	605,37	629,62	663,6	688,87	682,36	578,46	0,86
	T5_1	119,7		154,75	160,1	168,18	172,66	195,81	120,09		141,49	178,45	150,53	186,57	163,54	120,57	
	T7_1	172,1		93,51	119,75	99,34	102,42	93,1	69,89		85,45	81,73	81,09	108,15	104,7	66,14	
Sigma = 3	T1_1			644,35	711,74	652,96	716,61	788,12	609,2		568,5	617,0	634,3	657,0	651,8	578,5	
	T2_1			143,35	152,25	158,97	163,18	184,61	120,1		134,9	167,4	149,1	170,6	153,2	120,6	
	T7_1			86,56	108,9	95,1	98,28	90,97	69,9		79,6	78,7	76,9	99,8	103,3	66,1	
sigma = 4	T1_1			611,31	673,71	630,3	701,6	760,65	609,22		542,78	604,22	613,79	635,98	631,23	578,46	
	T5_1			136,48	146,6	152,96	157,88	177,37	120,09		130,11	159,97	146,52	161,35	146,71	120,57	
	T7_1			82,3	102,23	92,06	95,59	89,44	69,89		75,92	76,38	74,27	94,75	101,94	66,14	
Sigma = 5	T1_1			575,49	632,75	604,83	683,2	731,76	609,22		513,6	586,08	590,49	612,8	608,43	578,46	
	T5_1			129,22	139,97	146,45	152,66	169,57	120,09		124,48	151,7	142,5	152,19	139,77	120,57	
	T7_1			77,83	95,26	88,57	92,66	87,63	69,89		71,88	73,55	71,47	89,76	100,07	66,14	
Sigma = 6	T1_1			538,86	591,35	578,25	662,23	702,45	609,22		482,67	562,98	565,67	588,4	584,5	578,46	
	T5_1			121,89	132,69	139,74	147,53	161,67	120,09		118,28	142,96	137,22	143,6	132,78	120,57	
	T7_1			73,3	88,31	84,8	89,64	85,65	69,89		67,69	70,32	68,62	85,11	97,72	66,14	
Sigma = 7	T1_1			503,02	551,36	551,82	639,43	673,34	609,22		451,52	536,01	540,32	563,58	560,15	578,46	
	T5_1			114,68	125,68	133,12	142,5	154,06	120,09		111,17	134,04	131,05	135,77	126,04	120,57	
	T7_1			68,81	81,64	80,94	86,66	83,57	69,89		63,51	66,83	65,78	80,93	94,97	66,14	
Sigma = 8	T1_1			468,96	513,85	526,19	615,42	644,67	609,22		421,22	506,53	515	538,84	535,83	578,46	
	T2_1			107,71	117,44	126,76	137,55	146,97	120,09		105,41	125,2	124,37	128,63	119,69	120,57	
	T7_1			64,42	75,4	77,12	83,77	81,43	69,89		59,45	63,21	62,98	77,16	91,94	66,14	
Sigma = 9	T1_1			437,18	479,34	501,59	590,73	616,57	609,22		392,44	475,91	489,98	514,45	511,91	578,46	
	T2_1			101,01	109,99	120,77	132,68	140,47	120,09		99,18	116,63	117,5	122,02	113,81	120,57	
	T7_1			60,17	69,65	73,45	81,02	79,28	69,89		55,58	59,58	60,25	73,74	88,74	66,14	
Sigma = 12	T1_1			477,09	468,4	415,75	367,15	399,82	609,22		475,76	511,68	662,49	658,72	536,91	578,46	
	T2_1			78,41	82,17	88,78	100,77	96,99	120,09		80,73	90,61	101,58	91	85,2	120,57	
	T7_1			43,15	39,54	33,74	35,73	36,8	69,89		36,41	38,81	39,24	38,85	38,17	66,14	

Table S30 shows the activity concentrations measured using WKP and with 2 mL small VOI methods on SPECTs images post-filtered with Gaussian filter (0-12 mm) for patient number 12. Whereas, RC represented patient specific recovery coefficient of right and the left kidneys, respectively.

Post-filtering (mm)	Data	Time p.i. (h)	Inj Act (MBq)	Right Kidney										Left Kidney			
			7616	VOI_1 (counts/mL)	VOI_2 (counts/mL)	VOI_3 (counts/mL)	VOI_4 (counts/mL)	VOI_5 (counts/mL)	WKP (counts/mL)	RC	VOI_1 (counts/mL)	VOI_2 (counts/mL)	VOI_3 (counts/mL)	VOI_4 (counts/mL)	VOI_5 (counts/mL)	WKP (counts/mL)	RC
sigma = 0	T1_1	22,9		491,85	587,31	584,95	591,51	646,89	409,6	0,85	448,49	479,95	493,42	650,07	671,22	418,29	0,84
	T2_1	50,1		311,95	341,81	385,42	354,54	331,61	260,19		281,16	278,66	316,63	337,6	414,53	251,38	
	T7_1	173,0		61,87	53,65	53,24	45,2	54,12	35,25		43,32	50,55	54,52	50,82	55,85	34,67	
Sigma = 3	T1_1			457,7	544,16	560,72	566,96	598,24	409,6		412,3	451,3	470,8	612,0	623,0	418,3	
	T2_1			284,87	325,25	355,52	337,74	318,72	260,2		256,8	269,6	301,8	326,5	386,2	251,4	
	T7_1			57,57	52,02	51,24	42,62	48,5	35,3		41,9	48,4	52,7	49,7	51,8	34,7	
sigma = 4	T1_1			435,71	517,01	542,92	550,14	566,04	409,6		389,74	431,61	454,59	584,66	591,68	418,29	
	T2_1			267,87	313,03	337,13	325,88	310	260,19		241,75	262,65	291,67	316,99	367,31	251,38	
	T7_1			54,82	50,73	49,63	40,88	45,17	35,25		40,85	46,98	51,28	48,89	49,31	34,67	
Sigma = 5	T1_1			412,38	488,18	522,25	530,69	531,19	409,6		366,41	409,89	435,79	553,52	557,91	418,29	
	T2_1			250,29	298,42	317,96	312,35	299,82	260,19		226,59	253,89	280,17	304,98	346,58	251,38	
	T7_1			51,86	49,13	47,66	38,97	41,87	35,25		39,49	45,41	49,62	47,8	46,71	34,67	
Sigma = 6	T1_1			389,91	459,19	499,89	509,18	495,96	409,6		343,79	387,5	415,25	520,65	523,73	418,29	
	T2_1			233,34	282,28	298,94	297,9	288,38	260,19		212,31	243,6	267,63	291,29	325,33	251,38	
	T7_1			48,85	47,26	45,42	37	38,84	35,25		37,93	43,73	47,73	46,47	44,17	34,67	
Sigma = 7	T1_1			367,75	431,3	476,76	486,27	461,97	409,6		322,82	365,39	393,81	487,82	490,6	418,29	
	T2_1			217,8	265,67	280,76	283,17	276,06	260,19		199,41	232,22	254,47	276,65	304,64	251,38	
	T7_1			45,92	45,19	43,05	35,04	36,17	35,25		36,3	42,01	45,71	44,95	41,77	34,67	
Sigma = 8	T1_1			348,38	405,26	453,5	462,57	430,1	409,6		303,89	344,07	372,12	456,18	459,3	418,29	
	T2_1			204,06	249,5	263,76	268,6	263,28	260,19		187,97	220,24	241,03	261,73	285,12	251,38	
	T7_1			43,14	42,99	40,65	33,14	33,87	35,25		34,68	40,25	43,64	43,28	39,56	34,67	
Sigma = 9	T1_1			331,36	381,44	430,55	438,63	400,73	409,6		287	323,8	350,69	426,38	430,18	418,29	
	T2_1			192,25	234,37	248,09	254,47	250,44	260,19		177,84	208,08	227,59	246,97	267,03	251,38	
	T7_1			40,56	40,74	38,28	31,32	31,92	35,25		33,14	38,49	41,58	41,52	37,52	34,67	
Sigma = 12	T1_1			291,82	322,95	366,05	369,58	327,4	409,6		246,26	269,85	291,04	349,15	355,61	418,29	
	T2_1			166,99	197,18	208,73	215,95	213,86	260,19		153,15	173,53	189,24	206,04	221,14	251,38	
	T7_1			34,05	34,19	31,79	26,43	27,61	35,25		29,37	33,51	35,9	36,2	32,3	34,67	

Table S31 shows the activity concentrations measured using WKP and with 2 mL small VOI methods on SPECTs images post-filtered with Gaussian filter (0-12 mm) for patient number 13. Whereas, RC represented patient specific recovery coefficient of right and the left kidneys, respectively.

Post-filtering (mm)	Data	Time p.i. (h)	Inj Act (MBq)	Right Kidney							Left Kidney						
			7589	VOI_1 (counts/mL)	VOI_2 (counts/mL)	VOI_3 (counts/mL)	VOI_4 (counts/mL)	VOI_5 (counts/mL)	WKP (counts/mL)	RC	VOI_1 (counts/mL)	VOI_2 (counts/mL)	VOI_3 (counts/mL)	VOI_4 (counts/mL)	VOI_5 (counts/mL)	WKP (counts/mL)	RC
sigma = 0	T1_1	22,9		417	523,16	553,35	546,5	479,09	353,04	0,85	368,33	417,53	454,84	503,39	507,22	330,61	0,84
	T2_1	50,3		358,99	430,26	434,8	397,73	328,87	290,42		310,34	360,26	340,23	424,31	364,74	259,69	
	T7_1	171,8		113,14	113,69	139,56	160,1	124,15	88,37		148,54	129,84	126,83	149,72	127,58	86,14	
Sigma = 3	T1_1			409,03	487,51	523,35	519,89	460,39	353,0		351,0	400,9	419,0	468,2	465,5	330,6	
	T2_1			331,51	407,97	412,94	374,69	311,44	290,4		288,9	341,4	321,4	390,5	341,2	259,7	
	T7_1			108,46	112,15	132,11	142,61	115,69	88,4		134,6	122,4	120,2	133,8	119,6	86,1	
sigma = 4	T1_1			401,17	463,74	501,44	500,35	445,45	353,04		337,81	387,71	396,2	445,25	438,05	330,61	
	T2_1			313,94	391,75	397,27	360,15	300,32	290,42		273,85	327,8	308,47	368,9	325,51	259,69	
	T7_1			104,67	110,01	126,32	132,41	110,32	88,37		125,76	117,72	115,86	124,57	114,55	86,14	
Sigma = 5	T1_1			390,1	437,6	475,82	477,44	427,08	353,04		321,94	371,09	371,85	420,18	408,68	330,61	
	T2_1			295,26	372,81	378,88	344,19	288,32	290,42		257,27	311,95	293,56	345,85	308,62	259,69	
	T7_1			100,08	106,7	119,39	122,31	104,53	88,37		116,53	112,78	111,19	115,38	109,03	86,14	
Sigma = 6	T1_1			376,17	410,55	447,97	452,5	406,33	353,04		304,24	351,55	347,28	394,06	379,43	330,61	
	T2_1			276,49	352,19	358,76	327,37	275,75	290,42		240,32	294,7	277,4	322,66	291,65	259,69	
	T7_1			95,07	102,39	111,77	112,82	98,56	88,37		107,56	107,73	106,46	106,65	103,33	86,14	
Sigma = 7	T1_1			359,86	383,79	419,42	426,69	384,28	353,04		285,69	330,11	323,55	367,99	351,56	330,61	
	T2_1			258,28	331,03	337,94	310,27	262,79	290,42		223,69	276,88	260,82	300,28	275,14	259,69	
	T7_1			89,98	97,41	103,95	104,18	92,65	88,37		99,3	102,75	101,93	98,63	97,66	86,14	
Sigma = 8	T1_1			342	358,04	391,34	400,91	361,86	353,04		267,12	307,84	301,23	342,76	325,65	330,61	
	T2_1			240,97	310,16	317,25	293,3	249,64	290,42		207,73	259,13	244,44	279,19	259,28	259,69	
	T7_1			85,01	92,07	96,36	96,41	86,93	88,37		91,97	98	97,78	91,44	92,14	86,14	
Sigma = 9	T1_1			323,33	333,69	364,53	375,79	339,79	353,04		249,13	285,68	280,55	318,93	301,9	330,61	
	T2_1			224,72	290,11	297,27	276,74	236,57	290,42		192,64	241,88	228,72	259,62	244,1	259,69	
	T7_1			80,27	86,62	89,23	89,42	81,53	88,37		85,66	93,65	94,09	85,09	86,86	86,14	
Sigma = 12	T1_1			268,09	270,12	294,58	307,61	279,93	353,04		201,71	225,6	227,85	257,95	243,2	330,61	
	T2_1			182,65	236,63	244,12	231,36	200,34	290,42		153,42	195,23	187,46	210,13	203,18	259,69	
	T7_1			67,49	71,05	71,36	72,2	67,52	88,37		72,86	83,74	85,35	70,8	72,81	86,14	

Table S32 shows the activity concentrations measured using WKP and with 2 mL small VOI methods on SPECTs images post-filtered with Gaussian filter (0-12 mm) for patient number 14. Whereas, RC represented patient specific recovery coefficient of right and the left kidneys, respectively.

Post-filtering (mm)	Data	Time p.i. (h)	Inj Act (MBq)	Right Kidney							Left Kidney						
			7 614	VOI_1 (counts/mL)	VOI_2 (counts/mL)	VOI_3 (counts/mL)	VOI_4 (counts/mL)	VOI_5 (counts/mL)	WKP (counts/mL)	RC	VOI_1 (counts/mL)	VOI_2 (counts/mL)	VOI_3 (counts/mL)	VOI_4 (counts/mL)	VOI_5 (counts/mL)	WKP (counts/mL)	RC
sigma = 0	T1_1	24,5		702,16	656	625,43	631,3	674,61	486,91	0,87	651,83	713,53	592,58	515,7	570,49	470	0,83
	T2_1	51,2		447,79	443,57	355,1	425,85	431,57	346,22		388,92	514,04	405,68	401,24	416,24	316,25	
	T7_1	172,5		80,47	88,78	75,63	88,46	75,17	57,37		77,22	85,86	78,02	77,42	79,54	55,8	
Sigma = 3	T1_1			655,6	617,23	599,96	606,62	640,55	486,9		614,0	665,6	583,4	506,3	552,4	470,0	
	T2_1			424,15	424,63	350,47	403,09	413,71	346,2		363,7	482,4	387,4	382,3	386,2	316,3	
	T7_1			74,49	83,75	72,36	79,09	69,45	57,4		68,7	78,8	76,9	72,7	74,5	55,8	
sigma = 4	T1_1			624,05	591,98	582,74	589,52	618,93	486,91		588,16	633,83	575,14	500,49	539,62	470	
	T2_1			407,79	409,88	346,81	388,5	401,22	346,22		347,17	460,2	376,38	369,43	367,79	316,25	
	T7_1			70,95	80,01	69,99	73,68	65,94	57,37		63,64	73,89	74,95	69,81	71,21	55,8	
Sigma = 5	T1_1			588,77	564,41	563,35	570,34	595,93	486,91		559,09	598,63	563,35	494,1	524,68	470	
	T2_1			389,18	391,87	341,54	373,34	386,87	346,22		329,46	434,94	364,97	355,13	348,87	316,25	
	T7_1			67,2	75,61	67,27	68,43	62,37	57,37		58,75	68,39	72,02	66,73	67,84	55,8	
Sigma = 6	T1_1			551,66	535,73	542,66	550,2	572,69	486,91		528,15	561,63	547,65	486,65	508,05	470	
	T2_1			369,08	371,44	334,15	358,52	371,39	346,22		311,68	407,92	353,37	340,2	330,42	316,25	
	T7_1			63,29	70,83	64,36	63,66	58,94	57,37		54,18	62,62	68,48	63,64	64,66	55,8	
Sigma = 7	T1_1			514,47	506,98	521,49	529,85	549,75	486,91		496,7	524,33	528,23	477,53	490,3	470	
	T2_1			348,19	349,61	324,47	344,35	355,47	346,22		294,57	380,44	341,52	325,24	312,93	316,25	
	T7_1			59,32	65,92	61,36	59,53	55,84	57,37		49,98	56,97	64,59	60,61	61,76	55,8	
Sigma = 8	T1_1			478,46	478,88	500,28	509,61	527,2	486,91		465,73	487,82	505,7	466,24	471,79	470	
	T2_1			327,15	327,36	312,7	330,72	339,57	346,22		278,43	353,45	329,18	310,57	296,5	316,25	
	T7_1			55,42	61,1	58,33	56,01	53,13	57,37		46,16	51,66	60,55	57,68	59,12	55,8	
Sigma = 9	T1_1			444,45	451,84	479,2	489,51	504,88	486,91		435,9	452,86	480,92	452,56	452,71	470	
	T2_1			306,46	305,48	299,3	317,33	323,93	346,22		263,27	327,62	316,16	296,27	281,03	316,25	
	T7_1			51,67	56,49	55,34	53,03	50,79	57,37		42,69	46,84	56,52	54,85	56,66	55,8	
Sigma = 12	T1_1			357,48	378,81	416,48	428,77	437,99	486,91		356,06	360,91	401,82	399,4	392,98	470	
	T2_1			249,92	246,49	254,93	276,79	279,1	346,22		222,73	260,1	273,36	255,2	238,99	316,25	
	T7_1			41,75	44,57	46,83	46,05	45,19	57,37		34,16	35,52	45,47	46,89	49,82	55,8	

Table S33 shows the activity concentrations measured using WKP and with 2 mL small VOI methods on SPECTs images post-filtered with Gaussian filter (0-12 mm) for patient number 15. Whereas, RC represented patient specific recovery coefficient of right and the left kidneys, respectively.

Post-filtering (mm)	Data	Time p.i. (h)	Inj Act (MBq)	Right Kidney							Left Kidney						
			7 620	VOI_1 (counts/mL	VOI_2 (counts/mL	VOI_3 (counts/mL	VOI_4 (counts/mL	VOI_5 (counts/mL	WKP (counts/mL)	RC	VOI_1 (counts/mL	VOI_2 (counts/mL	VOI_3 (counts/mL	VOI_4 (counts/mL	VOI_5 (counts/mL	WKP (counts/mL)	RC
sigma = 0	T1_1	20,7		643,44	676,08	515,16	540,3	638,07	419,72	0,85	490,91	511,82	526,71	504,64	552	426,56	0,84
	T2_1	48,4		359,88	288,39	346,37	380,09	338,45	243,98		385,08	350,12	297,86	357,17	350,1	235,34	
	T7_1	169,1		54,43	57,99	51,55	51,63	52,8	41,39		61,32	57,36	61,1	58,06	57,58	42,58	
Sigma = 3	T1_1			585,53	618,36	488,51	514,35	601,73	419,7		463,0	479,4	495,3	489,3	519,7	426,6	
	T2_1			328,82	273,86	329,59	355,76	321,82	244,0		346,7	331,5	281,8	333,9	332,7	235,3	
	T7_1			49,18	52,08	49,36	49	50,43	41,4		55,8	54,4	55,7	54,4	51,7	42,6	
sigma = 4	T1_1			547,71	580,75	470,78	496,2	577,14	419,72		442,84	456,47	473,26	477,73	499,35	426,56	
	T2_1			309,01	264,49	317,6	339,69	309,19	243,98		322,73	318,04	271,9	318,9	320,62	235,34	
	T7_1			45,79	48,87	47,78	47,18	48,89	41,39		52,33	52,19	52,47	52,08	48,32	42,58	
Sigma = 5	T1_1			507,13	540,01	450,9	475,63	549,5	419,72		419,88	430,74	448,34	463,16	477,55	426,56	
	T2_1			288	254,51	303,79	322	294,3	243,98		297,79	302,32	261,6	302,7	307,04	235,34	
	T7_1			42,34	45,84	45,93	45,08	47,18	41,39		48,65	49,67	49,26	49,57	45,17	42,58	
Sigma = 6	T1_1			466,53	498,41	429,64	453,87	520,21	419,72		395,75	404,02	421,9	445,89	455,34	426,56	
	T2_1			266,96	244,43	288,95	303,56	278,28	243,98		273,42	285,28	251,37	286,21	292,58	235,34	
	T7_1			39,07	43,03	43,86	42,82	45,33	41,39		45,06	47,15	46,22	47,04	42,38	42,58	
Sigma = 7	T1_1			427,82	457,93	407,67	431,89	490,58	419,72		371,78	377,6	395,12	426,63	433,49	426,56	
	T2_1			246,73	234,47	273,75	285,13	262,02	243,98		250,65	267,78	241,48	270,11	277,78	235,34	
	T7_1			36,09	40,46	41,69	40,56	43,38	41,39		41,68	44,7	43,45	44,56	40	42,58	
Sigma = 8	T1_1			392,04	419,85	385,51	410,25	461,6	419,72		348,86	352,25	368,91	406,13	412,34	426,56	
	T2_1			227,78	224,65	258,72	267,24	246,13	243,98		229,98	250,47	232,01	254,8	263,02	235,34	
	T7_1			33,4	38,09	39,52	38,38	41,38	41,39		38,55	42,32	40,92	42,18	37,97	42,58	
Sigma = 9	T1_1			359,63	384,82	363,58	389,25	433,87	419,72		327,51	328,4	343,88	385,12	392	426,56	
	T2_1			210,33	214,91	244,2	250,25	230,99	243,98		211,5	233,82	222,95	240,48	248,57	235,34	
	T7_1			30,95	35,88	37,42	36,33	39,4	41,39		35,71	40,01	38,6	39,93	36,21	42,58	
Sigma = 12	T1_1			281,89	299,2	302,14	330,64	360,34	419,72		273,49	266,98	278,98	324,37	335,61	426,56	
	T2_1			167,17	185,97	205,05	206,13	191,33	243,98		167,88	190,04	197,57	203,73	208,78	235,34	
	T7_1			24,83	30,04	31,81	31,12	33,97	41,39		28,71	33,49	32,59	33,96	32,06	42,58	

Table S34 shows the activity concentrations measured using WKP and with 2 mL small VOI methods on SPECTs images post-filtered with Gaussian filter (0-12 mm) for patient number 16. Whereas, RC represented patient specific recovery coefficient of right and the left kidneys, respectively.

Post-filtering (mm)	Data	Time p.i. (h)	Inj Act (MBq)	Right Kidney								Left Kidney				
			7 488	VOI_1 (counts/mL)	VOI_2 (counts/mL)	VOI_3 (counts/mL)	VOI_4 (counts/mL)	VOI_5 (counts/mL)	WKP (counts/mL)	RC	VOI_1 (counts/mL)	VOI_2 (counts/mL)	VOI_3 (counts/mL)	VOI_4 (counts/mL)	VOI_5 (counts/mL)	WKP (counts/mL)	RC
sigma = 0	T1_1	23,2		621,64	687,97	552,43	587,9	628,27	440,12	0,86	429,57	523,89	541,01	554,03	633,06	410,14	0,85
	T2_1	50,2		375,41	387,86	290,77	353,8	338,21	235,88		302,35	315,17	372,84	347,56	322,53	254,2	
	T7_1	171,3		71,85	68,46	58,17	71,66	61,84	45,27		74,73	72,46	72,57	82,34	76,67	44,04	
Sigma = 3	T1_1			581,47	640,83	533,26	558,38	596,4	440,1		397,9	485,8	515,5	522,2	593,2	410,1	
	T2_1			345,18	357,9	285,99	337,13	323,92	235,9		274,8	305,0	342,2	333,4	319,9	254,2	
	T7_1			64,3	63,23	53,25	65,74	58,53	45,3		65,1	67,7	68,4	73,2	69,5	44,0	
sigma = 4	T1_1			554,27	610,34	520,54	538,03	575,12	440,12		376,44	461,46	497,42	499,98	567,28	410,14	
	T2_1			352,39	338,54	282,28	325,53	313,77	235,88		258,53	296,4	323,11	323,4	315,31	254,2	
	T7_1			59,61	59,89	50,37	62,51	56,71	45,27		59,84	64,56	65,38	67,9	65,21	44,04	
Sigma = 5	T1_1			524,24	577,09	506,68	515,91	551,95	440,12		353,61	435,56	476,44	474,44	539,64	410,14	
	T2_1			304,16	317,9	277,74	312,71	302,05	235,88		242,27	285,49	303,17	311,51	307,81	254,2	
	T7_1			54,91	56,41	47,56	59,39	54,95	45,27		54,88	61,33	61,93	62,82	60,83	44,04	
Sigma = 6	T1_1			493,17	542,77	492,43	493,67	528,34	440,12		331,31	409,58	453,5	447,08	511,63	410,14	
	T2_1			282,95	297,34	272,42	299,57	289,36	235,88		226,9	272,9	283,45	298,01	297,79	254,2	
	T7_1			50,57	53,01	44,95	56,44	53,29	45,27		50,47	58,13	58,37	58,19	56,64	44,04	
Sigma = 7	T1_1			462,6	508,93	478,03	472,19	505,18	440,12		310,7	384,66	429,56	419,43	484,19	410,14	
	T2_1			262,91	277,87	266,35	286,82	276,34	235,88		212,9	259,36	264,66	283,35	285,86	254,2	
	T7_1			46,73	49,83	42,67	53,73	51,76	45,27		46,66	54,99	54,9	54,09	52,79	44,04	
Sigma = 8	T1_1			433,5	476,56	463,42	451,81	482,84	440,12		292,32	361,4	405,4	392,6	457,74	410,14	
	T2_1			244,67	260	259,55	274,79	263,51	235,88		200,41	245,5	247,09	268,03	272,7	254,2	
	T7_1			43,42	46,93	40,72	51,28	50,34	45,27		43,36	51,92	51,64	50,48	49,29	44,04	
Sigma = 9	T1_1			406,4	446,21	448,39	432,53	461,43	440,12		276,26	340,03	381,65	367,23	432,37	410,14	
	T2_1			228,45	243,88	252,11	263,58	251,26	235,88		189,36	231,8	230,78	252,52	258,91	254,2	
	T7_1			40,58	44,3	39,07	49,07	48,98	45,27		40,46	48,92	48,62	47,29	46,13	44,04	
Sigma = 12	T1_1			338,27	368,58	400,01	379,92	402,46	440,12		239,71	286,38	316,44	301,69	362,7	410,14	
	T2_1			190,98	205,03	227,09	233,68	219,37	235,88		162,9	194	188,79	208,27	217,61	254,2	
	T7_1			34,33	37,92	35,33	43,48	44,79	45,27		33,35	40,59	40,86	39,56	38,34	44,04	

Table S35 shows the activity concentrations measured using WKP and with 2 mL small VOI methods on SPECTs images post-filtered with Gaussian filter (0-12 mm) for patient number 17. Whereas, RC represented patient specific recovery coefficient of right and the left kidneys, respectively.

Post-filtering (mm)	Data	Time p.i. (h)	Inj Act (MBq)	Right Kidney							Left Kidney						
			7584	VOI_1 (counts/mL)	VOI_2 (counts/mL)	VOI_3 (counts/mL)	VOI_4 (counts/mL)	VOI_5 (counts/mL)	WKP (counts/mL)	RC	VOI_1 (counts/mL)	VOI_2 (counts/mL)	VOI_3 (counts/mL)	VOI_4 (counts/mL)	VOI_5 (counts/mL)	WKP (counts/mL)	RC
sigma = 0	T1_1	23,0		627,54	774,64	830,03	817,43	796,97	624,72	0,84	790,09	810,18	691,99	678,01	653,14	692,25	0,85
	T2_1	50,8		475,04	470,74	466,98	546,8	611,03	423,35		530,45	556,71	577,12	535,23	481,77	463,18	
	T7_1	171,7		99,91	103,63	77,56	86,46	80,36	71,03		89,71	97,83	87,46	104,36	103	70,29	
Sigma = 3	T1_1			588,52	725,5	773,53	761,49	759,07	624,7		743,1	760,7	669,1	654,4	626,9	692,3	
	T2_1			437,51	445,3	447,39	528,18	579,09	423,4		493,1	518,3	534,8	514,7	456,6	463,2	
	T7_1			92,69	96,94	77,34	82,57	79,03	71,0		82,7	89,5	80,3	97,3	96,7	70,3	
sigma = 4	T1_1			564,24	691,88	735,98	725,6	732,96	624,72		711,32	726,87	654,2	638,73	610,28	692,25	
	T2_1			414,09	427,15	434,87	513,23	557,35	423,35		469,69	492,84	506,45	499,52	441,03	463,18	
	T7_1			88,12	92,1	76,58	80	77,77	71,03		78,28	84,14	76,08	92,49	92,44	70,29	
Sigma = 5	T1_1			538,97	654,44	695,01	687,45	703,23	624,72		675,94	688,72	637,69	621,83	592,75	692,25	
	T2_1			389,47	406,23	421,28	495,21	532,96	423,35		444,93	464,71	475,29	481,64	425,22	463,18	
	T7_1			83,16	86,56	75,15	77,1	76,08	71,03		73,59	78,53	71,79	87,21	87,89	70,29	
Sigma = 6	T1_1			513,98	615,38	652,98	649,2	671,24	624,72		638,88	648,09	619,76	604,91	575,39	692,25	
	T2_1			364,89	383,55	407,15	475,29	507,01	423,35		420,06	435,28	443,29	461,77	410,2	463,18	
	T7_1			77,98	80,75	73,09	74,35	74,02	71,03		68,87	72,96	67,59	81,82	83,31	70,29	
Sigma = 7	T1_1			489,84	576,44	611,62	612,25	638,31	624,72		601,87	606,91	600,41	588,68	558,9	692,25	
	T2_1			341,31	360,22	392,65	454,34	480,38	423,35		395,94	405,82	411,95	440,58	396,32	463,18	
	T7_1			72,83	75,03	70,54	71,48	71,63	71,03		64,35	67,65	63,59	76,67	78,94	70,29	
Sigma = 8	T1_1			466,59	538,75	571,96	577,14	605,48	624,72		566,1	566,66	579,71	573,25	543,53	692,25	
	T2_1			319,19	337,13	377,77	432,91	453,71	423,35		373,01	377,28	382,2	418,73	383,41	463,18	
	T7_1			67,88	69,61	67,67	68,63	68,98	71,03		60,15	62,7	59,84	71,94	74,9	70,29	
Sigma = 9	T1_1			444,14	503	534,54	544,01	573,41	624,72		532,28	528,38	557,85	558,33	529,22	692,25	
	T2_1			298,75	314,9	362,47	411,36	427,44	423,35		351,44	350,31	354,58	396,78	371,09	463,18	
	T7_1			63,2	64,59	64,62	65,8	66,14	71,03		56,31	58,17	56,33	67,72	71,23	70,29	
Sigma = 12	T1_1			380,81	409,67	437,22	455,63	484,49	624,72		444,76	429,73	489,26	512,44	489,52	692,25	
	T2_1			247,03	256,35	314,49	348,08	353,95	423,35		295,07	281,7	285,89	334,77	334,79	463,18	
	T7_1			51,1	52,07	55,37	57,44	57,2	71,03		46,97	47,07	47,26	57,84	62,36	70,29	

Table S36 shows the activity concentrations measured using WKP and with 2 mL small VOI methods on SPECTs images post-filtered with Gaussian filter (0-12 mm) for patient number 18. Whereas, RC represented patient specific recovery coefficient of right and the left kidneys, respectively.

Post-filtering (mm)	Data	Time p.i. (h)	Inj Act (MBq)	Right Kidney							Left Kidney						
			7252	VOI_1 (counts/mL)	VOI_2 (counts/mL)	VOI_3 (counts/mL)	VOI_4 (counts/mL)	VOI_5 (counts/mL)	WKP (counts/mL)	RC	VOI_1 (counts/mL)	VOI_2 (counts/mL)	VOI_3 (counts/mL)	VOI_4 (counts/mL)	VOI_5 (counts/mL)	WKP (counts/mL)	RC
sigma = 0	T1_1	22,7		863,96	1092,31	1082,74	1043,51	1007,75	780,59	0,85	944,76	953,18	879,38	1035,97	902,16	776,11	0,84
	T2_1	50,8		640,21	735,24	826,17	767,54	658,42	522,33		606,89	581,53	600,08	723,22	677,14	505,82	
	T7_1	172,2		129,6	177,3	134,05	134,97	122,46	93,34		131,75	162,18	182,48	196,71	160,44	109,71	
Sigma = 3	T1_1			849,27	1050,32	1016	993,46	955,85	780,6		877,0	897,6	828,9	960,3	872,2	776,1	
	T2_1			614,07	693,46	762,6	715,96	629,17	522,3		550,7	556,2	553,9	664,8	641,0	505,8	
	T7_1			118,09	158,56	132,59	127,74	117,06	93,3		127,3	157,9	173,2	178,3	155,7	109,7	
sigma = 4	T1_1			834,17	1018,15	971,58	956,9	920,75	780,59		829,45	858,8	795,72	912,13	849,71	776,11	
	T2_1			595,24	664,16	720,79	681,5	609,07	522,33		514,87	535	523,79	627,52	616,95	505,82	
	T7_1			111,4	147,53	129,86	122,77	113,12	93,34		124,27	155,05	167,57	167,33	151,43	109,71	
Sigma = 5	T1_1			812,58	979,08	922,66	914,48	882,02	780,59		775,78	813,78	758,63	861,26	822,95	776,11	
	T2_1			572,79	630,71	675,62	643,55	586,3	522,33		477	508,15	491,51	588,05	590,34	505,82	
	T7_1			104,67	136,6	125,56	117,16	108,41	93,34		120,91	151,92	161,98	156,45	145,89	109,71	
Sigma = 6	T1_1			785,21	934,93	871,86	868,77	842,11	780,59		719,43	764,58	719,08	810,7	793,17	776,11	
	T2_1			547,51	594,9	629,86	604,35	562,02	522,33		439,36	477,19	459	548,81	562,27	505,82	
	T7_1			98,17	126,29	120,01	111,25	103,17	93,34		117,51	148,7	156,72	146,25	139,23	109,71	
Sigma = 7	T1_1			753,41	887,77	821,27	822,06	802,85	780,59		663,54	713,51	678,72	762,67	761,72	776,11	
	T2_1			520,42	558,41	585,67	565,82	537,36	522,33		403,65	444,1	427,53	511,6	533,8	505,82	
	T7_1			92,05	116,82	113,68	105,36	97,64	93,34		114,22	145,49	151,91	136,98	131,89	109,71	
Sigma = 8	T1_1			718,81	839,44	772,21	776,05	765,36	780,59		610,32	662,58	638,8	718,2	729,7	776,11	
	T2_1			492,48	522,49	544,24	529,26	513,16	522,33		370,8	410,71	397,77	477,33	505,71	505,82	
	T7_1			86,34	108,15	107,01	99,77	92,06	93,34		111,11	142,26	147,5	128,62	124,27	109,71	
Sigma = 9	T1_1			682,94	791,33	725,44	731,95	730,19	780,59		561,1	613,35	600,16	677,48	697,89	776,11	
	T2_1			464,55	487,98	506,11	495,37	489,96	522,33		341,19	378,42	369,95	446,25	478,55	505,82	
	T7_1			81,01	100,21	100,38	94,65	86,63	93,34		108,19	138,98	143,33	121,05	116,72	109,71	
Sigma = 12	T1_1			578	656,69	601,45	616,03	638,41	780,59		440,81	484,19	495,58	573,81	607,67	776,11	
	T2_1			386,15	396,84	411,26	410,87	428,18	522,33		270,96	295,2	298,34	369,8	405,54	505,82	
	T7_1			67,05	80,17	82,51	82,5	71,99	93,34		100,38	128,23	130,95	101,98	96,19	109,71	

Table S37 shows the activity concentrations measured using WKP and with 4 mL small VOI methods on SPECTs images post-filtered with Gaussian filter (0-12 mm) for patient number 1. Whereas, RC represented patient specific recovery coefficient of right and the left kidneys, respectively.

Post-filtering (mm)	Data	Time p.i. (h)	Inj Act (MBq)	Right Kidney							Left Kidney						
			7672	VOI_1 (counts/mL)	VOI_2 (counts/mL)	VOI_3 (counts/mL)	VOI_4 (counts/mL)	VOI_5 (counts/mL)	WKP (counts/mL)	RC	VOI_1 (counts/mL)	VOI_2 (counts/mL)	VOI_3 (counts/mL)	VOI_4 (counts/mL)	VOI_5 (counts/mL)	WKP (counts/mL)	RC
sigma = 0	T1_1	21,0		833,64	807,32	880,9	786,65	689,29	643,51	0,89	924,07	1028,61	900,77	678,41	822,8	593,48	0,86
	T2_1	48,8		583,94	534,74	669,29	548,6	648,05	447,5		750,45	660,44	533,39	518,77	576,58	405,35	
	T7_1	170,4		87,76	97,23	118,33	105,04	112,39	76,42		152,54	112,11	108,22	102,83	119,54	65,31	
Sigma = 3	T1_1			786,49	779,93	826,84	743,04	647,87	643,51		850,59	968,13	835,91	651,16	750,02	593,48	
	T2_1			538,78	510,23	622,14	520,29	603,58	447,5		695,95	616,06	493,16	481,05	529,58	405,35	
	T7_1			90,96	110,39	97,47	102,41	97,34	76,42		141,3	103,1	102,18	96,6	109,97	65,31	
sigma = 4	T1_1			754,62	759,41	791,09	713,16	621,36	643,51		802,15	925,03	792,9	630,92	702,58	593,48	
	T2_1			508,56	492,66	592,59	501,7	574,34	447,5		658,83	586,76	465,37	455,61	498,68	405,35	
	T7_1			86,7	105,17	92,56	96,38	91,57	76,42		133,5	97,32	97,98	92,43	103,76	65,31	
sigma = 5	T1_1			718,78	734,64	752,66	679,88	592,77	643,51		749,59	875,17	745,49	607,63	651,46	593,48	
	T2_1			475,37	473,08	561,39	481,46	542,31	447,5		617,37	554,44	434,62	427,77	465,04	405,35	
	T7_1			82,03	99,62	87,39	90,2	85,34	76,42		124,64	91,02	93,17	87,77	97,17	65,31	
sigma = 6	T1_1			680,78	706,69	713,87	645,48	563,6	643,51		696,05	821	696,4	582,51	599,95	593,48	
	T2_1			441,31	452,75	530,38	460,97	509,22	447,5		574	520,76	403,06	399,25	430,67	405,35	
	T7_1			77,25	94,13	82,29	84,16	79,02	76,42		115,28	84,56	88,01	82,87	90,62	65,31	
Sigma = 7	T1_1			642,23	676,77	676,43	611,95	535,07	643,51		644,13	765,03	647,93	556,34	550,67	593,48	
	T2_1			408,09	432,51	500,89	441,35	476,61	447,5		530,8	487,07	372,42	371,34	397,17	405,35	
	T7_1			72,59	88,93	77,43	78,47	72,91	76,42		105,93	78,22	82,73	77,93	84,36	65,31	
Sigma = 8	T1_1			604,23	645,91	641,22	580,6	507,9	643,51		595,42	709,41	601,59	529,59	505,19	593,48	
	T2_1			376,76	412,77	473,6	423,23	445,5	447,5		489,25	454,24	343,85	344,86	365,6	405,35	
	T7_1			68,21	84,17	72,9	73,2	67,16	76,42		96,96	72,19	77,52	73,1	78,51	65,31	
Sigma = 9	T1_1			567,48	614,92	608,6	552,14	482,48	643,51		550,61	655,73	558,18	502,54	464,22	593,48	
	T2_1			347,85	393,71	448,72	406,76	416,47	447,5		450,24	422,79	317,9	320,22	336,47	405,35	
	T7_1			64,21	79,89	68,72	68,34	61,88	76,42		88,64	66,57	72,5	68,46	73,09	65,31	
Sigma = 12	T1_1			467,85	525,6	525,32	483,83	417,36	643,51		439,62	515,67	446,81	422,1	368,08	593,48	
	T2_1			276,21	340,53	386,94	364,67	343,32	447,5		352,17	338,88	256,55	258,7	264,61	405,35	
	T7_1			54,64	69,6	57,93	55,79	48,84	76,42		68,54	52,44	59,1	56,12	59,17	65,31	

Table S38 shows the activity concentrations measured using WKP and with 4 mL small VOI methods on SPECTs images post-filtered with Gaussian filter (0-12 mm) for patient number 2. Whereas, RC represented patient specific recovery coefficient of right and the left kidneys, respectively.

Post-filtering (mm)	Data	Time p.i. (h)	Inj Act (MBq)	Right Kidney							Left Kidney						
			7692	VOI_1 (counts/mL)	VOI_2 (counts/mL)	VOI_3 (counts/mL)	VOI_4 (counts/mL)	VOI_5 (counts/mL)	WKP (counts/mL)	RC	VOI_1 (counts/mL)	VOI_2 (counts/mL)	VOI_3 (counts/mL)	VOI_4 (counts/mL)	VOI_5 (counts/mL)	WKP (counts/mL)	RC
sigma = 0	T1_1	22,8		897,18	912,67	945,1	949,67	864,68	637,65	0,89	898,05	983,71	1009,93	939,45	809,29	661,07	0,89
	T2_1	50,4		634,95	624,21	644,42	653,62	654,65	545,18		713,34	726,25	688,78	679,16	666,01	523,75	
	T7_1	171,3		125,84	118,01	137,29	124,93	109,66	103,62		127,96	120,27	120,19	127,04	102,72	105,79	
Sigma = 3	T1_1			843,9	861,76	898,27	902,47	807,11	637,65		843,89	938,89	964,64	893,6	752,34	661,07	
	T2_1			601,37	597,63	619,09	631,39	612,7	545,18		678,61	704,99	662,02	640,94	627,24	523,75	
	T7_1			114,01	111,24	126,46	121,39	103,41	103,62		120,89	115,98	116,99	120,43	95,99	105,79	
sigma = 4	T1_1			810,11	827,21	867,1	867,96	768,35	637,65		805,61	907,68	933,34	861,56	715	661,07	
	T2_1			577,51	578,77	600,62	614,54	585,59	545,18		653,08	686,39	642,36	614,82	601,02	523,75	
	T7_1			106,86	106,94	119,98	118,33	99,22	103,62		115,74	112,88	114,57	115,63	91,38	105,79	
sigma = 5	T1_1			773,77	788,41	832,09	828,09	724,99	637,65		762,11	871,76	897,53	825,02	677,35	661,07	
	T2_1			549,78	556,84	578,92	594,05	556,59	545,18		622,61	662,28	619,05	585,3	571,34	523,75	
	T7_1			99,53	102,33	113,56	114,39	94,5	103,62		109,85	109,07	111,51	110,06	86,36	105,79	
sigma = 6	T1_1			736,82	747,38	794,6	784,82	679,42	637,65		716,02	832,7	858,52	786	693,03	661,07	
	T2_1			519,59	532,77	555,12	570,61	527,24	545,18		588,6	633,84	593,19	553,93	539,46	523,75	
	T7_1			92,43	97,59	107,55	109,74	89,51	103,62		103,56	104,71	107,89	104,01	81,24	105,79	
Sigma = 7	T1_1			700,7	705,98	755,99	740,03	633,7	637,65		669,6	792,2	817,67	746,2	602,47	661,07	
	T2_1			488,36	507,43	530,26	545,09	498,49	545,18		552,61	602,67	565,98	522,11	506,64	523,75	
	T7_1			85,81	92,86	102,05	104,61	84,47	103,62		97,19	100,02	103,84	97,76	76,29	105,79	
Sigma = 8	T1_1			666,15	665,52	717,24	695,16	589,27	637,65		624,46	751,58	776,13	706,75	568,31	661,07	
	T2_1			457,22	481,58	505,17	518,35	470,7	545,18		516,09	570,37	538,35	490,81	473,91	523,75	
	T7_1			79,78	88,2	97	99,23	79,54	103,62		90,98	95,17	99,48	91,54	71,64	105,79	
Sigma = 9	T1_1			633,48	626,86	679,04	651,22	564,97	637,65		581,6	711,79	734,79	668,27	536,66	661,07	
	T2_1			427	455,8	480,4	491,19	443,96	545,18		480,21	538,18	510,88	460,63	442,07	523,75	
	T7_1			74,34	83,63	92,28	93,81	74,79	103,62		85,07	90,34	94,94	85,48	67,33	105,79	
Sigma = 12	T1_1			546,06	524,86	571,37	530,56	436,53	637,65		470,76	602,32	617,63	560,88	455,14	661,07	
	T2_1			346,08	382,79	410,86	412,48	369,92	545,18		383,72	449,31	432,33	378,91	356,39	523,75	
	T7_1			61,13	70,85	79,44	78,58	61,94	103,62		69,84	76,78	81,21	69,08	56,31	105,79	

Table S39 shows the activity concentrations measured using WKP and with 4 mL small VOI methods on SPECTs images post-filtered with Gaussian filter (0-12 mm) for patient number 3. Whereas, RC represented patient specific recovery coefficient of right and the left kidneys, respectively.

Post-filtering (mm)	Data	Time p.i. (h)	Inj Act (MBq)	Right Kidney							Left Kidney						
			7670	VOI_1 (counts/mL)	VOI_2 (counts/mL)	VOI_3 (counts/mL)	VOI_4 (counts/mL)	VOI_5 (counts/mL)	WKP (counts/mL)	RC	VOI_1 (counts/mL)	VOI_2 (counts/mL)	VOI_3 (counts/mL)	VOI_4 (counts/mL)	VOI_5 (counts/mL)	WKP (counts/mL)	RC
sigma = 0	T1_1	23,0		917,5	1428,28	1256,91	1068,42	995,99	903,77	0,81	1634,21	1568,75	1769,63	1807,13	1441,37	1233,85	0,90
	T2_1	50,2		872,26	1024,54	895,52	776,63	751,21	683,92		1001,89	1057,84	1206,94	1089,62	892,76	864,09	
	T7_1	169,5		279,34	362,38	259,21	212,48	231,95	207,99		207,86	269,35	264,79	279,66	262,98	193,94	
Sigma = 3	T1_1			884,35	1313,04	1151,39	977,32	910,75	903,77		1538,79	1494,61	1690,64	1739,97	1357,73	1233,85	
	T2_1			809,3	945,71	818,94	716,35	676,55	683,92		945,44	1007,58	1155,56	1048,64	856,94	864,09	
	T7_1			256,46	320,53	239,69	189,49	210,82	207,99		200,32	256,16	259,34	271,62	253,79	193,94	
sigma = 4	T1_1			861,63	1236,49	1083,82	917,94	853,79	903,77		1475,34	1444,167	1635,29	1690,61	1299,46	1233,85	
	T2_1			769,38	893	770,08	676,5	629,23	683,92		906,48	973,5	1119,25	1020,35	828,85	864,09	
	T7_1			241,38	294,26	226,74	175,15	197,08	207,99		194,13	247,08	254,5	265,72	246,86	193,94	
sigma = 5	T1_1			836,04	1153,29	1012,16	854	791,6	903,77		1405,24	1380,46	1571,59	1631,81	1233	1233,85	
	T2_1			726,68	835,19	718,86	632,99	579,34	683,92		863,23	935,99	1077,04	987,63	794,41	864,09	
	T7_1			225,22	267,31	212,75	160,39	182,47	207,99		186,31	236,79	248,16	258,67	238,41	193,94	
sigma = 6	T1_1			808,8	1068,63	940,6	789,27	728,27	903,77		1332,03	1314,16	1503,11	1566,1	1161,71	1233,85	
	T2_1			683,69	775,82	668,19	588,34	529,92	683,92		818,39	897,33	1031,05	952,06	755,33	864,09	
	T7_1			209,1	241,52	198,5	146,12	168,03	207,99		177,28	225,81	240,52	250,72	228,83	193,94	
Sigma = 7	T1_1			780,78	986,71	871,94	726,63	667,04	903,77		1258,58	1245,85	1433,4	1496,38	1088,87	1233,85	
	T2_1			642,4	717,91	619,89	544,62	483,2	683,92		774,14	859,31	983,49	915,16	713,56	864,09	
	T7_1			193,9	217,99	184,56	132,89	154,51	207,99		167,62	214,57	231,86	242,18	218,6	193,94	
Sigma = 8	T1_1			752,4	910,12	807,57	667,72	609,89	903,77		1186,79	1177,95	1365,11	1425,21	1017,09	1233,85	
	T2_1			603,85	663,43	574,71	503,12	440,35	683,92		731,82	823,09	936,15	878,04	670,91	864,09	
	T7_1			180,08	197,13	171,2	120,93	142,31	207,99		157,84	203,39	222,54	233,36	208,13	193,94	
Sigma = 9	T1_1			723,85	840,16	748,01	613,36	557,77	903,77		1117,91	1112,14	1299,97	1354,68	948,26	1233,85	
	T2_1			568,42	613,44	532,83	464,5	401,79	683,92		692,14	789,35	890,38	841,45	628,85	864,09	
	T7_1			167,82	178,92	158,58	110,27	131,58	207,99		148,29	192,49	212,92	224,53	197,76	193,94	
Sigma = 12	T1_1			637,78	670,65	597,42	478,66	432,1	903,77		934,83	935,56	1129,57	1159,61	768,47	1233,85	
	T2_1			479,18	491,92	425,86	367,18	310,36	683,92		590,48	704,2	769,01	738,07	514,61	864,09	
	T7_1			139,46	137,89	125,7	85,24	107,23	207,99		122,69	162,54	184,96	199,51	169,25	193,94	

Table S40 shows the activity concentrations measured using WKP and with 4 mL small VOI methods on SPECTs images post-filtered with Gaussian filter (0-12 mm) for patient number 4. Whereas, RC represented patient specific recovery coefficient of right and the left kidneys, respectively.

Post-filtering (mm)	Data	Time p.i. (h)	Inj Act (MBq)	Right Kidney							Left Kidney						
			7777	VOI_1 (counts/mL)	VOI_2 (counts/mL)	VOI_3 (counts/mL)	VOI_4 (counts/mL)	VOI_5 (counts/mL)	WKP (counts/mL)	RC	VOI_1 (counts/mL)	VOI_2 (counts/mL)	VOI_3 (counts/mL)	VOI_4 (counts/mL)	VOI_5 (counts/mL)	WKP (counts/mL)	RC
sigma = 0	T1_1	23,3		932,46	1186,76	1132,97	1159,95	1151,88	879,22	0,87	885,63	1050,51	934,99	934,45	995,93	829,16	0,86
	T2_1	51,1		553,49	701,92	719,5	723,42	615,9	479,36		683,79	694,77	753,49	683,32	632,43	528,34	
	T9_1	217,8		79,6	86,43	73,95	67,82	63,97	46,72		68,14	64,25	66,81	74,4	79,29	47,8	
Sigma = 3	T1_1			873,88	1111,46	1055,74	1113,15	1070,83	879,22		826,23	994,1	890,72	877,1	928,67	829,16	
	T2_1			529,94	659,89	673,45	681,96	598,26	479,36		657,18	663,87	704,36	647,84	603,98	528,34	
	T9_1			74,31	79,62	70,15	63,58	59,99	46,72		62,13	59,75	61,72	67,35	71,07	47,8	
sigma = 4	T1_1			837,22	1061,29	1006,86	1077,73	1017,94	879,22		787,64	955,74	859,64	838,99	884,01	829,16	
	T2_1			513,83	631,48	642,63	652,72	584,15	479,36		637,62	642,41	672,32	632,86	582,76	528,34	
	T9_1			70,95	75,26	67,33	60,68	57,23	46,72		58,19	56,87	58,47	63,04	66,03	47,8	
sigma = 5	T1_1			798,85	1006,46	954,98	1035,8	959,91	879,22		746,93	912,34	824,5	796,1	834,83	829,16	
	T2_1			495,76	600,18	608,79	619,02	565,88	479,36		614,32	618,25	637,82	596,85	557,02	528,34	
	T9_1			67,35	70,56	64	57,38	54,05	46,72		54,01	53,81	55,03	58,72	60,88	47,8	
sigma = 6	T1_1			761,36	950,05	902,72	989,19	899,56	879,22		707,17	866,47	787,23	750,39	783,81	829,16	
	T2_1			476,73	567,99	573,8	582,47	543,79	479,36		588,36	592,96	602,75	568,13	527,94	528,34	
	T9_1			63,7	65,81	60,35	53,82	50,62	46,72		49,86	50,76	51,63	54,67	55,91	47,8	
Sigma = 7	T1_1			726,5	894,73	852,09	939,67	839,38	879,22		670,7	820,47	749,57	703,91	733,2	829,16	
	T2_1			457,67	536,46	539,1	544,72	518,59	479,36		560,98	567,86	568,51	538,84	496,89	528,34	
	T7_1			60,16	61,21	56,58	50,18	47,12	46,72		45,92	47,84	48,48	51,05	51,31	47,8	
Sigma = 8	T1_1			695,09	842,26	804,14	888,77	781,07	879,22		638,86	776	712,68	658,18	684,48	829,16	
	T2_1			439,23	506,55	505,52	507,06	491,22	479,36		533,25	543,86	535,93	509,7	465,16	528,34	
	T7_1			56,8	56,88	52,83	46,58	43,68	46,72		42,25	45,13	45,71	47,9	47,14	47,8	
Sigma = 9	T1_1			667,21	793,65	759,27	837,74	725,69	879,22		612,13	734,11	677,2	614,19	638,5	829,16	
	T2_1			421,81	478,7	473,48	470,49	462,69	479,36		505,99	521,44	505,47	481,17	433,83	528,34	
	T7_1			53,67	52,88	49,19	43,11	40,37	46,72		38,9	42,65	43,41	45,18	43,39	47,8	
Sigma = 12	T1_1			599,49	672,3	642,25	692,47	581,59	879,22		558,67	627,62	580,42	497,38	519,76	829,16	
	T2_1			376,38	407,8	387,99	372,76	378,52	479,36		431,73	463,82	428,04	401,41	348,82	528,34	
	T7_1			45,67	42,87	39,42	34,03	31,78	46,72		30,67	36,55	39,4	39,06	34,3	47,8	

Table S41 shows the activity concentrations measured using WKP and with 4 mL small VOI methods on SPECTs images post-filtered with Gaussian filter (0-12 mm) for patient number 5. Whereas, RC represented patient specific recovery coefficient of right and the left kidneys, respectively.

Post-filtering (mm)	Data	Time p.i. (h)	Inj Act (MBq)	Right Kidney							Left Kidney						
			7726	VOI_1 (counts/mL)	VOI_2 (counts/mL)	VOI_3 (counts/mL)	VOI_4 (counts/mL)	VOI_5 (counts/mL)	WKP (counts/mL)	RC	VOI_1 (counts/mL)	VOI_2 (counts/mL)	VOI_3 (counts/mL)	VOI_4 (counts/mL)	VOI_5 (counts/mL)	WKP (counts/mL)	RC
sigma = 0	T1_1	19,9		1207,71	1207,71	1589,34	1511,8	1253,85	1011,9	0,87	859,9	974,72	911,61	1009,71	1095,12	708,57	0,74
	T2_1	43,8		952,35	942,28	1014,95	1050,38	854,19	846,1		691,95	838,09	899,97	719,54	725,06	599,16	
	T7_1	169,2		183,41	177,74	174,44	174,19	146,04	140,46		134,4	115,98	146,99	150,7	148,06	98,76	
Sigma = 3	T1_1			1143,08	1332,64	1494,14	1404,08	1184,03	1011,9		794,06	908,95	860,4	956,95	977,09	708,57	
	T2_1			883,8	899,67	951	986,6	847,85	846,1		633,72	756,36	816,61	679,17	681,33	599,16	
	T7_1			173,69	172,86	168,96	166,87	141,45	140,46		123,63	108,36	133,7	138,92	131,68	98,76	
sigma = 4	T1_1			1096,79	1292,64	1430,64	1333,91	1135,87	1011,9		750,21	861,19	824,43	917,34	902,35	708,57	
	T2_1			836,98	870,46	911,18	946,92	842,17	846,1		595,65	702,44	761,55	649,63	648,42	599,16	
	T7_1			168,7	169,47	165,02	161,39	137,32	140,46		116,3	103,36	125,6	130,54	121,91	98,76	
sigma = 5	T1_1			1043	1246,33	1360,51	1258,08	1081,05	1011,9		701,73	805,24	783,63	870,24	824,44	708,57	
	T2_1			785,11	837,25	869,58	905,28	833,74	846,1		554,05	644,47	702,36	614,84	609,72	599,16	
	T7_1			162,99	165,47	160,42	154,72	131,94	140,46		108,28	98,08	117,17	121,25	111,91	98,76	
sigma = 6	T1_1			984,61	1195,33	1287,46	1180,58	1022,39	1011,9		651,18	744,57	740,35	818,24	748,55	708,57	
	T2_1			731,45	801,48	828,29	864,07	821,67	846,1		511,17	586,44	642,79	576,84	567,59	599,16	
	T7_1			156,72	160,9	155,26	147,2	125,59	140,46		100,12	92,81	108,92	111,81	102,23	98,76	
Sigma = 7	T1_1			924,42	1141,34	1214,58	1104,62	962,63	1011,9		600,81	682,76	696,52	764,03	678,08	708,57	
	T2_1			678,69	764,43	788,49	824,67	805,13	846,1		468,99	531,25	585,61	537,79	524,38	599,16	
	T7_1			150,02	155,82	149,63	139,25	118,62	140,46		92,26	87,75	101,2	102,81	93,22	98,76	
Sigma = 8	T1_1			864,66	1085,72	1143,89	1032,15	903,79	1011,9		552,26	622,72	653,47	709,95	614,59	708,57	
	T2_1			628,55	727,04	750,55	787,47	783,76	846,1		428,93	480,57	532,5	499,44	482	599,16	
	T7_1			143,02	150,31	143,64	131,21	111,39	140,46		84,96	82,99	94,18	94,62	85,03	98,76	
Sigma = 9	T1_1			806,89	1029,57	1076,47	964,22	847,16	1011,9		506,64	566,48	612,11	657,82	558,53	708,57	
	T2_1			581,99	689,9	714,45	752,25	757,79	846,1		391,81	435,16	484,28	462,98	441,84	599,16	
	T7_1			135,85	144,46	137,41	123,32	104,21	140,46		78,36	78,55	87,91	87,42	77,66	98,76	
Sigma = 12	T1_1			653,5	865,93	897,03	790,1	696,07	1011,9		392,29	428,91	502,54	522,58	431	708,57	
	T2_1			466,24	583,73	615,35	654,96	660,99	846,1		300,93	330,49	370,12	369,81	341,15	599,16	
	T7_1			114,68	126,02	118,55	101,8	84,94	140,46		62,82	67,2	73,32	71,58	60,16	98,76	

Table S42 shows the activity concentrations measured using WKP and with 4 mL small VOI methods on SPECTs images post-filtered with Gaussian filter (0-12 mm) for patient number 6. Whereas, RC represented patient specific recovery coefficient of right and the left kidneys, respectively.

Post-filtering (mm)	Data	Time p.i. (h)	Inj Act (MBq)	Right Kidney							Left Kidney						
			7723	VOI_1 (counts/mL)	VOI_2 (counts/mL)	VOI_3 (counts/mL)	VOI_4 (counts/mL)	VOI_5 (counts/mL)	WKP (counts/mL)	RC	VOI_1 (counts/mL)	VOI_2 (counts/mL)	VOI_3 (counts/mL)	VOI_4 (counts/mL)	VOI_5 (counts/mL)	WKP (counts/mL)	RC
sigma = 0	T1_1	23,2		929,84	1026,27	1005,45	983,06	914,38	742,34	0,85	952,36	888,66	1013,85	855,22	760,1	732,77	0,88
	T2_1	47,3		751,5	709,19	816,61	694,1	704,32	554,34		565,69	732,84	668,4	595,7	639,84	547,84	
	T7_1	170,7		184,2	190,7	169,77	176,58	168,47	134,56		192,78	201,4	184,83	175,66	158,07	132,64	
Sigma = 3	T1_1			879,55	978,58	968,86	932,51	850,22	742,34		891,6	854,31	966,15	804,91	722,21	732,77	
	T2_1			700,1	685,52	779,52	660,11	664,99	554,34		535,9	686,78	636,3	567,53	605,29	547,84	
	T7_1			171,26	184,95	165,4	168,9	152,98	134,56		178,13	191,54	174,03	168,42	146,76	132,64	
sigma = 4	T1_1			842,87	944,23	942,28	897,45	807,99	742,34		851,18	832,1	933,96	771,4	695,5	732,77	
	T2_1			665,77	669,67	753,83	635,02	636,08	554,34		516,13	657,04	615,35	546,81	582,67	547,84	
	T7_1			163,07	180,23	162,41	163,32	143,13	134,56		168,24	184,66	166,85	163,59	139,29	132,64	
sigma = 5	T1_1			799,52	905,02	910,07	857,84	762,39	742,34		806,51	808,03	898,02	735,16	664,7	732,77	
	T2_1			628,35	651,92	723,51	606,09	602,2	554,34		493,74	625,11	592,13	522,46	558,09	547,84	
	T7_1			154,45	174,19	158,91	156,79	132,86	134,56		157,4	176,96	159,09	158,23	131,21	132,64	
sigma = 6	T1_1			751,98	863,12	873,26	815,89	716,12	742,34		760,02	783,46	860,08	698,31	631,34	732,77	
	T2_1			590,08	632,59	689,75	574,82	565,18	554,34		469,83	593,01	567,7	495,79	532,65	547,84	
	T7_1			145,83	167,07	154,98	149,64	122,84	134,56		146,3	168,89	151,18	152,67	123,05	132,64	
Sigma = 7	T1_1			702,82	820,12	833,42	773,41	671,18	742,34		713,92	759,39	821,57	662,28	596,87	732,77	
	T2_1			552,62	611,72	654,05	542,51	526,88	554,34		445,55	562,37	542,97	468,07	507,14	547,84	
	T7_1			137,49	159,18	150,65	142,2	113,46	134,56		135,49	160,77	143,48	147,17	115,2	132,64	
Sigma = 8	T1_1			654,11	777,08	792,08	731,61	628,72	742,34		669,66	736,19	783,51	627,75	562,41	732,77	
	T2_1			516,95	589,28	617,67	510,16	488,82	554,34		421,79	534,14	518,53	440,26	481,92	547,84	
	T7_1			129,53	150,89	145,9	134,71	104,89	134,56		125,33	152,8	136,15	141,88	107,87	132,64	
Sigma = 9	T1_1			607,28	734,66	750,51	691,22	589,25	742,34		628,05	713,76	746,54	594,96	528,72	732,77	
	T2_1			483,54	565,38	581,61	478,47	452,14	554,34		399,11	508,68	494,75	413,09	457,17	547,84	
	T7_1			121,99	142,49	140,74	127,37	97,16	134,56		115,99	145,09	129,27	136,87	101,16	132,64	
Sigma = 12	T1_1			484,59	614,58	632,22	581,92	488,87	742,34		521,27	647,28	644,98	506,2	436,62	732,77	
	T2_1			397,72	488,43	480,88	391,34	356,36	554,34		339,81	446,9	428,67	339,02	386,49	547,84	
	T7_1			101,75	118,77	123,44	107,3	78,41	134,56		92,93	123,82	111,28	123,26	84,45	132,64	

Table S43 shows the activity concentrations measured using WKP and with 4 mL small VOI methods on SPECTs images post-filtered with Gaussian filter (0-12 mm) for patient number 7. Whereas, RC represented patient specific recovery coefficient of right and the left kidneys, respectively.

Post-filtering (mm)	Data	Time p.i. (h)	Inj Act (MBq)	Right Kidney							Left Kidney						
			7713	VOI_1 (counts/mL)	VOI_2 (counts/mL)	VOI_3 (counts/mL)	VOI_4 (counts/mL)	VOI_5 (counts/mL)	WKP (counts/mL)	RC	VOI_1 (counts/mL)	VOI_2 (counts/mL)	VOI_3 (counts/mL)	VOI_4 (counts/mL)	VOI_5 (counts/mL)	WKP (counts/mL)	RC
sigma = 0	T1_1	22,3		1161,03	954,75	1057,88	1068,41	1014,6	815,15	0,86	1153,69	1126,16	945,64	1136,24	1092,45	755,35	0,86
	T2_1	48,1		722,2	689,84	651,31	683,61	649,36	536,03		725,07	723,08	628,17	651,44	602,46	489,57	
	T7_1	170,2		140,54	159,17	131,1	128,64	121,08	108,46		144,26	144,46	137,44	130,58	167,31	109	
Sigma = 3	T1_1			1097,75	912,97	999,79	995,04	946,11	815,15		1087,09	1071,38	891,51	1079,81	1051,2	755,35	
	T2_1			681,66	652,76	612,78	633,16	603,53	536,03		688,3	665,56	584,42	621,78	573,15	489,57	
	T7_1			136,2	149,39	122,83	123,46	116,51	108,46		137,81	136,41	128,5	122,36	154,85	109	
sigma = 4	T1_1			1053,62	885,35	958,74	944,89	899,27	815,15		1040,11	1031,09	852,88	1039,4	1022,48	755,35	
	T2_1			653,45	628,52	586,25	599,26	572,42	536,03		660,89	628,34	554,63	602,05	550,5	489,57	
	T7_1			132,53	143,49	117,31	120,56	113,75	108,46		133,34	131,21	122,66	116,54	146,94	109	
sigma = 5	T1_1			1002,82	854,95	911,76	888,99	846,69	815,15		986,34	983,37	807,35	992,33	989,05	755,35	
	T2_1			621,46	602,1	556,29	561,86	537,87	536,03		628,35	588,52	521,39	580,28	522,85	489,57	
	T7_1			127,98	137,23	111,28	117,58	110,66	108,46		128,23	125,74	116,59	110,03	138,53	109	
sigma = 6	T1_1			947,86	823,03	861,5	830,65	791,27	815,15		928,82	930,71	757,1	940,75	951,99	755,35	
	T2_1			587,42	574,77	524,46	523,11	501,89	536,03		592,61	548,59	486,56	557,57	491,69	489,57	
	T7_1			122,86	130,8	105,02	114,55	107,26	108,46		122,71	120,28	110,75	103,24	130,11	109	
Sigma = 7	T1_1			891,37	790,49	810,29	772,61	735,62	815,15		870,4	836,72	704,82	798,07	860,91	755,35	
	T2_1			552,9	547,47	492,2	466,55	466,18	536,03		555,71	510,36	451,71	534,71	429,94	489,57	
	T7_1			117,44	124,41	98,77	111,48	103,59	108,46		117,08	114,96	105,37	96,5	122,02	109	
Sigma = 8	T1_1			835,49	757,84	759,85	716,64	681,58	815,15		813,34	790,14	652,89	745,24	820,29	755,35	
	T2_1			519,07	520,7	460,52	432,25	431,92	536,03		519,32	474,82	417,93	512,23	401,11	489,57	
	T7_1			112,01	118,19	92,69	108,37	99,65	108,46		111,63	109,86	100,49	90,05	114,5	109	
Sigma = 9	T1_1			781,82	725,41	711,25	663,77	630,4	815,15		759,31	745,01	603,08	659,88	780,41	755,35	
	T2_1			486,71	494,74	430,07	400,06	399,79	536,03		484,65	442,36	385,92	490,39	373,16	489,57	
	T7_1			106,76	112,22	86,88	105,23	95,52	108,46		106,63	105,04	96,13	84,1	107,67	109	
Sigma = 12	T1_1			643,16	631,31	581,47	527,63	499,3	815,15		624,09	626,29	475,63	568,89	669,54	755,35	
	T2_1			402,62	422,42	349,17	318,08	318,49	536,03		396,81	362,56	303,66	429,76	301,45	489,57	
	T7_1			93,7	96,04	71,49	96,46	82,87	108,46		95,9	93,08	86,22	70,62	91,95	109	

Table S44 shows the activity concentrations measured using WKP and with 4 mL small VOI methods on SPECTs images post-filtered with Gaussian filter (0-12 mm) for patient number 8. Whereas, RC represented patient specific recovery coefficient of right and the left kidneys, respectively.

Post-filtering (mm)	Data	Time p.i. (h)	Inj Act (MBq)	Right Kidney							Left Kidney						
			7690	VOI_1 (counts/mL)	VOI_2 (counts/mL)	VOI_3 (counts/mL)	VOI_4 (counts/mL)	VOI_5 (counts/mL)	WKP (counts/mL)	RC	VOI_1 (counts/mL)	VOI_2 (counts/mL)	VOI_3 (counts/mL)	VOI_4 (counts/mL)	VOI_5 (counts/mL)	WKP (counts/mL)	RC
sigma = 0	T1_1	19,5		974,8	885,99	916,97	897,09	868,63	691,31	0,87	1060,7	909,27	875,28	805,19	891,84	758,2	0,88
	T2_1	46,7		554,21	537,52	594,22	615,83	564,17	512,72		676,22	718,5	768,98	682,56	665,57	541,35	
	T7_1	169,1		152,25	133,2	138,96	127,68	134,54	99,5		154,18	125,23	132,8	130,89	150,03	96,43	
Sigma = 3	T1_1			914,46	861,46	893,57	867,05	839,91	691,31		998,87	861,46	833,21	767,19	839,6	758,2	
	T2_1			529,18	513,02	567,46	581,32	540,29	512,72		655,79	685,4	721,71	653,37	624,56	541,35	
	T7_1			142,04	131,18	131,21	122,6	128,61	99,5		142,67	120,16	124,62	121,8	143,55	96,43	
sigma = 4	T1_1			873,24	844,21	875,53	842,95	815,04	691,31		955,67	829,39	805,17	741,77	803,6	758,2	
	T2_1			511,42	497,14	551,39	558,02	521,76	512,72		637,95	662,24	690,11	633,36	596,76	541,35	
	T7_1			135,4	130,06	126,94	118,8	123,91	99,5		135,08	116,4	119,61	115,5	139,45	96,43	
sigma = 5	T1_1			827,26	824,26	853,2	813,14	783,1	691,31		906,81	794,25	774,14	713,45	762,5	758,2	
	T2_1			490,93	480,72	534,31	532,25	499	512,72		614,64	636,25	655,72	610,19	565,21	541,35	
	T7_1			128,27	128,99	123,04	114,25	118,1	99,5		126,84	111,99	114,39	108,41	134,86	96,43	
sigma = 6	T1_1			779,24	802,27	827,21	779,01	745,59	691,31		854,94	758,19	741,29	683,3	718,22	758,2	
	T2_1			468,9	465,21	516,92	505,07	473	512,72		586,98	609,16	620,57	584,85	531,36	541,35	
	T7_1			121,15	127,99	119,76	109,19	111,46	99,5		118,47	107,25	109,16	100,97	129,97	96,43	
Sigma = 7	T1_1			731,5	778,83	798,4	742,18	704,45	691,31		802,48	722,97	707,7	652,06	672,7	758,2	
	T2_1			446,55	451,46	499,68	477,32	444,98	512,72		556,57	582,4	586,15	558,4	496,68	541,35	
	T7_1			114,43	127,03	117,14	103,86	104,32	99,5		110,39	102,5	104,04	93,56	124,95	96,43	
Sigma = 8	T1_1			685,61	754,37	767,69	704,18	661,58	691,31		751,21	689,68	674,21	620,25	627,45	758,2	
	T2_1			424,84	439,72	482,69	449,62	416,16	512,72		525,01	556,88	553,3	531,64	462,38	541,35	
	T7_1			108,36	126,03	115,04	98,48	97,02	99,5		102,86	97,96	99,08	86,45	119,92	96,43	
Sigma = 9	T1_1			642,53	729,25	735,9	666,2	618,58	691,31		702,29	658,82	641,46	588,28	583,62	758,2	
	T2_1			404,36	429,73	465,87	422,43	387,58	512,72		493,61	533	522,47	505,12	429,36	541,35	
	T7_1			103,01	124,89	113,28	93,19	89,86	99,5		95,99	93,76	94,32	79,81	114,96	96,43	
Sigma = 12	T1_1			533,59	652,46	640,41	559,37	499,07	691,31		574,77	580,43	551,46	495,38	466,93	758,2	
	T2_1			352,73	405,45	415,66	347,09	309,75	512,72		407,81	470,58	442,86	429,89	342,7	541,35	
	T7_1			91,07	120,15	108,37	78,69	70,84	99,5		79,33	83,28	81,4	63,16	100,89	96,43	

Table S45 shows the activity concentrations measured using WKP and with 4 mL small VOI methods on SPECTs images post-filtered with Gaussian filter (0-12 mm) for patient number 9. Whereas, RC represented patient specific recovery coefficient of right and the left kidneys, respectively.

Post-filtering (mm)	Data	Time p.i. (h)	Inj Act (MBq)	Right Kidney							Left Kidney						
			7738	VOI_1 (counts/mL)	VOI_2 (counts/mL)	VOI_3 (counts/mL)	VOI_4 (counts/mL)	VOI_5 (counts/mL)	WKP (counts/mL)	RC	VOI_1 (counts/mL)	VOI_2 (counts/mL)	VOI_3 (counts/mL)	VOI_4 (counts/mL)	VOI_5 (counts/mL)	WKP (counts/mL)	RC
sigma = 0	T0_1	4,4		808,48	818,64	796,63	686,75	614,23	530,54	0,86	687,25	772,23	719,94	720,49	585,29	489,49	0,87
	T2_1	51,0		346,32	434,78	394,35	350,39	304,07	279,86		411,18	364,98	383,4	318,72	271,68	249,22	
	T7_1	171,3		101,7	76,92	89,16	97,63	99,96	55,15		109,88	107,8	85,08	85,08	76,42	52,24	
Sigma = 3	T0_1			760,95	771,06	757,42	654,77	585,15	530,54		659,78	726,69	670,4	678,24	556,42	489,49	
	T2_1			326,12	409,65	375,89	340,27	291,48	279,86		375,39	351,12	360,87	297,58	261,56	249,22	
	T7_1			93,25	72,8	86,74	91,34	96,69	55,15		100,62	95,44	81,2	76,96	68,84	52,24	
sigma = 4	T0_1			729,45	739,64	729,83	630,93	564,93	530,54		639,16	696,36	638,41	649,55	536,59	489,49	
	T2_1			313,21	393,21	364,05	332,01	282,56	279,86		352,26	340,99	346,09	283,93	253,65	249,22	
	T7_1			87,69	70,05	84,66	87,45	94,43	55,15		94,33	88,03	78,26	71,92	64,13	52,24	
sigma = 5	T0_1			695,29	705,84	698,6	602,61	541,98	530,54		614,48	663,19	604,08	617,92	514	489,49	
	T2_1			299,45	375,35	351,33	321,37	272,07	279,86		327,86	329,06	330,06	269,26	243,87	249,22	
	T7_1			81,69	67,07	82	83,38	91,9	55,15		87,45	80,62	74,72	66,9	59,28	52,24	
sigma = 6	T0_1			660,79	671,86	665,61	571,45	517,42	530,54		586,92	628,95	569,52	585,21	489,62	489,49	
	T2_1			285,66	357,09	338,25	308,87	260,43	279,86		303,76	315,84	313,68	254,48	232,56	249,22	
	T7_1			75,62	64,01	78,9	79,35	89,28	55,15		80,45	73,68	70,77	62,29	54,62	52,24	
Sigma = 7	T1_1			627,86	639,22	632,51	539,11	492,29	530,54		557,76	595,01	536,32	552,87	464,36	489,49	
	T2_1			272,52	339,25	325,17	295,16	248,04	279,86		281,08	301,86	297,54	240,18	220,21	249,22	
	T7_1			69,76	60,96	75,57	75,53	86,73	55,15		73,73	67,48	66,65	58,25	50,33	52,24	
Sigma = 8	T1_1			597,83	608,69	600,49	506,92	467,33	530,54		528,11	562,19	505,28	521,76	438,95	489,49	
	T2_1			260,49	322,37	312,25	280,86	235,3	279,86		260,37	287,6	281,99	226,67	207,35	249,22	
	T7_1			64,26	57,94	72,17	72,02	84,35	55,15		67,53	62,06	62,55	54,79	46,51	52,24	
Sigma = 9	T1_1			571,47	580,58	570,2	475,79	443,03	530,54		498,83	530,91	476,66	492,32	413,95	489,49	
	T2_1			249,83	306,78	299,55	266,51	222,55	279,86		241,86	273,45	267,19	214,03	194,46	249,22	
	T7_1			59,19	54,97	68,83	68,88	82,18	55,15		61,97	61,97	57,38	58,62	51,82	52,24	
Sigma = 12	T1_1			515,42	510,74	491,43	392,87	376,47	530,54		418,79	447,55	404,16	414,95	344,84	489,49	
	T2_1			226,29	268,74	263,38	226,33	186,59	279,86		198,88	234,16	227,64	181,11	158,99	249,22	
	T7_1			46,71	46,5	59,8	61,63	76,66	55,15		49,17	46,72	48,34	44,7	35,11	52,24	

Table S46 shows the activity concentrations measured using WKP and with 4 mL small VOI methods on SPECTs images post-filtered with Gaussian filter (0-12 mm) for patient number 9. Whereas, RC represented patient specific recovery coefficient of right and the left kidneys, respectively.

Post-filtering (mm)	Data	Time p.i. (h)	Inj Act (MBq)	Right Kidney									Left Kidney					
			7738	VOI_1 (counts/mL)	VOI_2 (counts/mL)	VOI_3 (counts/mL)	VOI_4 (counts/mL)	VOI_5 (counts/mL)	WKP (counts/mL)	RC	VOI_1 (counts/mL)	VOI_2 (counts/mL)	VOI_3 (counts/mL)	VOI_4 (counts/mL)	VOI_5 (counts/mL)	WKP (counts/mL)	RC
sigma = 0	T1_1	22,2		811,67	769,86	876,27	938,14	1000,11	670,81	0,83	854,05	1000,6	853,61	640,58	797,37	717,2	0,82
	T2_1	119,1		156,35	164,42	149,26	118,72	150,99	126,71		174,13	147,33	141,24	163,87	149,04	123,39	
	T7_1	173,2		80,18	75,63	99,01	70,73	65,25	58,98		98,11	84,35	92,74	109,24	87,39	60,76	
Sigma = 3	T1_1			753,41	730,61	821,38	896,71	944,06	670,81		810,22	944,71	799,34	602,57	735,96	717,2	
	T2_1			145,13	158,42	140,63	112,68	139,53	126,71		159,4	140,84	131,41	151,88	139,35	123,39	
	T7_1			77,61	71,04	89,76	64,93	60,45	58,98		87,92	77,73	85,7	104,03	80,61	60,76	
sigma = 4	T1_1			714,94	702,66	784,17	867,53	906,12	670,81		779,82	907,96	764,04	576,82	694,95	717,2	
	T2_1			137,93	153,89	134,61	109,12	132,51	126,71		149,57	135,63	125,02	143,95	133,37	123,39	
	T7_1			75,52	68,29	83,93	61,4	57,26	58,98		81,75	73,36	81,19	100,38	76,37	60,76	
sigma = 5	T1_1			672,87	670,98	742,95	833,49	863,83	670,81		745,59	868,81	725,08	548,22	650,61	717,2	
	T2_1			130,12	148,39	127,9	105,41	125,23	126,71		139,11	129,57	118,36	135,46	127,25	123,39	
	T7_1			73,04	65,39	77,9	57,68	53,94	58,98		75,6	68,75	76,48	96,41	72,01	60,76	
sigma = 6	T1_1			629,6	637,64	700,14	796,32	819,67	670,81		709,24	829,67	684,59	518,5	606,06	717,2	
	T2_1			122,13	142,2	120,87	101,61	118,12	126,71		128,81	123,09	111,83	126,97	121,4	123,39	
	T7_1			70,33	62,46	72,03	55,98	50,73	58,98		69,89	64,18	71,85	92,37	67,79	60,76	
Sigma = 7	T1_1			587,08	604,28	657,74	757,58	775,73	670,81		672,32	792,13	644,57	489,05	563,69	717,2	
	T2_1			114,34	135,61	113,87	97,79	111,48	126,71		119,22	116,54	105,69	118,95	116,06	123,39	
	T7_1			67,54	59,6	66,56	50,54	47,75	58,98		64,86	59,86	67,48	88,42	63,89	60,76	
Sigma = 8	T1_1			546,53	572,08	617,02	718,45	733,33	670,81		635,89	756,96	606,41	460,77	524,87	717,2	
	T2_1			106,97	128,89	107,09	93,96	105,41	126,71		110,61	110,16	100,05	111,65	111,31	123,39	
	T7_1			64,76	56,88	61,59	47,17	45,05	58,98		60,6	55,88	63,45	84,66	60,37	60,76	
Sigma = 9	T1_1			508,6	541,76	578,72	679,8	693,16	670,81		600,6	724,28	570,89	434,15	490,18	717,2	
	T2_1			100,14	122,26	100,69	90,15	99,95	126,71		103,07	104,09	94,91	105,16	107,13	123,39	
	T7_1			62,05	54,31	57,13	44,15	42,63	58,98		57,05	52,29	59,77	81,1	57,25	60,76	
Sigma = 12	T1_1			411,86	464,22	479,96	571,59	587,16	670,81		504,43	638,38	481,66	365,22	409,43	717,2	
	T2_1			83,03	103,91	84,21	79,07	86,76	126,71		85,96	88,02	82,31	90,19	97,19	123,39	
	T7_1			54,42	47,49	46,49	36,58	36,77	58,98		49,69	43,73	50,56	71,56	49,97	60,76	

Table S47 shows the activity concentrations measured using WKP and with 4 mL small VOI methods on SPECTs images post-filtered with Gaussian filter (0-12 mm) for patient number 11. Whereas, RC represented patient specific recovery coefficient of right and the left kidneys, respectively.

Post-filtering (mm)	Data	Time p.i. (h)	Inj Act (MBq)	Right Kidney							Left Kidney						
			7736	VOI_1 (counts/mL)	VOI_2 (counts/mL)	VOI_3 (counts/mL)	VOI_4 (counts/mL)	VOI_5 (counts/mL)	WKP (counts/mL)	RC	VOI_1 (counts/mL)	VOI_2 (counts/mL)	VOI_3 (counts/mL)	VOI_4 (counts/mL)	VOI_5 (counts/mL)	WKP (counts/mL)	RC
sigma = 0	T1_1	22,7		728,68	767,35	819,37	694,33	708,31	609,22	0,87	632,37	683,46	690,97	642,46	779,82	578,46	0,86
	T2_1	119,7		155,15	166,4	177,07	180,44	159,99	120,09		127,38	116,31	163,11	148,7	180,93	120,57	
	T7_1	172,1		97,54	99,15	101,93	105,29	81,62	69,89		94,05	90,71	99,6	103,88	99,92	66,14	
Sigma = 3	T1_1			684,01	723,49	774,18	668,53	674,17	609,22		591,13	636,66	653,57	608,15	714,45	578,46	
	T2_1			144,34	157,33	168,13	175,89	148,66	120,09		122,8	109,93	152,99	140,8	166,8	120,57	
	T7_1			91,46	94,19	92,51	98,25	81,2	69,89		86,79	85,59	95,23	95,19	95,74	66,14	
sigma = 4	T1_1			653,47	696,87	744,98	651	650,34	609,22		563,98	606,33	629,12	583,4	672	578,46	
	T2_1			137,15	151,45	162,77	172,07	141,59	120,09		119,37	106,38	146,39	135,55	158,4	120,57	
	T7_1			87,43	91,08	86,64	93,54	80,8	69,89		82	81,97	92,4	89,6	93,16	66,14	
sigma = 5	T1_1			618,97	669,82	713,67	631,56	622,82	609,22		534,42	574,3	602,78	554,43	626,39	578,46	
	T2_1			129,36	145,09	157,29	167,23	134,23	120,09		115,4	103,09	139,3	129,8	149,96	120,57	
	T7_1			83,05	87,85	80,6	88,31	80,23	69,89		76,86	77,76	89,28	83,74	90,52	66,14	
sigma = 6	T1_1			582,39	643,5	682	611,41	592,79	609,22		503,99	543	575,91	522,72	580,56	578,46	
	T2_1			121,41	138,55	151,82	161,62	126,97	120,09		111,11	100,31	132,19	123,77	141,93	120,57	
	T7_1			78,61	84,71	74,75	82,8	79,46	69,89		71,74	73,19	86,02	77,97	87,99	66,14	
Sigma = 7	T1_1			545,43	618,26	651,22	591,4	561,54	609,22		473,91	514,15	549,4	489,82	536,8	578,46	
	T2_1			113,61	132,09	146,41	155,52	120,08	120,09		106,65	98,15	125,43	117,64	134,48	120,57	
	T7_1			74,3	81,81	69,34	77,24	78,44	69,89		66,91	68,49	82,71	72,5	85,64	66,14	
Sigma = 8	T1_1			509,34	593,88	621,92	571,86	530,17	609,22		444,94	488,55	523,62	457,02	496,4	578,46	
	T2_1			106,13	125,89	141,1	149,18	113,67	120,09		102,17	96,54	119,22	111,53	127,6	120,57	
	T7_1			70,28	79,19	64,49	71,76	77,14	69,89		62,53	63,85	79,43	67,43	83,48	66,14	
Sigma = 9	T1_1			474,96	569,99	594,26	552,77	499,53	609,22		417,54	466,31	498,62	425,26	459,89	578,46	
	T2_1			99,07	120,03	135,91	142,8	107,76	120,09		97,75	95,34	113,66	105,53	121,17	120,57	
	T7_1			66,59	76,84	60,24	66,5	75,59	69,89		58,66	59,42	76,2	62,77	81,43	66,14	
Sigma = 12	T1_1			385,48	499,31	520,01	496,45	416,73	609,22		346,48	415,47	427,79	341,36	373,05	578,46	
	T2_1			80,93	104,56	121,29	124,73	92,67	120,09		85,55	92,72	100,52	88,72	103,65	120,57	
	T7_1			57,52	71,01	50,71	52,78	69,86	69,89		50,07	47,96	67,15	51,15	75,43	66,14	

Table S48 shows the activity concentrations measured using WKP and with 4 mL small VOI methods on SPECTs images post-filtered with Gaussian filter (0-12 mm) for patient number 12. Whereas, RC represented patient specific recovery coefficient of right and the left kidneys, respectively.

Post-filtering (mm)	Data	Time p.i. (h)	Inj Act (MBq)	Right Kidney										Left Kidney			
			7616	VOI_1 (counts/mL)	VOI_2 (counts/mL)	VOI_3 (counts/mL)	VOI_4 (counts/mL)	VOI_5 (counts/mL)	WKP (counts/mL)	RC	VOI_1 (counts/mL)	VOI_2 (counts/mL)	VOI_3 (counts/mL)	VOI_4 (counts/mL)	VOI_5 (counts/mL)	WKP (counts/mL)	RC
sigma = 0	T1_1	22,9		570,52	515,91	583,1	507,35	646,67	409,6	0,85	614,94	643,24	681,31	611,84	536,74	418,29	0,84
	T2_1	50,1		317,1	383,33	379,54	365,14	332,43	260,19		382	306,96	426,38	427,62	391,2	251,38	
	T7_1	173,0		53,57	49,84	51,19	61,96	63,29	35,25		55,64	56,31	59,41	54,66	52,07	34,67	
Sigma = 3	T1_1			525,33	501,96	551,21	479,47	609,44	409,6		575,58	597,8	629,83	571,55	503,66	418,29	
	T2_1			296,57	366,02	353,45	346,66	315,77	260,19		359,04	293,16	393,77	390,38	370,9	251,38	
	T7_1			49,31	48,15	48,85	57,16	57,71	35,25		51,87	54,07	56,59	51,14	50,14	34,67	
sigma = 4	T1_1			495,71	491,41	529,54	460,52	583,28	409,6		550,02	569,4	596,73	544,03	480,6	418,29	
	T2_1			282,57	353,43	336,1	333,95	304,11	260,19		342,79	283,57	373,51	367,18	356,39	251,38	
	T7_1			46,58	46,97	47,23	54,14	54,21	35,25		49,38	52,4	54,47	48,74	48,62	34,67	
sigma = 5	T1_1			464,14	478,53	505,29	439,49	553,68	409,6		522,48	539,28	561,45	513,46	454,43	418,29	
	T2_1			266,88	338,75	317,35	319,54	290,92	260,19		324,3	272,59	352,19	342,92	339,9	251,38	
	T7_1			43,7	45,63	45,41	50,95	50,56	35,25		46,65	50,42	51,94	46,09	46,79	34,67	
sigma = 6	T1_1			432,92	463,82	479,81	417,71	522,35	409,6		494,39	508,88	525,99	481,74	426,71	418,29	
	T2_1			250,53	322,8	298,36	304,16	277,07	260,19		304,74	260,66	330,86	318,96	322,47	251,38	
	T7_1			40,89	44,19	43,5	47,79	46,97	35,25		43,86	48,25	49,17	43,32	44,82	34,67	
Sigma = 7	T1_1			403,71	447,87	454,33	396,22	490,74	409,6		466,76	479,22	491,76	450,44	398,97	418,29	
	T2_1			234,5	306,29	279,91	288,52	263,29	260,19		285,15	248,14	310,29	296,27	304,97	251,38	
	T7_1			38,29	42,68	41,57	44,78	43,59	35,25		41,15	46	46,31	40,57	42,82	34,67	
Sigma = 8	T1_1			377,39	431,17	429,7	375,69	459,84	409,6		440,18	450,83	459,54	420,54	372,3	418,29	
	T2_1			219,51	289,74	262,4	273,09	250,02	260,19		266,24	235,32	290,9	275,34	287,99	251,38	
	T7_1			35,95	41,12	39,65	41,98	40,51	35,25		38,62	43,76	43,5	37,92	40,88	34,67	
Sigma = 9	T1_1			354,3	414,13	406,38	356,48	430,27	409,6		414,99	423,98	429,65	392,55	347,38	418,29	
	T2_1			205,98	273,48	246,03	258,2	237,48	260,19		248,44	222,45	272,86	256,31	271,91	251,38	
	T7_1			33,89	39,53	37,78	39,4	37,74	35,25		36,3	41,56	40,81	35,41	39,04	34,67	
Sigma = 12	T1_1			302,49	363,28	345,34	306,84	352,25	409,6		348,96	353,37	353,78	320,82	285,23	418,29	
	T2_1			174,96	228,24	203,84	217,92	204,24	260,19		203,21	185,52	226,98	209,6	230,13	251,38	
	T7_1			29,13	34,62	32,46	32,82	31,19	35,25		30,78	35,53	33,75	28,9	34,13	34,67	

Table S49 shows the activity concentrations measured using WKP and with 4 mL small VOI methods on SPECTs images post-filtered with Gaussian filter (0-12 mm) for patient number 13. Whereas, RC represented patient specific recovery coefficient of right and the left kidneys, respectively.

Post-filtering (mm)	Data	Time p.i. (h)	Inj Act (MBq)	Right Kidney							Left Kidney						
			7589	VOI_1 (counts/mL)	VOI_2 (counts/mL)	VOI_3 (counts/mL)	VOI_4 (counts/mL)	VOI_5 (counts/mL)	WKP (counts/mL)	RC	VOI_1 (counts/mL)	VOI_2 (counts/mL)	VOI_3 (counts/mL)	VOI_4 (counts/mL)	VOI_5 (counts/mL)	WKP (counts/mL)	RC
sigma = 0	T1_1	22,9		515,11	554,49	540,54	521,77	552,54	353,04	0,85	445,5	489,98	523,73	495,11	486,73	330,61	0,84
	T2_1	50,3		383,66	411,91	443,9	367,55	416,76	290,42		422,42	411,63	410,55	428,49	404,09	259,69	
	T7_1	171,8		143,62	136,69	132,81	139,73	134,55	88,37		126,01	150,72	137,73	137,14	119,06	86,14	
Sigma = 3	T1_1			493,5	525,64	506,48	480,46	510,62	353,04		421,13	458,78	498,73	463,79	461,35	330,61	
	T2_1			374,11	389,07	412,12	352,74	381,12	290,42		393,09	375,78	377,37	395,72	380,48	259,69	
	T7_1			130,57	125,09	121,12	127,7	123,76	88,37		113,95	137,15	127,13	127,81	111,84	86,14	
sigma = 4	T1_1			477,08	504,4	481,96	453,68	483,66	353,04		403,93	437,49	481,1	443,57	444,45	330,61	
	T2_1			364,14	374,05	391,4	342,15	358,3	290,42		373,86	352,47	356,86	374,39	364,3	259,69	
	T7_1			122,37	117,89	113,79	120,01	116,79	88,37		106,48	128,47	120,66	121,88	107,09	86,14	
sigma = 5	T1_1			457,35	479,7	454,14	424,77	454,78	353,04		384,14	413,7	461,1	421,72	425,73	330,61	
	T2_1			350,4	357,23	368,71	329,25	334,25	290,42		352,92	327,64	335,06	351,33	346,7	259,69	
	T7_1			113,73	110,4	106,11	111,84	109,3	88,37		98,92	119,37	114,02	115,45	101,84	86,14	
sigma = 6	T1_1			435,24	452,97	424,77	395,41	425,53	353,04		362,73	388,65	440,08	399,17	406,02	330,61	
	T2_1			333,56	339,36	345,39	314,38	310,41	290,42		331,66	302,9	313,06	327,89	328,62	259,69	
	T7_1			105,18	103,07	98,54	103,67	101,66	88,37		91,77	110,5	107,53	108,82	96,33	86,14	
Sigma = 7	T1_1			411,81	425,52	395,41	366,97	397,07	353,04		340,62	363,48	419,24	376,6	386	330,61	
	T2_1			314,58	321,12	322,51	298,12	287,78	290,42		311,17	279,39	291,72	305,21	310,66	259,69	
	T7_1			97,11	96,14	91,38	95,81	94,18	88,37		85,23	102,33	101,38	102,27	90,77	86,14	
Sigma = 8	T1_1			387,98	398,38	367,19	340,34	370,02	353,04		318,5	339,02	399,56	354,41	366,2	330,61	
	T2_1			294,48	303,04	300,76	281,09	266,87	290,42		292,18	257,63	271,53	284,04	293,14	259,69	
	T7_1			89,73	89,7	84,8	88,42	87,04	88,37		79,34	95,1	95,61	95,95	85,34	86,14	
Sigma = 9	T1_1			364,44	372,24	340,84	315,98	344,69	353,04		296,89	315,83	381,69	332,91	347,06	330,61	
	T2_1			274,17	285,46	280,45	263,88	247,89	290,42		275,07	237,76	252,7	264,82	276,26	259,69	
	T7_1			83,11	83,75	78,85	81,58	80,36	88,37		74,02	88,9	90,2	89,94	80,14	86,14	
Sigma = 12	T1_1			299,79	302,75	275,38	257,16	279,2	353,04		238,38	256,18	341,64	274,61	296,96	330,61	
	T2_1			218,17	237,67	228,52	215,28	202,21	290,42		235,52	188,43	204,79	220,36	230,43	259,69	
	T7_1			67,37	68,47	64,51	64,42	63,49	88,37		60,54	75,83	75,88	73,95	66,3	86,14	

Table S50 shows the activity concentrations measured using WKP and with 4 mL small VOI methods on SPECTs images post-filtered with Gaussian filter (0-12 mm) for patient number 14. Whereas, RC represented patient specific recovery coefficient of right and the left kidneys, respectively.

Post-filtering (mm)	Data	Time p.i. (h)	Inj Act (MBq)	Right Kidney							Left Kidney						
			7 614	VOI_1 (counts/mL)	VOI_2 (counts/mL)	VOI_3 (counts/mL)	VOI_4 (counts/mL)	VOI_5 (counts/mL)	WKP (counts/mL)	RC	VOI_1 (counts/mL)	VOI_2 (counts/mL)	VOI_3 (counts/mL)	VOI_4 (counts/mL)	VOI_5 (counts/mL)	WKP (counts/mL)	RC
sigma = 0	T1_1	24,5		712,62	654,96	697,89	657,64	565,22	486,91	0,87	731,57	687,77	634,71	598,86	563,86	470	0,83
	T2_1	51,2		439,89	450,65	486,23	506,66	446,97	346,22		488,71	392	399,92	442,36	414,2	316,25	
	T7_1	172,5		103,2	86,36	88,39	77,96	86,35	57,37		106,44	95,57	78,52	91,21	82,6	55,8	
Sigma = 3	T1_1			673,59	623,13	663,99	634,85	538,35	486,91		692,73	645,55	586,12	563,11	530,83	470	
	T2_1			420,63	428,95	465,78	475,94	422,28	346,22		459,32	377,73	377,86	418,72	388,55	316,25	
	T7_1			94,91	79,22	82,04	73,03	82,47	57,37		98,34	85,4	73,86	84,13	76,59	55,8	
sigma = 4	T1_1			646,03	607,27	641,99	616,82	519,22	486,91		665,45	617,68	555,56	536,92	511,1	470	
	T2_1			405,74	415,66	451,59	455,45	405,13	346,22		439,2	367,9	363,3	401,25	370,45	316,25	
	T7_1			89,4	74,96	78,22	69,81	79,59	57,37		93,22	78,97	70,8	79,44	72,51	55,8	
sigma = 5	T1_1			614,27	589,39	618,17	594,38	496,97	486,91		633,82	587,33	523,44	506,28	491,4	470	
	T2_1			387,74	401,6	435,47	432,78	385,5	346,22		416,5	356,84	347,36	380,53	349,63	316,25	
	T7_1			83,35	70,76	74,2	66,25	76,12	57,37		87,57	72,41	67,59	74,31	67,84	55,8	
sigma = 6	T1_1			579,94	570,2	593,61	568,59	472,73	486,91		599,16	556,03	491,23	472,99	472,87	470	
	T2_1			367,48	387,3	418,07	409,01	364,31	346,22		392,44	345,02	330,87	357,66	327,28	316,25	
	T7_1			77,15	66,9	70,09	62,48	72,25	57,37		81,68	66,07	64,46	69,05	62,87	55,8	
Sigma = 7	T1_1			544,67	550,08	568,96	540,72	447,6	486,91		562,91	524,87	459,87	438,85	455,82	470	
	T2_1			345,97	373,01	399,96	385,03	342,47	346,22		368,14	332,77	314,45	333,85	304,52	316,25	
	T7_1			71,11	63,5	66	58,64	68,15	57,37		75,84	60,17	61,51	63,9	57,9	55,8	
Sigma = 8	T1_1			509,73	529,14	544,41	511,87	422,43	486,91		526,28	494,49	429,76	405,32	439,93	470	
	T2_1			324,12	358,71	381,52	361,39	320,71	346,22		344,36	320,18	298,39	310,11	282,25	316,25	
	T7_1			65,43	60,55	61,97	54,84	63,98	57,37		70,26	54,77	58,77	59,06	53,14	55,8	
Sigma = 9	T1_1			476,04	507,38	519,95	482,95	397,84	486,91		490,27	465,23	401,08	373,42	424,61	470	
	T2_1			302,65	344,29	363,02	338,45	299,57	346,22		321,56	307,26	282,82	287,19	261,08	316,25	
	T7_1			60,23	57,95	58,04	51,17	59,87	57,37		65,07	49,9	56,23	54,6	48,7	55,8	
Sigma = 12	T1_1			386,73	438,33	446,69	400,88	330,77	486,91		392,13	385,15	324,42	291,98	377,28	470	
	T2_1			244,74	299,64	308,59	275,59	242,77	346,22		260,84	266,68	239,16	227,09	206,95	316,25	
	T7_1			47,63	51,15	47,17	41,45	48,58	57,37		52,02	38,31	49,25	43,67	37,81	55,8	

Table S51 shows the activity concentrations measured using WKP and with 4 mL small VOI methods on SPECTs images post-filtered with Gaussian filter (0-12 mm) for patient number 15. Whereas, RC represented patient specific recovery coefficient of right and the left kidneys, respectively.

Post-filtering (mm)	Data	Time p.i. (h)	Inj Act (MBq)	Right Kidney							Left Kidney						
			7 620	VOI_1 (counts/mL)	VOI_2 (counts/mL)	VOI_3 (counts/mL)	VOI_4 (counts/mL)	VOI_5 (counts/mL)	WKP (counts/mL)	RC	VOI_1 (counts/mL)	VOI_2 (counts/mL)	VOI_3 (counts/mL)	VOI_4 (counts/mL)	VOI_5 (counts/mL)	WKP (counts/mL)	RC
sigma = 0	T1_1	20,7		507,9	574,58	508,82	456,73	516,2	419,72	0,85	495,19	520,86	601,71	617,68	535,63	426,56	0,84
	T2_1	48,4		392,84	390,07	384,04	328	319,38	243,98		365,37	355,93	362,75	306,47	290,23	235,34	
	T7_1	169,1		67,68	73,35	59,53	51,66	70,87	41,39		48,24	60,05	62,03	69,5	62,09	42,58	
Sigma = 3	T1_1			470,79	539,69	485,54	440,61	488,63	419,72		457,63	488,77	567,93	583,88	504,95	426,56	
	T2_1			361,84	363,92	353,28	302,71	305	243,98		341,07	329,27	346,72	287,24	271,05	235,34	
	T7_1			62,79	64,16	56,83	48,43	65,82	41,39		46,28	56,35	58,41	63,69	57,68	42,58	
sigma = 4	T1_1			446,95	516,33	469,31	428,64	471,37	419,72		432,91	468,14	545,07	560,45	484,04	426,56	
	T2_1			341,23	346,59	333,86	286,26	295,54	243,98		324,91	312,61	334,91	275,12	258,55	235,34	
	T7_1			59,52	58,65	54,49	46,26	62,28	41,39		44,67	53,92	55,85	59,9	54,67	42,58	
sigma = 5	T1_1			421,43	490,73	450,99	414,25	453,07	419,72		406,37	446,24	519,62	533,97	460,17	426,56	
	T2_1			318,61	327,58	313,52	268,61	285,04	243,98		307,12	295,21	321,13	262,49	244,9	235,34	
	T7_1			55,9	53,17	51,54	43,92	58,37	41,39		42,59	51,22	52,98	55,91	51,41	42,58	
sigma = 6	T1_1			395,55	464,37	431,44	398,04	434,7	419,72		379,7	424,3	492,91	505,76	436,14	426,56	
	T2_1			295,31	307,9	293,31	250,82	273,9	243,98		288,64	278,05	306,2	250,02	230,85	235,34	
	T7_1			52,15	48,08	48,23	41,57	54,37	41,39		40,2	48,36	49,97	52	48,1	42,58	
Sigma = 7	T1_1			370,21	438,41	411,35	380,6	416,9	419,72		354,15	403,18	466,07	477,07	411,2	426,56	
	T2_1			272,46	288,35	273,97	233,67	262,41	243,98		270,22	261,79	290,85	238,07	216,99	235,34	
	T7_1			48,44	43,56	44,79	39,28	50,49	41,39		37,66	45,49	46,97	48,33	44,89	42,58	
Sigma = 8	T1_1			345,92	413,52	391,24	362,48	399,86	419,72		330,42	383,27	439,87	448,84	386,48	426,56	
	T2_1			250,76	269,51	255,81	217,57	250,77	243,98		252,35	246,71	275,62	226,66	203,64	235,34	
	T7_1			44,91	39,63	41,42	37,13	46,88	41,39		35,11	42,68	44,1	44,97	41,86	42,58	
Sigma = 9	T1_1			322,94	390,02	371,53	344,14	383,56	419,72		308,78	364,63	414,76	421,68	362,42	426,56	
	T2_1			230,64	251,71	238,96	202,71	239,11	243,98		235,31	232,85	260,78	215,72	190,99	235,34	
	T7_1			41,61	36,23	38,23	35,16	43,59	41,39		32,64	39,99	41,42	41,93	39,04	42,58	
Sigma = 12	T1_1			262,78	327,73	317,31	291,24	337,25	419,72		255,72	315,28	347,96	349,03	296,58	426,56	
	T2_1			180,76	205,76	195,92	165,32	205	243,98		190,48	197,81	219,8	184,84	157,73	235,34	
	T7_1			33,39	28,52	30,15	30,26	35,58	41,39		26,22	32,83	34,63	34,61	31,82	42,58	

Table S52 shows the activity concentrations measured using WKP and with 4 mL small VOI methods on SPECTs images post-filtered with Gaussian filter (0-12 mm) for patient number 16. Whereas, RC represented patient specific recovery coefficient of right and the left kidneys, respectively.

Post-filtering (mm)	Data	Time p.i. (h)	Inj Act (MBq)	Right Kidney								Left Kidney				
			7 488	VOI_1 (counts/mL)	VOI_2 (counts/mL)	VOI_3 (counts/mL)	VOI_4 (counts/mL)	VOI_5 (counts/mL)	WKP (counts/mL)	RC	VOI_1 (counts/mL)	VOI_2 (counts/mL)	VOI_3 (counts/mL)	VOI_4 (counts/mL)	VOI_5 (counts/mL)	WKP (counts/mL)	RC
sigma = 0	T1_1	23,2		634,48	547,13	547,23	582,03	545,86	440,12	0,86	571,09	556,36	555,04	625,61	641	410,14	0,85
	T2_1	50,2		364,87	379,42	409,56	370,54	343,84	235,88		401,53	337,05	355,76	389,21	383,86	254,2	
	T7_1	171,3		70,64	81	70,3	69,34	65,76	45,27		58,49	65,35	69,23	84,2	66,13	44,04	
Sigma = 3	T1_1			597,63	530	529,35	557,73	527,67	440,12		535,51	518,89	524,5	566,2	600,95	410,14	
	T2_1			347,1	350,91	391,54	353,03	316,9	235,88		355,95	312,27	337,67	363,06	362,34	254,2	
	T7_1			67,64	75,15	65,26	63,23	62,89	45,27		53,61	58,99	65,23	78,04	60,02	44,04	
sigma = 4	T1_1			573,07	518,49	515,88	539,29	515,4	440,12		511,03	495,11	503,06	527,28	574,38	410,14	
	T2_1			334,94	332,56	377,99	341,38	299,45	235,88		327,49	296,36	325,34	345,96	347,6	254,2	
	T7_1			65,53	71,15	62,42	59,4	61,17	45,27		50,35	55,11	62,77	74,05	56,23	44,04	
sigma = 5	T1_1			545,83	505,34	499,63	517,27	501,42	440,12		483,6	470,37	478,25	485,38	544,88	410,14	
	T2_1			321,24	312,95	361,69	328,58	280,81	235,88		298,11	279,62	311,29	327,32	330,78	254,2	
	T7_1			62,97	66,85	59,47	55,5	59,35	45,27		46,79	51,3	60,2	69,77	52,31	44,04	
sigma = 6	T1_1			517,51	491,11	481,41	492,71	486,09	440,12		454,92	446,03	451,56	443,15	513,89	410,14	
	T2_1			306,83	293,33	343,43	315,22	262,17	235,88		269,75	263,04	296,08	308,25	312,78	254,2	
	T7_1			60,09	62,56	56,47	51,81	57,47	45,27		43,15	47,79	57,63	65,43	48,49	44,04	
Sigma = 7	T1_1			489,48	476,27	462,1	466,69	469,52	440,12		426,38	422,9	424,43	402,77	482,83	410,14	
	T2_1			292,51	274,66	324,17	301,63	244,45	235,88		243,63	247,2	280,3	289,62	294,5	254,2	
	T7_1			57,02	58,48	53,45	48,49	55,57	45,27		39,63	44,64	55,07	61,22	44,94	44,04	
Sigma = 8	T1_1			462,76	461,07	442,44	440,18	451,73	440,12		398,91	401,22	397,91	365,59	452,72	410,14	
	T2_1			278,81	257,44	304,73	287,98	228,2	235,88		220,32	232,33	264,41	271,91	276,58	254,2	
	T7_1			53,92	54,67	50,44	45,61	53,69	45,27		36,37	41,84	52,53	57,26	41,71	44,04	
Sigma = 9	T1_1			437,93	445,67	423,05	414,01	432,81	440,12		373	380,9	372,64	332,3	424,16	410,14	
	T2_1			266	241,85	285,73	274,34	213,71	235,88		199,95	218,48	248,8	255,38	259,41	254,2	
	T7_1			50,88	51,16	47,46	43,14	51,83	45,27		33,45	39,34	50	53,59	38,81	44,04	
Sigma = 12	T1_1			376,04	399,04	369,58	342,34	371,85	440,12		305,95	326,03	306,39	256,19	349,9	410,14	
	T2_1			233,06	203,93	234,5	234,31	180,8	235,88		154,88	182,65	205,75	213,17	213,93	254,2	
	T7_1			42,68	42,24	39,04	37,69	46,33	45,27		27,03	33,2	42,51	44,27	31,88	44,04	

Table S53 shows the activity concentrations measured using WKP and with 4 mL small VOI methods on SPECTs images post-filtered with Gaussian filter (0-12 mm) for patient number 17. Whereas, RC represented patient specific recovery coefficient of right and the left kidneys, respectively.

Post-filtering (mm)	Data	Time p.i. (h)	Inj Act (MBq)	Right Kidney							Left Kidney						
			7584	VOI_1 (counts/mL)	VOI_2 (counts/mL)	VOI_3 (counts/mL)	VOI_4 (counts/mL)	VOI_5 (counts/mL)	WKP (counts/mL)	RC	VOI_1 (counts/mL)	VOI_2 (counts/mL)	VOI_3 (counts/mL)	VOI_4 (counts/mL)	VOI_5 (counts/mL)	WKP (counts/mL)	RC
sigma = 0	T1_1	23,0		736,29	731,09	842,85	820,27	758,05	624,72	0,84	813,99	673,13	748,38	824,82	716,21	692,25	0,85
	T2_1	50,8		588,83	569,84	643,27	538,62	547,72	423,35		566,34	488,32	501,45	518,57	475,34	463,18	
	T7_1	171,7		97,8	107,88	101,12	85,74	97,55	71,03		93,43	106,16	93,68	89,27	85,32	70,29	
Sigma = 3	T1_1			681,35	698,33	793,86	766,41	711,74	624,72		776,03	628,69	704,48	782,92	700,88	692,25	
	T2_1			522,49	539,25	610,52	517,14	518,47	423,35		547,85	469,61	474,84	494,43	449,76	463,18	
	T7_1			92,84	100,97	96,18	80,64	91,07	71,03		86,73	99,19	87,33	84,97	80,08	70,29	
sigma = 4	T1_1			644,05	675,84	761,1	731,19	680,01	624,72		748,5	599,51	673,83	754,35	690,18	692,25	
	T2_1			497,49	518,76	587,1	500,41	498,77	423,35		532,12	455,16	457	478,74	432,37	463,18	
	T7_1			89,34	96,01	92,68	77,77	86,76	71,03		82,46	94,48	83,33	82,6	76,55	70,29	
sigma = 5	T1_1			602,85	650,28	724,54	692,97	644,47	624,72		716,18	567,99	638,8	772,63	677,71	692,25	
	T2_1			469,19	496,06	559,89	479,9	476,79	423,35		511,61	437,1	437,26	461,8	413,28	463,18	
	T7_1			85,32	90,31	88,65	74,95	81,19	71,03		77,85	89,34	79,13	80,48	72,67	70,29	
sigma = 6	T1_1			560,33	622,55	685,99	653,68	607,12	624,72		680,64	536,02	601,27	689,41	663,94	692,25	
	T2_1			439,14	472,27	530,16	456,46	453,52	423,35		487,17	416,14	416,67	444,62	393,61	463,18	
	T7_1			80,79	84,28	84,29	72,29	76,74	71,03		73,1	84,08	74,92	78,74	68,67	70,29	
Sigma = 7	T1_1			518,54	593,37	647,05	614,83	569,62	624,72		643,52	505,06	563,03	656,05	649,24	692,25	
	T2_1			408,82	448,2	499,26	431,17	429,76	423,35		460,14	393,29	396,1	427,95	374,17	463,18	
	T7_1			76,45	78,26	79,8	69,76	71,48	71,03		68,4	78,99	70,84	77,36	64,72	70,29	
Sigma = 8	T1_1			478,83	563,31	608,81	577,3	533,12	624,72		606,23	475,89	525,45	623,47	633,77	692,25	
	T2_1			379,32	424,38	468,26	405,03	405,99	423,35		431,98	369,55	376,14	412,18	355,39	463,18	
	T7_1			76,45	78,26	79,8	69,76	71,48	71,03		68,4	78,99	70,84	77,36	64,72	70,29	
Sigma = 9	T1_1			441,92	532,85	571,92	541,58	498,33	624,72		569,8	448,81	489,45	592,23	617,5	692,25	
	T2_1			351,38	401,1	437,94	378,91	382,58	423,35		403,93	345,82	357,1	397,36	337,48	463,18	
	T7_1			67,41	67,08	71	64,87	61,39	71,03		59,57	69,82	63,43	75,16	57,36	70,29	
Sigma = 12	T1_1			349,84	443,79	472,06	446,75	406,92	624,72		471,44	379,58	395,09	508,59	563,09	692,25	
	T2_1			279,69	336,13	355,27	306,05	316,82	423,35		328,01	280,34	306,4	356,92	289,2	463,18	
	T7_1			55,18	53,39	59,2	57,34	48,66	71,03		48,64	58,85	54,59	71,7	48,03	70,29	

Table S54 shows the activity concentrations measured using WKP and with 4 mL small VOI methods on SPECTs images post-filtered with Gaussian filter (0-12 mm) for patient number 18. Whereas, RC represented patient specific recovery coefficient of right and the left kidneys, respectively.

Post-filtering (mm)	Data	Time p.i. (h)	Inj Act (MBq)	Right Kidney							Left Kidney						
			7252	VOI_1 (counts/mL)	VOI_2 (counts/mL)	VOI_3 (counts/mL)	VOI_4 (counts/mL)	VOI_5 (counts/mL)	WKP (counts/mL)	RC	VOI_1 (counts/mL)	VOI_2 (counts/mL)	VOI_3 (counts/mL)	VOI_4 (counts/mL)	VOI_5 (counts/mL)	WKP (counts/mL)	RC
sigma = 0	T1_1	22,7		993,42	1074,37	1155,41	900,7	889,32	780,59	0,85	942,91	1282,5	1053,6	948,51	1019,52	776,11	0,84
	T2_1	50,8		799,22	712,5	698,28	633,07	664,99	522,33		573,32	637,75	774,99	610,25	560,16	505,82	
	T7_1	172,2		169,03	138,73	125,08	93,15	108,65	93,34		133,69	203,71	136,11	153,45	82,25	109,71	
Sigma = 3	T1_1			943,84	1017,95	1095,55	869,39	861,02	780,59		888,98	1190,58	1000,62	899,62	977,66	776,11	
	T2_1			763,75	689,02	672,01	607,5	616,16	522,33		555,78	619,11	739,41	580,97	542,86	505,82	
	T7_1			149,99	131,43	115,09	91,29	107,24	93,34		125,37	184,54	128,26	149,92	79,49	109,71	
sigma = 4	T1_1			908,92	979,91	1052,95	847,46	841,01	780,59		850,94	1131,07	964,5	861,42	947,74	776,11	
	T2_1			736,4	668,69	651,85	588,89	583,49	522,33		541,66	605,16	713,88	559,97	527,51	505,82	
	T7_1			138,38	126,36	109,05	90,35	106,22	93,34		120,13	172,81	123,38	147,54	77,58	109,71	
sigma = 5	T1_1			868,86	937,45	1003,61	821,63	817,79	780,59		807,77	1067,18	924,1	814,79	912,68	776,11	
	T2_1			703,47	642,53	627,67	566,77	547,31	522,33		523,94	588,55	684,15	535,63	507,13	505,82	
	T7_1			126,87	120,64	102,84	89,81	105,07	93,34		114,66	160,93	118,21	144,97	75,38	109,71	
sigma = 6	T1_1			825,87	892,95	950,18	792,39	792,08	780,59		762,26	1002,46	881,34	762,63	874	776,11	
	T2_1			667,07	612,06	600,98	541,95	509,55	522,33		503,55	570,21	651,72	509,3	482,54	505,82	
	T7_1			116,17	114,56	96,75	89,68	103,97	93,34		109,31	149,62	112,94	142,34	72,95	109,71	
Sigma = 7	T1_1			781,99	848,54	895,22	760,42	764,61	780,59		716,93	939,48	837,69	708,23	833,33	776,11	
	T2_1			629,31	579,1	573,25	515,41	471,95	522,33		481,7	550,99	618,06	482,31	455,11	505,82	
	T7_1			106,59	108,41	90,94	89,85	103,08	93,34		104,34	139,27	107,64	139,68	70,34	109,71	
Sigma = 8	T1_1			738,73	805,72	840,75	726,49	736,06	780,59		673,56	879,6	794,08	654,37	792,02	776,11	
	T2_1			591,86	545,24	545,64	488,06	435,74	522,33		459,51	531,53	584,34	455,63	426,33	505,82	
	T7_1			98,09	102,41	85,45	90,14	102,43	93,34		99,88	129,96	102,35	136,89	67,6	109,71	
Sigma = 9	T1_1			697,04	765,31	788,1	691,44	707,06	780,59		633,22	823,4	751,09	603,06	751,14	776,11	
	T2_1			555,82	511,72	518,97	460,7	401,73	522,33		437,81	512,17	551,35	429,87	397,5	505,82	
	T7_1			90,56	96,72	80,28	90,34	101,96	93,34		95,96	121,62	97,09	133,89	64,75	109,71	
Sigma = 12	T1_1			584,88	660,01	647,37	587,07	622,74	780,59		532,96	677,08	629,37	472,85	636,67	776,11	
	T2_1			460,39	419,88	447,7	383,69	315,91	522,33		379,77	455,38	461,03	360,11	318,74	505,82	
	T7_1			72,39	82,34	66,63	89,31	100,26	93,34		86,97	101,19	81,99	123,08	56,1	109,71	
